# Opposing regulation of endolysosomal pathways by long-acting nanoformulated antiretroviral therapy and HIV-1 in human macrophages

**DOI:** 10.1186/s12977-014-0133-5

**Published:** 2015-01-22

**Authors:** Mariluz Araínga, Dongwei Guo, Jayme Wiederin, Pawel Ciborowski, JoEllyn McMillan, Howard E Gendelman

**Affiliations:** Departments of Pharmacology and Experimental Neuroscience, University of Nebraska Medical Center, 985880 Nebraska Medical Center, Omaha, NE 68198-5880 USA; Pharmaceutical Sciences, University of Nebraska Medical Center, Omaha, NE 68198-5880 USA

**Keywords:** Macrophages, HIV-1, Proteomics, NanoART, Endocytic pathways, Rab proteins

## Abstract

**Background:**

Long-acting nanoformulated antiretroviral therapy (nanoART) is designed to improve patient regimen adherence, reduce systemic drug toxicities, and facilitate clearance of human immunodeficiency virus type one (HIV-1) infection. While nanoART establishes drug depots within recycling and late monocyte-macrophage endosomes, whether or not this provides a strategic advantage towards viral elimination has not been elucidated.

**Results:**

We applied quantitative SWATH-MS proteomics and cell profiling to nanoparticle atazanavir (nanoATV)-treated and HIV-1 infected human monocyte-derived macrophages (MDM). Native ATV and uninfected cells served as controls. Both HIV-1 and nanoATV engaged endolysosomal trafficking for assembly and depot formation, respectively. Notably, the pathways were deregulated in opposing manners by the virus and the nanoATV, likely by viral clearance. Paired-sample z-scores, of the proteomic data sets, showed up- and down- regulation of Rab-linked endolysosomal proteins. NanoART and native ATV treated uninfected cells showed limited effects. The data was confirmed by Western blot. DAVID and KEGG bioinformatics analyses of proteomic data showed relationships between secretory, mobility and phagocytic cell functions and virus and particle trafficking.

**Conclusions:**

We posit that modulation of endolysosomal pathways by antiretroviral nanoparticles provides a strategic path to combat HIV infection.

**Electronic supplementary material:**

The online version of this article (doi:10.1186/s12977-014-0133-5) contains supplementary material, which is available to authorized users.

## Background

Long acting nanoformulated antiretroviral therapy (nanoART) is emerging as an important part of the treatment armamentarium for human immunodeficiency virus type one (HIV-1) infection [1-4]. While our prior studies defined both a platform for drug delivery and the trafficking mechanisms operative for nanoART in monocyte-macrophages, how these cells can be harnessed as drug vehicles for improved antiretroviral responses has not been realized [5-9]. Indeed, human monocyte-derived macrophages (MDM) serve as nanoART carriers extending ART half-life and drug stability [10-12]. Such cell-based drug delivery strategies may also decrease systemic drug toxicities [13,14]. We posit that endolysosomal pathways can serve as Trojan horses for viral persistence or as vehicles for its elimination. If correct, facilitated viral replication and the means to eliminate it may occur at identical subcellular locales. The operative nanoART response would facilitate drug delivery by bringing the medicine to the site of viral replication and assembly within mononuclear phagocytes (MP; monocytes, macrophages and dendritic cells). To investigate this apparent mechanistic paradox, functional proteomic tests were employed to uncover how drug particles affect the HIV-1 replication cycle beyond nanoART activity.

The intracellular trafficking pathways held by the virus and nanoART were investigated by Sequential Windowed data independent Acquisition of the Total High-resolution Mass Spectra (SWATH-MS) profiling. This technique was applied to obtain a broader picture of complex nanoART-HIV interactions. The method was previously employed in our laboratory an others to identify and quantify cellular peptides on a larger scale [15-19]. While past transcriptomic and proteomic analyses were applied to study virus-cell interactions [16-19], they have failed to uncover key proteins affected by targeted antiretroviral treatments. Herein, we identified deregulated cellular proteins affected by nanoatazanavir (nanoATV) in HIV-1-infected MDM. Comparison was made between nanoformulated and native ATV treatments as related to the proteomic effects induced by HIV-1. Common cellular proteins with coordinated molecular, biochemical and biological functions were altered in virus-infected and nanoATV treated cells. These were linked to phagosome signalling pathways clearly associated with the endosomal and lysosomal compartments. Specifically, opposing expressions of Rab5 and −7 and LAMP1 were seen in HIV-1 infected and nanoATV-treated cells. Notably, the downregulation of late and recycling endosomes and LAMP1 indicated that pathways that could be employed, in measure, for viral assembly and nanoparticle lysosomal degradation were affected. Through cross validation of proteomics, cell biology and protein chemistry, our data provide novel insights into how nanoART facilitates viral clearance while establishing long-lived cell-based depots different from native drug. These works represent a previously unknown mechanism for how long-acting nanoART provides a strategic advantage to combat viral infection.

## Results

### Proteomics analyses of HIV-1 infected MDM

**Figure 1:**
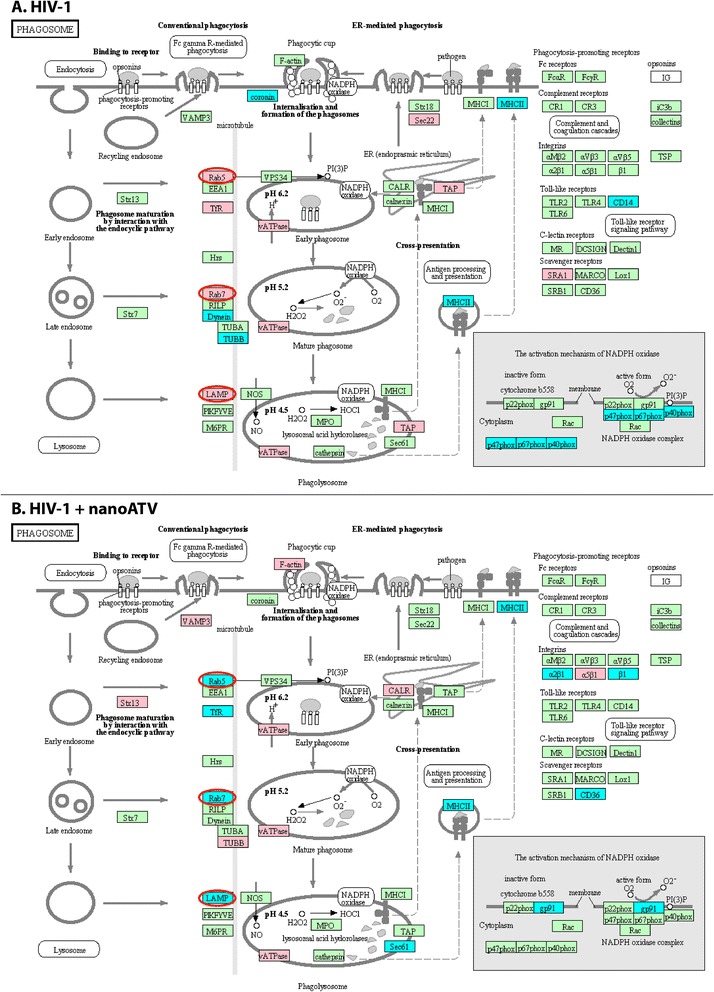
**Schematic representation of the MDM phagosome network identified in HIV-1-infected (A) and HIV-1-infected and nanoATV treated cells (B).** Proteins identified were compared against control uninfected MDM cultures (p < 0.05). The acquired profiles were analyzed through the bioinformatics program using a comprehensive set of functional annotation tools to uncover biological data sets behind the uncovered list of genes. Data for Annotation, Visualization and Integrated Discovery (DAVID) facilitated the linked sets of enriched functional-related protein groups. This tool was employed to identify enriched biological processes among the expressed proteins. Gene Ontology terms were used to identify related pathways with the assistance of the Kyoto Encyclopedia of Genes and Genomes (KEGG) database. The KEGG database facilitated the elucidation of the functions for the MDM as derived from the proteomic datasets. Statistical significance was determined using a *p*-value < 0.05. Proteins in red and blue, display up- and down- regulation, respectively. Proteins in green belong to the phagosome network and not deregulated by ATV treatment. The differences in protein up- and down-regulation between HIV infection alone and HIV infection with nanoATV treatment are circled in red.

**Figure 2:**
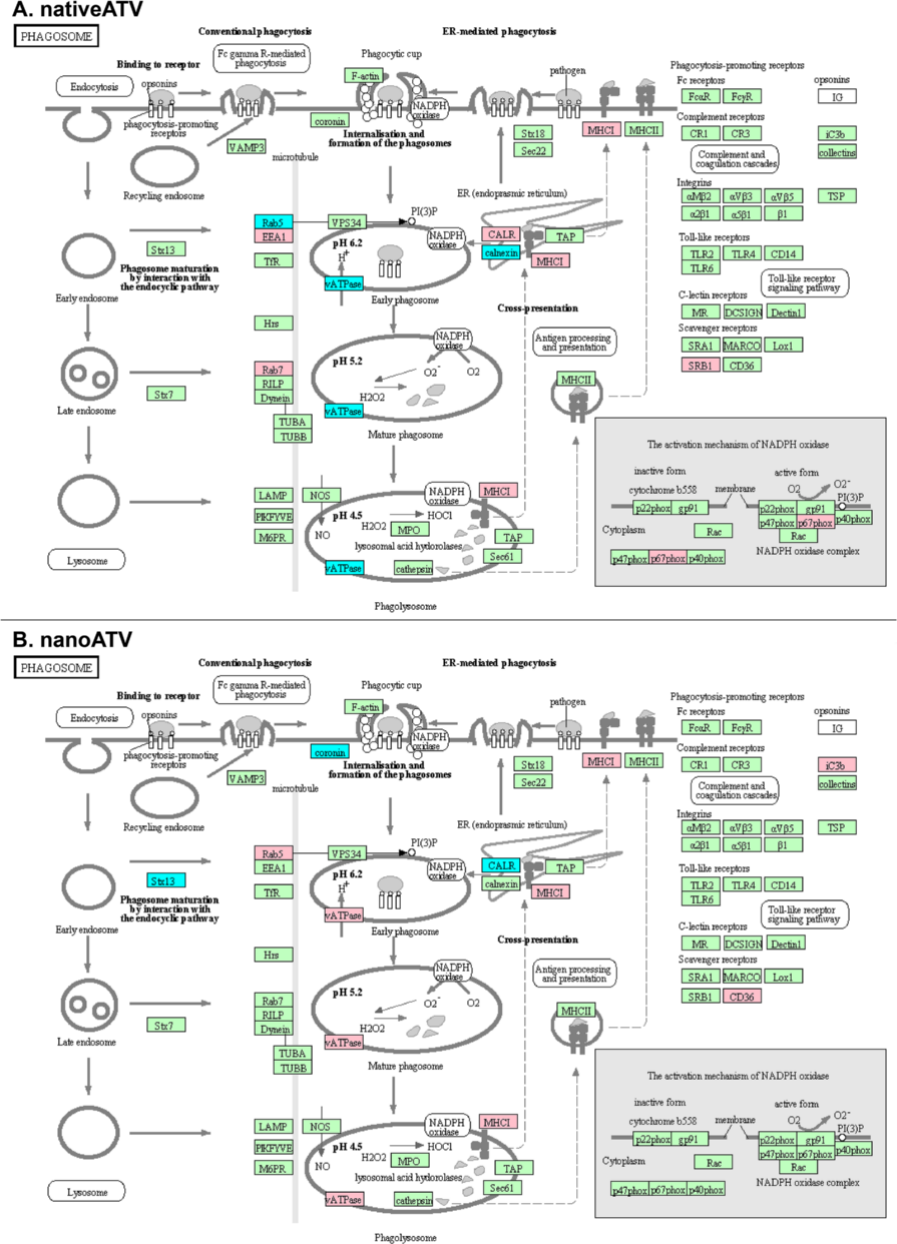
**Changes in MDM phagosome network for uninfected cells treated with native ATV (A) or nanoATV (B).** Proteins were compared to uninfected and untreated MDM controls (p < 0.05) then bioinformatics analysis performed following parallel procedures described in Figure 1. Proteins in red and blue, display up- and down- regulation, respectively. Proteins in green belong to the phagosome network but were not significantly altered by viral infection or treatment.

HIV-1 infection engages a spectrum of cellular proteins seen in specialized cell populations that support its replication [20-23]. The effect of nanoART on cell protein expression has not yet been defined, in its target macrophage. To such ends, we applied quantitative SWATH-MS proteomics followed by bioinformatics to uncover proteins deregulated by native ATV or nanoATV with or without HIV-1 infection. For these experiments MDM were first infected with HIV-1_ADA_ and four hours later medium was removed and cells were treated with 100 μM P407-ATV. Following 16 hours of drug treatment, the media was replaced with drug-free fluids and cells were harvested for proteomic tests after an additional seven days. This experimental paradigm was followed to assess the role that the antiretroviral delivery system had on the macrophage proteome during spreading viral infection. To separate the effects of the antiretroviral drug, the nanoparticle and the viral infection, separate and combined analyses of each of these were required. MDM were infected with HIV-1 at a MOI of 0.1 then treated with 100 μM native- or nanoATV. After seven days, cells were harvested and SWATH-MS was performed on whole cell lysates [15]. Because of the expansive proteomic data sets and analyses based on the biological response variables amongst the nanoATV, ATV and HIV-1 treatments the data are presented in 7 additional files and two figures. Figure 1 illustrates differences in protein expression between HIV-1 infected cells with or without nanoART as compared to controls (uninfected-untreated MDM) and Figure 2 illustrates differences in protein expression between uninfected cells treated with native- and nanoATV compared to controls. Quantitative profiling identified 527 significantly changed MDM proteins following HIV-1 infection. These were up- or down-regulated (p < 0.05) and were assessed by paired-samples z-scores (Additional file 1). The numbers of proteins exhibiting changed expression in HIV-1-infected cells were greater than in replicate infected cells treated with nanoATV, 527 *versus* 376 respectively (Additional file 2A). Up- and down- regulated proteins in HIV-1 infected cells were 41 and 59% of total (n = 216 and 311, respectively). In contrast for nanoATV-treated HIV-1-infected MDM, up- and down-regulated proteins were 59 and 41% of total (n = 222 and 154, respectively). Uninfected cells treated with nanoATV had fewer deregulated proteins (n = 195) compared to the other groups (Additional file 1). The proteins uncovered engaged the PANTHER database which sorted the deregulated proteins by classes. This illustrated the relative numbers of proteins in each class for the HIV-1-infected and infected and nanoATV-treated cells (Additional file 2B). This showed that the number of deregulated proteins in HIV-1-infected *versus* infected and nanoATV-treated cells was greater for each of the classes (nucleic acid binding, hydrolase, transferase, protease, signalling molecule, transporter, transcription factors and ligases). These results demonstrate that the deregulation of cellular proteins by HIV-1 infection can be altered by nanoATV treatment. To uncover the function of the classes of proteins deregulated by HIV infection we examined the functional categories by PANTHER classification. These data are based on Gene Ontology annotations (GO molecular function, GO biological processes and GO cellular component). Additional file 3A shows the proteins sorted according to molecular function with the percentages of total deregulated proteins classified for subgroups. Common proteins were separated based on binding (28 to 31%), enzyme regulator (5%) and transporter (3-4%) activities between the nanoATV, HIV-1 and HIV-1 and nanoATV treated MDM. Proteins included Rab5, −7 (GDP/GTP and protein binding) and LAMP1 (enzyme and protein binding). The classification revealed enrichment for metabolic, cellular, localization, regulation and cellular organizational cellular processes. These were the principal categories or protein sets (Additional file 3B). The relative ratios for the proteins were similar amongst groups. GO for cellular component showed that deregulated proteins sorted by cell organelle and macromolecular complex (Additional file 3C). Note that such groupings were common to HIV-1 and HIV-1 and nanoATV treated cells. The data demonstrated that HIV-1 and HIV-1 and nanoATV, as compared to control, affect similar cellular processes. However, the numbers of proteins in each were reduced following infection and nanoATV treatment.

### KEGG pathway analyses for HIV-infected MDM

**Figure 3:**
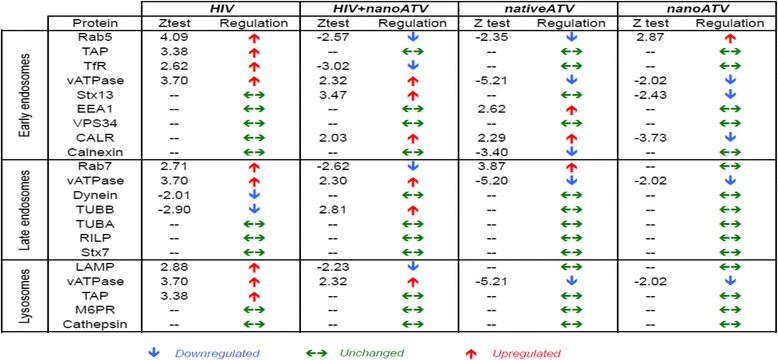
**Endolysosomal proteins in HIV-1, HIV + nanoATV, nanoATV and native ATV treated MDM.**

The proteins relative to functional pathways were further investigated using the KEGG database, which indicated phagosomes as one of the main pathways related to HIV-1 infection and nanoATV treatment. *First* we identified the role of HIV-1 infection as compared to HIV-1 infected nanoART-treated MDM on the phagosome network (Figure 1A and B, respectively). Few proteins were deregulated with nanoATV treatment. However, more proteins were deregulated during HIV-1 infection and nanoATV treatment. Notably, there was an opposite regulation for proteins within the phagosome and endosomal compartment between HIV-1-infected and HIV-1-infected and nanoATV-treated MDM. Up-regulation of Rab5 and −7 proteins was observed in HIV-1 infected cells; in contrast these same proteins were down-regulated in nanoATV-treated HIV-1 infected cells (pink = increased expression, blue = decreased expression). A similar pattern for LAMP1 was also observed. Moreover, DAVID functional enrichment clustering gave similar enrichment results for lysosomes in HIV-1-infected and HIV-1-infected and nanoATV-treated cells by filtering the data sets at a *P* value <0.01 (Additional files 4, 5, 6). There was a down-regulation of endosomal and lysosomal proteins in the uninfected cells treated with native ATV (Figure 2A) or nanoATV. The latter showed few down-regulated proteins in parallel endosomal compartments (Figure 2B). HIV-1 and native ATV treatment was similar to HIV-1 alone (i.e. high number of altered proteins). There were less numbers of oppositely regulated proteins compared to HIV and nanoATV (data not shown). A composite of these protein network changes are summarized in Figure 3.

### Protein-protein interaction networks

To elucidate the operative and dynamic biological processes, methods depicting changes of protein interaction networks are needed. Here we used our data from SWATH-MS and applied it to the STRING bioinformatics tool to determine the dynamics of the protein interaction after HIV-1-infection with or without nanoATV treatment. Differentially expressed proteins in infected or infected and treated MDM were identified using a P-value <0.05, and protein-protein interaction networks were constructed. The results of the networking reproduced a consistently high number of altered cellular proteins by HIV-1 (Additional file 7A). The complex changes and interactions during HIV-1 infection are clearly visualized and, more importantly, the reduced complexity is clear when the infected cells are treated with nanoATV (Additional file 7B). These dynamic changes following HIV-1 infection and nanoATV treatment provide evidence of the importance of antiretroviral therapy to control protein-protein interaction networks.

### Antiretroviral activities of native and nanoformulated ATV

**Figure 4:**
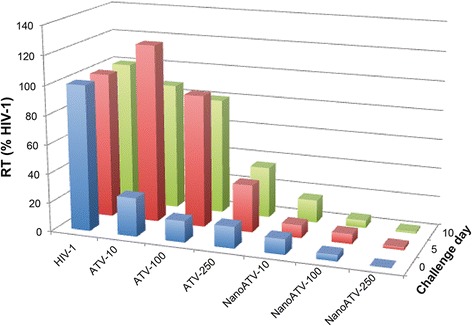
**NanoATV treatment affects HIV-1 reverse transcriptase (RT) activity.** HIV-1 RT activity was determined in treated (native ATV or nanoATV) MDM followed by HIV-1 infection at days 0, 5 or 10. HIV-1 infected cells without any treatment served as a positive control for RT activity. All samples were collected after 7 days of viral infection. Results shown are the mean of 5 replicates.

To confirm the antiretroviral activity of nanoATV treatment HIV-1 reverse transcriptase (RT) activity was determined in HIV-1-infected human monocyte-derived macrophages (MDM) treated with either native- or nanoATV. Cells were treated with 10, 100 or 250 μM of native- or nanoATV for 16 hours. At this time the medium was removed, cells were washed 3 times with phosphate buffered saline and fresh medium without drug was added prior to HIV-1_ADA_ challenge at a multiplicity of infection (MOI) of 0.1 at days 0, 5 and 10 after treatment. Infected cells were cultured for an additional 7 days and RT activity in the culture medium was determined. Significant differences were found between cells treated with native- or nanoATV. For native ATV treated cells, RT activity was suppressed only in the day 0 infection group, at all treatment concentrations. At 10 and 100 μM little antiviral suppression was observed in the days 5 and 10 infection groups. In contrast, for cells treated with nanoATV, RT activity was suppressed to less than 20% HIV-1 positive control with all treatment concentrations and at all infection days (Figure 4).

### Endolysosomal proteins deregulated HIV-1 and nanoATV

**Figure 5:**
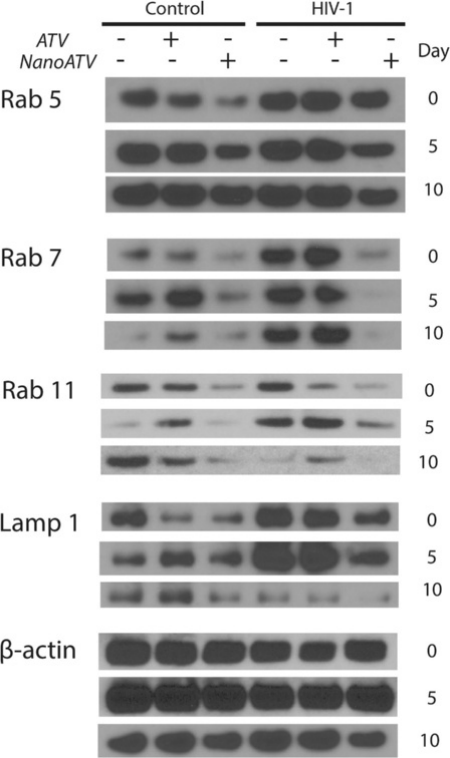
**NanoATV and HIV-1 endosomal protein regulation.** Western blot of Rab5, −7, −11, LAMP1 and β-actin was performed in cell lysates from MDM treated with native ATV or nanoATV and infected with HIV-1 at day 0, 5 or 10 post-drug treatment then incubated for 7 days. Uninfected cells and infected cells without drug treatment served as negative and positive controls for differential expression of cellular proteins during HIV-1 infection. Blots shown are from one donor and experiment, and equivalent to two independent experiments performed.

**Figure 6:**
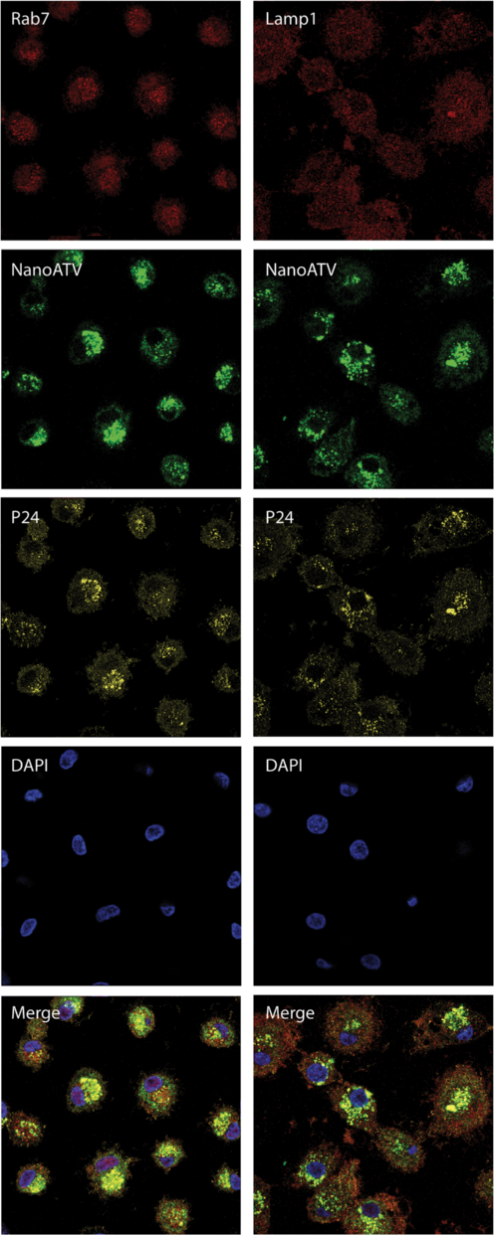
**Subcellular localization of nanoATV, HIV-1 and endolysosomal proteins.** Cellular localization of Rab7 or LAMP1 endosomal compartments (red), HIV-1p24 (yellow) and nanoATV (green) are shown by confocal microscopy. Cell nuclei were stained with DAPI (blue). Merged images showed the co-localization of all proteins. Fluorescence images were acquired with a LSM 510 confocal microscopy, 400x magnification.

We selected the proteins from the KEGG pathway related to phagosome and endosomal compartment which were oppositely regulated by HIV-1 and HIV-1/nanoATV, (Rab 5, −7, −11 and LAMP 1), for validation of the proteomics analysis. Protein expression of Rab 5, −7, −11 and LAMP1 was determined by Western blot. As shown in Figure 5, there was a down-regulation in Rab 5, −7, −11 and LAMP1 protein expression in nanoATV-treated infected cells group. This effect was greater than that seen with native ATV treatment, validating the KEGG analyses, and was time dependent; highlighting the dynamic nature of endosomal trafficking based on macrophage differentiation, viral infection and nanoATV treatments. Notably, assay of Rab protein levels, at multiple days following of HIV-1 infection and antiretroviral treatment revealed that nanoATV while inducing a significant down-regulation of endosomal and lysosomal proteins the effects paralleled what was observed in uninfected cells. Moreover, the downregulation was significant as it was sustained over 10 days. To better assess the potential relationships between the virus, Rab protein and nanoART we used immunofluorescence to visualize if co-localization of Rab7 or LAMP1, HIV-1 p24 and nanoATV could occur. Immunofluorescence co-localization (Figure 6) demonstrated that HIV-1 p24 (yellow), Rab7 or LAMP1 cellular proteins (red) and nanoATV (green) were in the identical cellular locale. These results highlight the fact that the endosomal trafficking routes taken by the virus and the nanoATV are identical. Most importantly the results support the idea that HIV-1 and nanoATV while present in the identical subcellular locale influence endosomal trafficking in opposite ways.

### Cytokine profile for HIV-1 and nanoATV

**Figure 7:**
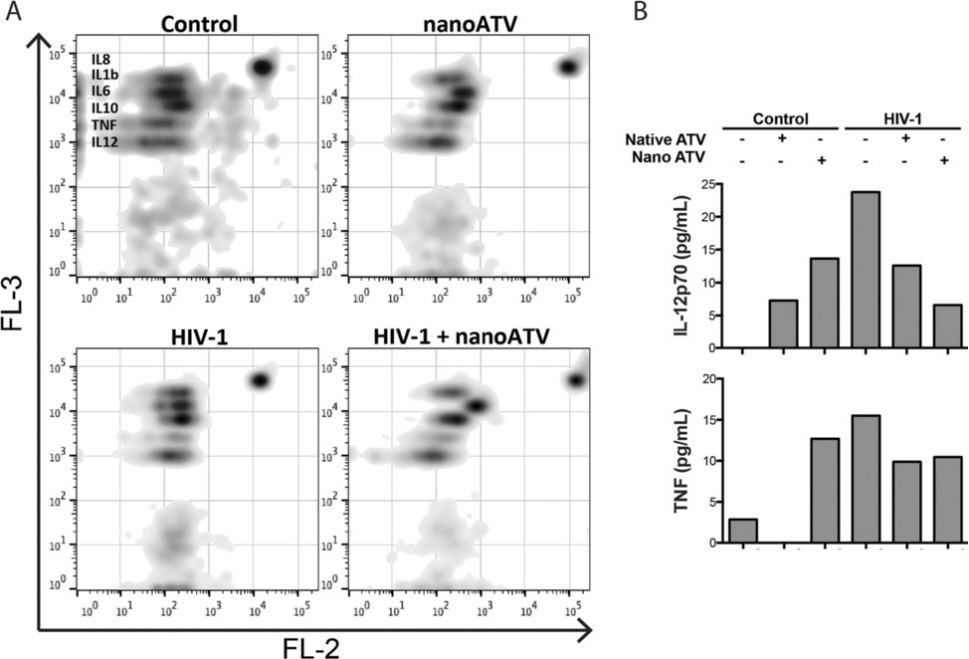
**NanoATV regulation of cytokine profiles in HIV-1 infected MDM.** MDM were treated with 100 μM native ATV or nanoATV and infected with HIV-1 at day 0, 5 or 10 post-drug treatment. Untreated, uninfected cells were used as controls. After 24 hours of viral infection, cell culture media were collected and analyzed using a cytokine bead array. **(A)** Density plots show expression of IL-12, TNF, IL-10, IL-6, IL-1β and IL-8 for control, treated, HIV-1 infected and treated infected MDM. Cytokine levels were detected by FACSArray cytometer and data was plotted using FlowJo (version 10.7) software. **(B)** Levels of IL-12 and TNF after treatment and infection are shown. Data are analyzed using FCAP software and values of cytokine expression were expressed as pg/mL.

To assess the activation state of the MDM, cytokine production was determined in HIV-1 infected cells with or without nanoATV treatment. Cell culture media from nanoATV treated and untreated HIV-1 infected and uninfected MDM were incubated with capture beads for IL-12, TNF, IL-10, IL-6, IL-1β and IL-8 and a detection fluorochrome. Acquisition was performed by FACSArray cytometry. IL-12 and TNF were increased in HIV-1 infected MDM. However, when the infected cells were treated with nanoATV cytokine levels were reduced (Figure 7), implying a positive correlation with endosomal and lysosomal proteins expression in our analysis mentioned above. NanoATV treated infected macrophages also expressed higher levels of IL-6 and IL-8, compared to infected and uninfected cells. There were no significant changes for IL-10 and IL-1β (data not shown). These results showed a negative correlation in the expression of IL-12 and TNF between treated and untreated HIV-1 infected MDM, suggesting a role for nanoATV as a regulator of pro-inflammatory cytokines.

## Discussion

While it is well known that HIV-1 alters cellular nucleic acid binding and regulatory protein functions affecting its transcription and translation [15,24-26] how such virus-host cell interactions are altered when viral replication is attenuated by nanoART is not understood [11]. The effect of nanoART alone was also investigated here. To this end, we used functional proteomics, cell biology and protein chemistry to investigate potential interactions between the virus, the host cell and nanoART to elucidate how metabolic and signaling pathways can be engaged to both support viral replication and, at the same time, affect its elimination. Endolysosomal pathways were uncovered and found to be antagonistic in HIV-1 infected cells with nanoATV treatment. Notably, the results highlight how the cell can be manipulated to either facilitate or inhibit viral growth.

The data also serves to highlight unique cellular processes engaged in both the viral replication cycle and the means to attenuate it. Within an infected cell, virions are formed in association with the cellular membrane. Initial investigations of HIV-1 assembly in macrophages were done through electron microscopy studies and suggested that new virions were formed from the limiting membrane of a late endosomal compartment that was linked to vesicle formations as is known to occur in multi-vesicular bodies (MVB) but not at the plasma membrane known to be operative in T cells [27]. The linkage between Endosomal Sorting Complex Required for Transport (ESCRT), MVB biogenesis and viral budding is well known. In past years it was thought that HIV-1 budding is linked to ESCRT through the presence of late endosomal markers associated with macrophage-derived virions. However, the model has now been re-examined with several recent reports showing that the viral compartment has a neutral pH and can be connected to the plasma membrane by micro-channels [28-33]. We posit that the virion trafficking and viral budding can be independent but not mutually exclusive pathways and showed that both are likely operative. *First*, we now show through cross validations of proteomic, cell biology and protein chemistry that the endolysosomal machinery is significantly deregulated by HIV-1 infection and in an opposite manner by nanoART treatment. *Second*, there is a close association between endosomal-linked pathways that include Rab5, −7 and −11 and viral infection. *Third*, the fact that this pathway is conversely down regulated by HIV-1 infection likely reflects that the ability of the virus to hijack ESCRT is augmented by the drug nanoparticles. Such a theory was previously put forward in our own past works [11]. In regards to assembly and intracellular accumulation of progeny HIV-1 we need not discount the elegant work performed by immunofluorescence microscopy and immunoelectron microscopy that the organelles of an internally sequestered plasma membrane domain are divergent from endosomes. Both pathways can be operative in this scenario and are not mutually exclusive from one another. Nonetheless, there is little question that virions are endosomal-associated.

Progeny virions are pulled down that are associated with these proteins and reverse transcriptase activities are reduced significantly in parallel structures. While we did not study the tetraspanins CD81, CD9, and CD53, their regulation in phagocytosis or intracellular trafficking is appreciated [34]. It is noted that CD81 is linked to activation of mononuclear phagocytes, notably microglia [35]. Moreover, they are also involved in the formation of multinucleated giant cells [36] an additional major feature of viral infection in macrophages. Rab proteins function to evade degradation and direct transport to intracellular locations and utilize host vesicles to affect a stable intracellular niche for microbial stability and longevity [37]. Thus, it is not surprising that HIV-1 would induce the Rab pathways. While in uninfected macrophages the proteins are at the cell surface and in intracellular vacuole-like structures with a complex content of vesicles and interconnected membranes, these compartments are in a dynamic state within the cell and strongly regulated by HIV-1. While we acknowledge that endosome markers could be recruited to the viral structures and incorporated into virions the dynamic process of the virus and the macrophage transcends progeny virion assembly and includes viral trafficking and transport mechanisms. Such observations combined with a broader theory of the complexity of virus-cell interactions in the infected macrophages heralds the notion that multiple events are operative for virion assembly and persistence [38].

NanoART enters the macrophages primarily through clathrin-mediated pathways and is then stored in endocytic compartments. This provides a protected environment for release of the drug to sites of viral growth. Compared with nanoART, the non-formulated native drug didn’t show similar trafficking behavior since less endolysosomal proteins were found related to native drug treatment. On the one hand, amorphous native drug can hardly be taken or stored by macrophage; however, native drug cannot be internalized or carried by subcellular compartments for intracellular transportation. Subcellular distribution of nanoformulated ART is in late and recycling endosomal compartments. These same compartments serve as drug depots. Since late endosomes are sites of viral assembly, nanoART stored within such compartments can retain significant antiretroviral activity. This was clearly demonstrated by the significant reductions in HIV-1 RT activity previously observed in isolated endosomal compartments in nanoATV treated HIV-1 infected MDM.

All together, the current studies suggest a mechanism whereby the endolysosomal pathway is harnessed for HIV-1 viral replication and this same pathway may provide a means for its elimination. Indeed, while it is known that HIV-1 traffics through Rab5, −7, and −11 endosomal compartments how such early, late, and recycling endosomal pools regulate stages of the viral life cycle are not understood [39]. Such an intersection though is believed critical to the viral life cycle as the functions of the compartments serve to maintain cell homeostasis and protein transport [40]. It has been reported that Rab5 regulates clathrin-mediated endocytosis from the plasma membrane to early endosome pools and serves as an intersection point for proteins sorted to undergo degradation through Rab7-dependent late endosome and lysosomal routes or be sorted back to the plasma membrane through Rab11-dependent recycling pathways [39]. The ability of the macrophage to overcome such degradation events at the subcellular level underlie its abilities to persistent in its macrophage reservoir [39]. Moreover, it is well known and accepted that Rab proteins function to evade degradation and direct transport to intracellular locations and utilize host vesicles to affect a stable intracellular niche for microbial stability and longevity. Similar mechanisms certainly parallel the persistence of nanoART and sustained drug depots for extended time periods. Notably, proteomic tests revealed a large number of proteins deregulated by HIV-1 infection and these were the same protein sets also affected by nanoART. The protein sets included those affecting nucleic acid binding, hydrolase and enzyme activities, oxidoreductase responses, and the cellular cytoskeletal backbone. These were all characterized by GO molecular function that placed Rab5 and −7 proteins as those engaged in GTP catabolic processes, endocytosis and small GTPase-mediated signal transduction pathways. The molecular functions for both endosomal-linked proteins are GDP/GTPase activity and protein binding [41]. LAMP1 is associated with autophagy, the establishment of protein localization to cell organelles, golgi to lysosome transport, protein transport along microtubules and regulation of natural killer cell degranulation cytotoxic activities. For the cellular component GO classification, LAMP1 is located at the late endosome, lysosome, multivesicular body and vesicular exosome. It is included in the group of enzyme and protein binding molecular function protein sets. Indeed, as described by GO information, Rab family members are small, RAS-related GTP-binding proteins that regulate vesicular transport. Each Rab targets multiple proteins that act in exocytic and endocytic pathways.

An important finding in the current study was that Rab5, −7 and −11 and LAMP1 were significantly upregulated in expression following HIV-1 infection. HIV-1 could induce those proteins linked to endosomes. While endosome markers could be recruited to the viral structures and incorporated into virions, the dynamic process of the virus and the macrophage transcends progeny virion assembly but also viral trafficking and transport mechanisms also strongly affected during the dynamic course of viral infection. Interestingly, Rab 5, −7, −11 and LAMP1 deregulation in infected MDM was reversed, in part, by nanoATV. The extent of protein deregulation in infected MDM was reduced by nanoATV. As shown in some studies, Rab5 has a role in endocytosis and post-endocytic trafficking [42]. Its activation promotes focal adhesion disassembly, migration and invasiveness in tumor cells [43] and its knockdown decreases cell motility and invasion by an integrin-mediated signaling pathway [44]. Moreover, it has been indicated that Rab5, −7 and −11, affect RGS4 trafficking through plasma membrane recycling or endosomes [41] and are used by the drug particles and the virus in a coordinated manner. This was seen for other viral infections such as hepatitis B virus, which can affect Rab5 and −7 expressions and use pathways for viral transport from early to mature endosomes. This is a required step in the viral life cycle [45]. Similarly, in our study, following endocytosis HIV-1 travels through the complex endocytic pathway networks to reach the nucleus and initiate its replication and as such support the notion that endosomal proteins play a critical role in the viral life cycle.

Interestingly, Rab5, −7 and −11 and LAMP1 were down-regulated in HIV-1 and nanoATV-treated cells. This opposite regulation between HIV-1 and nanoATV in regulation of endosomal proteins is likely important in that cellular trafficking pathways may also be involved in the release of infectious progeny virus. As such late endosome-associated Rab7A is known to be required for HIV-1 propagation, regulation of Env processing and the incorporation of mature Env glycoproteins into viral particle [46]. In addition Rab7A promotes Vpu interaction with BST2/tetherin to facilitate HIV-1 release [47]. This may also be operative for nanoparticle viral interactions and suggests that in the present study nanoATV may disrupt mechanisms of critical cellular protein-protein interactions harnessed during the viral life cycle to perpetuate its growth. In other studies, silencing the expression of Rab9 inhibited HIV-replication [48] and silencing the endogenous Rab11a GTPase expression could destabilize HIV-1 Gag and reduce virion production both *in vitro* and in NOD/SCID/γc−/− mice [49]. It has been well documented that Rab11 is located on pericentriolar recycling endosomes and plays a key role in regulating vesicle trafficking through recycling endosomes to the plasma membrane as well as in exocytosis [11,50,51]. Therefore, down regulation of Rab11, as shown in our study, could destabilize HIV-1 proteins that would fail to traffic through the endosomal compartments and could be redirected for degradation at the lysosomal site.

The deregulation of endosomal proteins suggests a new mechanism for viral suppression by nanoART. This includes altered expression of endosomal proteins resulting in parallel reductions in viral assembly sites. In addition, reduction of LAMP1 following nanoART treatment could reduce degradation of the nanoparticles and therefore extend the half-life of ART. Our data is corroborated by studies demonstrating co-localization of HIV-1 and endosomal and lysosomal proteins (Rab7 and LAMP1) [11,52,53]. Moreover, differences in cytokine profile expressions in untreated and nanoATV treated infected HIV-1 macrophages suggest that nanoATV down regulated the expression of pro-inflammatory cytokines. HIV-1 has been linked to the up-regulation of cytokines and in fact HIV-1 Tat upregulates IL-12 and TNF-α and -β expression in monocyte-derived dendritic cells [54,55], suggesting an advantage of nanoATV in the regulation of pro-inflammatory cytokines during HIV-1 infection. In contrast with nanoATV, the effect of native ATV was relatively lesser. Moreover, it has been reported that IL-12 up-regulates Rab7 and induces lysosomal transport. Others have reported that Rab proteins are regulated by cytokines and affect TNF secretion by activated macrophages [56-58]. These findings provide further support to link Rab, IL-12 and TNF expression. As HIV-1 virions assemble at the plasma membrane and recruit endosomes to enable particle release, nanoATV depletes endosomal/lysosomal proteins and deregulates pro-inflammatory cytokines thus controlling viral growth. Although a mechanism is now forged to bridge nanoATV activities and endosomal signaling pathways this study serves as only an entry to future investigations.

To this end, we are currently examining the possible signaling pathways deregulated by nanoATV. Altogether, we found that SWATH-MS proteomics, bioinformatics analyses and cell biology showed that nanoATV treatment of HIV-infected MDM can down-regulate the endocytic proteins in HIV-1 infected cells and thus decrease the subcellular space available for viral assembly. Through this mechanism, nanoATV has unique but real potential towards improving virus clearance. Our work articulates commonly used pathways that are engaged in common macrophage functions such as phagocytosis and vesicular trafficking that are used both by the virus and the anti-virus.

## Conclusion

HIV-1 and nanoATV deregulate cellular proteins in opposing manners. The common pathways are linked to viral assembly and are endolysosomal-linked. Rab5, -7, -11 and LAMP1 serve to coordinate molecular and biological functions of the virus and the antivirus in subcellular compartments. Alterations made by HIV-1 and nanoATV indicate that specific organelles are action sites for both. These findings provide novel insights into the role played by long acting subcellular targeted nanotherapies for combating HIV-1 infection.

## Methods

### Reagents and antibodies

ATV sulfate (Gyma Laboratories of America Inc., Westbury, NY, USA) was free based with triethylamine. Poloxamer 407 (P407) and CF568-succinimidyl ester (CF568) were purchased from Sigma-Aldrich (St. Louis, MO, USA). Human serum was obtained from Innovative Biologics (Herndon, VA, USA). Macrophage colony-stimulating factor (MCSF) was prepared from 5/9 m alpha3-18 cells (ATCC; CRL-10154) [59]. Rabbit anti-human Rab 5, −7, −11, LAMP1 and β-actin antibodies were purchased from Santa Cruz Biotechnology, Dallas, TX, USA. Alexa Fluor 594 goat anti-rabbit IgG and Alexa Fluor 647 donkey anti-mouse IgG were obtained from Life Technologies (Eugene, OR, USA).

### NanoATV manufacture and particle characterization

P407-ATV was prepared by high-pressure homogenization using an Avestin Emulsiflex C3 homogenizer (Avestin Inc; Ottawa, ON, Canada) [8,60]. CF568-labeled P407-ATV was prepared as described previously [14] using a 1: 4 (w/w) ratio of CF568-P407 and P407. Drug content of the nanosuspensions were determined by reverse phase high-performance liquid chromatography (HPLC) [60]. Particle size, polydispersity and zeta potential for the nanoparticles were determined by dynamic light scattering using a Malvern Zetasizer Nano-ZS instrument (Malvern Instruments Inc.; Westborough, MA, USA).

### Monocyte isolation, cultivation and HIV-1 Infection

Human peripheral blood monocytes were obtained by leukapheresis from HIV-1,2 and hepatitis B seronegative donors and plated at a density of 1 × 10^6^ cells/mL in Dulbecco’s modified Eagle’s medium supplemented with 10% heat-inactivated human serum, 1% glutamine, 50 μg/ml gentamicin, 10 μg/ml ciprofloxacin and 1,000 U/ml MCSF [61]. After seven days of cell differentiation, MDM were infected with HIV-1_ADA_ at a MOI of 0.1 infectious viral particles per cell. After 4 hours the medium was removed and cells were treated with 100 μM native- or nanoATV. Following 16 hours of drug treatment, the media was replaced with drug-free fluids and cells were incubated for an additional seven days [9].

### SWATH-MS

MDM samples for mass spectrometry were collected seven days after infection and drug treatment. Cells were washed with ice-cold PBS, scraped, pelleted, and stored at −80°C until processed. Cell samples from four donors were processed simultaneously. Cell pellets were re-suspended in cell lysis buffer containing 4% (w/v) SDS, 0.1 M dithiothreitol (DTT) and 0.1 M Tris–HCl. Lysates were vortexed at room temperature for 10 min and then boiled at 95°C for 5 min to denature proteins. Protein quantification was performed using the Pierce 660 nm protein assay (Thermo Scientific; Wilmington, DE, USA) following the manufacturer’s protocol. On the basis of protein quantifications, 100–200 μg of each sample was processed using filter aided sample preparation (FASP) [62-64]. Samples were denatured with urea exchange buffer (8 M urea, 0.1 Tris–HCl, pH 8.5) placed into filter cartridges (10 kDa), centrifuged and then treated with 50 mM iodoacetamide (Sigma-Aldrich). Trypsin (Promega; Madison, WI, USA) was added (2 μg/100 μg protein) and incubated at 37°C overnight on the cartridge. Eluted peptides were dried via vacuum centrifugation. Peptides were cleaned using an Oasis mixed cation exchange cartridge following manufacturer’s protocols (Waters Inc.; Milford, MA, USA) and then dried under vacuum. After processing through mixed cation exchange, peptides were subjected to further clean up using C18 Zip-Tips (EMD Millipore; Billerica, MA, USA) and dried under vacuum. Peptides were resuspended in 0.1% formic acid (Honeywell Burdick & Jackson; Muskegon, MI, USA) and quantified using NanoDrop2000 (Thermo Scientific). One μg of peptide was then prepared for SWATH-MS quantitative proteomics analysis, as previously described [15,65]. Samples used to generate the SWATH-MS spectral library were subjected to traditional, data-dependent acquisition (DDA).

### Bioinformatics

Each SWATH-MS condition (per each donor) was transformed independently of other conditions and comparisons between control condition and experimental conditions were calculated. Extracted raw data transformation was performed as described by Haverland et al. [15] The raw intensity for each protein was transformed by taking the natural log (ln) of the intensity followed by assignment of *z*-score. The *p*-value for the computed *z*-score was assigned using standard normal distribution. Functional analysis and signalling pathway representation were performed using an array of complementary, open-access bioinformatic tools. Functional annotation of the proteins differentially expressed was performed using the Database for Annotation, Visualization and Integrated Discovery (DAVID) Bioinformatics Resources (6.7) and the Protein Analysis Through Evolutionary Relationships (PANTHER) Classification System (9.0), by entering the UniProt sequence feature. The gene ontology (GO) annotations showed proteins according to Biological Processes, Molecular Functions and Cellular Components. Protein class functional analysis was obtained by PANTHER. Protein–protein interactions among all identified transcription regulators were investigated using Search Tool for the Retrieval of Interacting Genes/Proteins (STRING) (9.1) considering a confidence of greater than 0.4 (medium confidence). Unconnected proteins (orphan proteins) and unconnected satellite networks (networks which were detached from the largest network) were removed.

The complementary pathway analysis, Kyoto Encyclopedia of Genes and Genomes (KEGG) was used to determine significant pathways between experimental conditions. The KEGG pathway (71.0) for the phagosome was coloured using the KEGG mapper colour pathway tool. Green represents all proteins confidently identified and red and blue colors are assigned to up- or down-regulated proteins, respectively.

### Antiretroviral activities

HIV-1 reverse transcriptase (RT) activity was measured to assess antiretroviral efficacy in HIV-1 infected MDM. MDM were treated with 10, 100 or 250 μM native- or nanoATV for 16 hours then infected with HIV-1_ADA_ for 4 hours at a MOI of 0.1 immediately and five and 10 days after drug treatment. Following viral infection, cells were cultured for an additional seven days at which time cell media were collected for measurement of RT activity [7,9]. Briefly, in a 96-well plate, 10 μL of sample supernatants were mixed with 10 μL of solution containing 100 mM Tris–HCl (pH 7.9), 300 mM KCl, 100 mM dithiothreitol, 0.1% NP-40 and water. The reaction mixture was incubated at 37°C for 15 min and 25 μL of a solution containing 50 mM Tris–HCl (pH 7.9), 150 mM KCl, 5 mM DTT, 15 mM MgCl_2_, 0.05% NP-40, 10 μL/mL poly(A), 0.25 U/mL oligo d(T) and 10 μCi/mL ^3^H-thymidine triphosphate was added to each well; plates were incubated at 37°C for 18 hours. Following incubation, 50 μL of cold 10% TCA was added to each well, the wells were harvested onto glass microfiber filters and the filters were assessed for ^3^H-thymidine triphosphate incorporation by β-scintillation spectroscopy using a TopCount NXT (Perkin Elmer Inc.; Waltham, MA, USA).

### Western blots

Protein expressions of Rab 5, 7 and 11, LAMP-1 and Actin were detected by Western blot assays. MDM were treated with native drug or nanoATV and infected with HIV-1_ADA_ as described. Seven days after infection cells were collected and lysed using CellLytic M Cell Lysis Reagent (Sigma-Aldrich). Protein content was quantitated using the Pierce 660-nm protein assay. Ten μg of protein was separated by electrophoresis using a NuPAGE Novex 4-12% Bis-Tris gel (Life Technologies-Novex; Grand Island, NY, USA). After electrophoresis, the proteins were transferred to a PVDF membrane (BioRad Laboratories, Hercules, CA, USA) and then blocked with 5% non-fat dry milk in PBS and 0.1% Tween-20 (PBST). Membranes were probed with primary antibodies for Rab5, Rab7, Rab11 or LAMP-1 and β-actin (Santa Cruz Biotechnology) followed by horseradish peroxidase-conjugated secondary antibody (Life Technologies-Novex). Proteins were detected using the SuperSignal West Pico Chemiluminescent substrate kit (Thermo Scientific) [7,11,66].

### Immunofluorescence and confocal microscopy

For immunofluorescence staining, cells were washed three times with PBS and fixed with 4% paraformaldehyde (PFA) at room temperature for 30 min. Fixed cells were permeabilized with 0.1% Triton in PBS and then blocked with 5% bovine serum albumin (BSA) in PBS for 30 min. Cells were washed with 5% BSA in PBS and sequentially incubated with primary antibodies against HIV-1 p24 (Dako; Carpinteria, CA, USA) and either Rab5, −7, −11 or LAMP-1 (Santa Cruz Biotechnology) for 1 hour then washed 3 times with PBS. Secondary antibodies conjugated with Alexa594 or Alexa647 dyes (Life Technologies-Molecular Probes) were applied against the primary antibody isotype and incubated at room temperature for 1 hour then washed 3 times with PBS. Slides were covered in ProLong Gold AntiFade reagent with DAPI (Life Technologies-Molecular Probes) and imaged using a 40X oil lens on a LSM 510 confocal microscope (Carl Zeiss Microimaging, Inc.; Dublin, CA, USA) [7,67].

### Cytokine bead array

MDM were infected with HIV-1_ADA_ for 4 hours at a MOI of 0.1 then treated with 100 μM native- or nanoATV for 16 hours immediately. 24 hours after drug treatment, 50 μL cell culture media from treated and infected MDM were tested to determine the concentrations of inflammatory cytokines measured by a cytokine bead array (CBA) detection kit (Becton Dickinson Biosciences; Mississauga, ON, USA) and performed according to instructions of the manufacturer. Monoclonal antibodies specific to interleukin-12 (IL-12), tumor necrosis factor (TNF), IL-10, IL-6, IL-1β and IL-8 were added to the samples in a 96 well plate. A serial dilution of known cytokines generated the standard curve. Following three hours of incubation, all samples were acquired and analysed on a FACSArray. The standard curve was determined using a parameter logistics model and analysed with FCAP Array software. Cytokine levels are expressed as pg/mL [68].

## Additional files

Additional file 1:
**Deregulated proteins by nanoATV, nativeATV, HIV-1 infection, HIV-1 and nanoATV or HIV-1 and nativeATV treatment in MDM cells as determined by proteomics analysis.**
Additional file 2:
**Deregulated proteins during HIV-1 infection and nanoATV treatment.** Uninfected and HIV-1 infected MDM were treated with or without nanoATV. The cells were then collected for peptide identification. (A) Numbers of significantly up- or down- regulated proteins were identified by SWATH-MS and compared to uninfected and untreated MDM used as controls. (B) Changed proteins were classified according to protein class by PANTHER. These are represented by 28 independent clusters (p<0.05). Additional file 3:
**Functional characterization of significant deregulated proteins between uninfected and HIV-1-infected and nanoATV treated MDM.** Proteins were compared to uninfected/untreated control cells (p<0.05) then bioinformatics analysis was performed. The Gene Ontology molecular function (A), biological processes (B) and cellular component distribution (C) were obtained from the analysis using the Protein Analysis Through Evolutionary Relationships (PANTHER) classification system. Additional file 4:
**Annotation Clusters and Enrichment data for MDM treated with nanoATV using DAVID.**
Additional file 5:
**Annotation Clusters and Enrichment data for HIV-1 infected MDM using DAVID.**
Additional file 6:
**Annotation Clusters and Enrichment data for HIV-1 infected MDM treated with nanoATV using DAVID.**
Additional file 7:
**Protein interaction in HIV-1-infected MDM or nanoATV treated HIV-1-infected MDM.**
