# Supplementary material for: Opposing regulation of endolysosomal pathways by long-acting nanoformulated antiretroviral therapy and HIV-1 in human macrophages
Source: Retrovirology. 2015 Jan 22;12:5. doi: 10.1186/s12977-014-0133-5 (PMC4307176; doi:10.1186/s12977-014-0133-5)
Supplement: Additional file 1: — Deregulated proteins by nanoATV, nativeATV, HIV-1 infection, HIV-1 and nanoATV or HIV-1 and nativeATV treatment in MDM cells as determined by proteomics analysis. [file 12977_2014_133_MOESM1_ESM.pdf]

Additional file 1. Dysregulated proteins by HIV-1 and nanoATV in MDM cells by proteomics analysis

HIV-1

Accession	*Uniprot ID*	*Ztest*	*Ztest_pval*
P46776	RL27A_HUMAN	13.753538	0
O75367	H2AY_HUMAN	10.484407	0
P52789	HXK2_HUMAN	9.4362654	0
P61421	VA0D1_HUMAN	9.3883332	0
Q99541	PLIN2_HUMAN	8.7475457	0
O15533	TPSN_HUMAN	6.3628895	1.98E-10
P33121	ACSL1_HUMAN	6.3571503	2.055E-10
Q14764	MVP_HUMAN	6.1721913	6.735E-10
Q9BSJ8	ESYT1_HUMAN	5.9749009	2.302E-09
Q96CS3	FAF2_HUMAN	5.7788478	7.521E-09
Q14108	SCRB2_HUMAN	5.5821847	2.375E-08
Q9NZM1	MYOF_HUMAN	5.4089002	6.341E-08
P17844	DDX5_HUMAN	5.3230457	1.02E-07
P08195	4F2_HUMAN	5.2888357	1.231E-07
P38606	VATA_HUMAN	5.1201669	3.053E-07
O76021	RL1D1_HUMAN	5.0949146	3.489E-07
P23368	MAOM_HUMAN	5.0865613	3.646E-07
P33897	ABCD1_HUMAN	4.9511273	7.378E-07
P60953	CDC42_HUMAN	4.8835948	1.042E-06
P06576	ATPB_HUMAN	4.8140394	1.479E-06
P61247	RS3A_HUMAN	4.795362	1.624E-06
P10909	CLUS_HUMAN	4.7479121	2.055E-06
P30101	PDIA3_HUMAN	4.7021663	2.574E-06
P84090	ERH_HUMAN	4.6976446	2.632E-06
P30520	PURA2_HUMAN	4.6954036	2.661E-06
P62306	RUXF_HUMAN	4.6819695	2.841E-06
Q8N5M1	ATPF2_HUMAN	4.6391147	3.499E-06
O60488	ACSL4_HUMAN	4.6252384	3.742E-06
Q9NP72	RAB18_HUMAN	4.5171925	6.266E-06
Q96A33	CCD47_HUMAN	4.5010376	6.762E-06
P50914	RL14_HUMAN	4.4779332	7.537E-06
P04083	ANXA1_HUMAN	4.4027718	1.069E-05
Q15006	EMC2_HUMAN	4.3899871	1.134E-05
O15118	NPC1_HUMAN	4.3585536	1.309E-05
P11940	PABP1_HUMAN	4.3493748	1.365E-05
P05023	AT1A1_HUMAN	4.3021018	1.692E-05
P02774	VTDB_HUMAN	4.1959078	2.718E-05
Q6PIU2	NCEH1_HUMAN	4.1369446	3.52E-05
Q12906	ILF3_HUMAN	4.0969414	4.186E-05
P20339	RAB5A_HUMAN	4.0929459	4.259E-05
Q9H1C7	CYTM1_HUMAN	4.0818664	4.468E-05
P39656	OST48_HUMAN	3.9778976	6.953E-05
Q53GQ0	DHB12_HUMAN	3.9584736	7.543E-05
P20674	COX5A_HUMAN	3.8880308	0.0001011
P40616	ARL1_HUMAN	3.8651489	0.000111
P02545	LMNA_HUMAN	3.8549513	0.0001158
P55084	ECHB_HUMAN	3.8509567	0.0001177
P23246	SFPQ_HUMAN	3.7830211	0.0001549
Q9NQC3	RTN4_HUMAN	3.7655098	0.0001662
P48681	NEST_HUMAN	3.7649672	0.0001666
P50416	CPT1A_HUMAN	3.7637902	0.0001674
Q14444	CAPR1_HUMAN	3.7332876	0.000189
Q13488	VPP3_HUMAN	3.6986061	0.0002168
Q9Y285	SYFA_HUMAN	3.6694175	0.0002431
Q13636	RAB31_HUMAN	3.6452593	0.0002671
Q9P0L0	VAPA_HUMAN	3.6093409	0.000307
P05090	APOD_HUMAN	3.5625739	0.0003672
P48047	ATPO_HUMAN	3.5451833	0.0003923
P43007	SATT_HUMAN	3.5312519	0.0004136
O94776	MTA2_HUMAN	3.5202096	0.0004312
Q5JTV8	TOIP1_HUMAN	3.5156062	0.0004388
P62318	SMD3_HUMAN	3.5001948	0.0004649
P61026	RAB10_HUMAN	3.4941063	0.0004757
Q6P179	ERAP2_HUMAN	3.4923702	0.0004788
Q9UHG3	PCYOX_HUMAN	3.4673981	0.0005255
O94886	TM63A_HUMAN	3.4565256	0.0005472
P16070	CD44_HUMAN	3.4496465	0.0005613

nanoATV

Accession	*Uniprot ID*	*ZTEST*	*Ztest_pval*
Q9Y285	SYFA_HUMAN	8.0259154	0.0465289
Q14444	CAPR1_HUMAN	7.0043657	0.0206159
P60903	S10AA_HUMAN	6.4204829	0.0113791
P10909	CLUS_HUMAN	6.2562679	0.0001546
Q9NZ01	TECR_HUMAN	5.9931337	0.0432861
P13674	P4HA1_HUMAN	5.8149028	0.0004728
P61247	RS3A_HUMAN	5.6698207	0.0128499
P34810	CD68_HUMAN	5.0320344	0.005578
P18085	ARF4_HUMAN	4.2236686	0.0009589
P39019	RS19_HUMAN	4.0126993	0.0063925
Q96HY6	DDRGK_HUMAN	3.9248461	0.0311589
Q03169	TNAP2_HUMAN	3.8927064	0.0155109
P16671	CD36_HUMAN	3.5933459	0.0008097
O75489	NDUS3_HUMAN	3.5882714	2.058E-09
P61163	ACTZ_HUMAN	3.4490283	0.0117187
P08195	4F2_HUMAN	3.4353843	9.824E-05
P28838	AMPL_HUMAN	3.3796618	0.0041004
P05120	PAI2_HUMAN	3.379526	5.407E-05
P33121	ACSL1_HUMAN	3.3638484	0.004977
O95721	SNP29_HUMAN	3.349471	1.429E-08
Q9NP72	RAB18_HUMAN	3.2260045	0.0415881
Q9Y613	FHOD1_HUMAN	3.2185897	0.0492723
Q7Z3B4	NUP54_HUMAN	3.1997223	0.0269084
Q01459	DIAC_HUMAN	3.1926651	0.0150993
Q8NF37	PCAT1_HUMAN	3.1604147	0.0286764
P48637	GSHB_HUMAN	3.1062768	0.0085222
Q14956	GPNMB_HUMAN	3.0913825	0.021046
P01876	IGHA1_HUMAN	3.0333155	2.332E-05
P48681	NEST_HUMAN	3.0080141	0.00864
O43567	RNF13_HUMAN	2.8709562	4.814E-11
P20339	RAB5A_HUMAN	2.8703356	0.0013629
P01011	AACT_HUMAN	2.8566873	1.48E-05
P00558	PGK1_HUMAN	2.8247254	4.853E-07
P30101	PDIA3_HUMAN	2.7385054	0.0047021
P05090	APOD_HUMAN	2.7269365	5.326E-05
Q9UMS4	PRP19_HUMAN	2.7224037	0.0449012
P49841	GSK3B_HUMAN	2.7162723	0.0091117
P20701	ITAL_HUMAN	2.6778547	0.0014097
P01024	CO3_HUMAN	2.6680592	1.766E-05
Q13510	ASAH1_HUMAN	2.6306497	0.0187494
Q6NUT3	MFS12_HUMAN	2.6218866	0.0247228
P42765	THIM_HUMAN	2.605222	0.0076291
Q06210	GFPT1_HUMAN	2.5853928	0.0156402
P10599	THIO_HUMAN	2.5791799	0.0001338
P26599	PTBP1_HUMAN	2.5588158	0.0029234
Q86U42	PABP2_HUMAN	2.5553191	0.0270893
Q86VS8	HOOK3_HUMAN	2.4879015	0.0271544
Q92688	AN32B_HUMAN	2.4599574	0.0305193
Q969H8	CS010_HUMAN	2.4172049	0.0308167
Q9Y3E5	PTH2_HUMAN	2.384567	0.0470077
P01777	HV316_HUMAN	2.3824175	2.209E-05
P55036	PSMD4_HUMAN	2.3718496	0.010609
O15229	KMO_HUMAN	2.3071576	2.481E-12
P53674	CRBB1_HUMAN	2.3045159	0.0105029
Q9H7F0	AT133_HUMAN	2.2197842	0.0394348
Q14974	IMB1_HUMAN	2.2059882	0.0211937
P46976	GLYG_HUMAN	2.1965703	0.0078383
P50416	CPT1A_HUMAN	2.190335	0.0097268
P62995	TRA2B_HUMAN	2.1632847	0.0138953
Q562E7	WDR81_HUMAN	2.1607144	0.0221679
P10321	1C07_HUMAN	2.1550375	0.0001257
P51665	PSMD7_HUMAN	2.141487	0.010005
P20073	ANXA7_HUMAN	2.1209299	0.0012882
P20674	COX5A_HUMAN	2.1200319	0.0013756
P22061	PIMT_HUMAN	2.1073329	0.0016602
Q9Y5L4	TIM13_HUMAN	2.084555	0.0477498
P21281	VATB2_HUMAN	2.0746006	0.001441

HIV-1 + nanoATV

Accession	*Uniprot ID*	*Ztest*	*Ztest_pval*
P04080	CYTB_HUMAN	11.848533	0
P21980	TGM2_HUMAN	9.7920862	0
P22307	NLTP_HUMAN	9.1141676	0
O75368	SH3L1_HUMAN	8.4573785	0
Q13162	PRDX4_HUMAN	7.6183519	2.576E-14
Q9BRF8	CPPED_HUMAN	7.559198	4.063E-14
Q16555	DPYL2_HUMAN	7.1279387	1.019E-12
Q9Y4W6	AFG32_HUMAN	6.7389982	1.595E-11
P49768	PSN1_HUMAN	6.2231097	4.874E-10
Q9Y6W5	WASF2_HUMAN	5.7428713	9.308E-09
Q9BZE4	NOG1_HUMAN	5.6302214	1.8E-08
P51991	ROA3_HUMAN	5.5238822	3.316E-08
Q8N3D4	EH1L1_HUMAN	5.2558644	1.473E-07
Q9NRV9	HEBP1_HUMAN	5.2062667	1.927E-07
P49721	PSB2_HUMAN	5.1275001	2.936E-07
P35914	HMGCL_HUMAN	5.1090569	3.238E-07
P50440	GATM_HUMAN	5.0968049	3.454E-07
P04233	HG2A_HUMAN	5.0540879	4.325E-07
P26006	ITA3_HUMAN	5.0441241	4.556E-07
Q9Y2B0	CNPY2_HUMAN	5.0260559	5.007E-07
P12955	PEPD_HUMAN	4.9163969	8.815E-07
P60981	DEST_HUMAN	4.8965997	9.751E-07
Q8IZ83	A16A1_HUMAN	4.8505924	1.231E-06
P30084	ECHM_HUMAN	4.7861445	1.7E-06
P68371	TBB4B_HUMAN	4.7667771	1.872E-06
Q96T60	PNKP_HUMAN	4.7632807	1.905E-06
Q9H3G5	CPVL_HUMAN	4.7606348	1.93E-06
O60664	PLIN3_HUMAN	4.68021	2.866E-06
O95202	LETM1_HUMAN	4.5503431	5.356E-06
P23528	COF1_HUMAN	4.548955	5.391E-06
P27816	MAP4_HUMAN	4.5465537	5.453E-06
Q12931	TRAP1_HUMAN	4.4152889	1.009E-05
O43399	TPD54_HUMAN	4.3671363	1.259E-05
P11177	ODPB_HUMAN	4.3557722	1.326E-05
P14550	AK1A1_HUMAN	4.3487165	1.369E-05
P30405	PPIF_HUMAN	4.3097267	1.635E-05
P30086	PEBP1_HUMAN	4.298459	1.72E-05
Q9Y333	LSM2_HUMAN	4.2745371	1.915E-05
Q8TC12	RDH11_HUMAN	4.2697833	1.957E-05
P46926	GNPI1_HUMAN	4.2473416	2.163E-05
P23526	SAHH_HUMAN	4.2238165	2.402E-05
P50552	VASP_HUMAN	4.2144597	2.504E-05
P30038	AL4A1_HUMAN	4.2113317	2.539E-05
Q9BUF5	TBB6_HUMAN	4.1439202	3.414E-05
Q15691	MARE1_HUMAN	4.0631976	4.841E-05
Q9NZM1	MYOF_HUMAN	4.0282332	5.62E-05
P62937	PPIA_HUMAN	4.0043857	6.218E-05
P08648	ITA5_HUMAN	3.9632746	7.393E-05
P14866	HNRPL_HUMAN	3.9310535	8.457E-05
Q9NS69	TOM22_HUMAN	3.9279602	8.567E-05
Q9H223	EHD4_HUMAN	3.9266699	8.613E-05
P22087	FBRL_HUMAN	3.8950026	9.82E-05
Q99497	PARK7_HUMAN	3.88827	0.000101
P29373	RABP2_HUMAN	3.861803	0.0001126
P25685	DNJB1_HUMAN	3.8575794	0.0001145
Q09028	RBBP4_HUMAN	3.8555929	0.0001154
P06733	ENOA_HUMAN	3.7990414	0.0001453
O43759	SNG1_HUMAN	3.717633	0.0002011
P82650	RT22_HUMAN	3.7086105	0.0002084
P54725	RD23A_HUMAN	3.7023883	0.0002136
P35606	COPB2_HUMAN	3.6936984	0.000221
Q68CZ2	TENS3_HUMAN	3.6562305	0.000256
P13686	PPA5_HUMAN	3.6273236	0.0002864
O15511	ARPC5_HUMAN	3.5990954	0.0003193
P13798	ACPH_HUMAN	3.5906656	0.0003298
Q96FZ7	CHMP6_HUMAN	3.5424128	0.0003965
P63220	RS21_HUMAN	3.5414725	0.0003979

Q03518	TAP1_HUMAN	3.3788603	0.0007279
P10412	H14_HUMAN	3.358679	0.0007832
O95881	TXD12_HUMAN	3.3211372	0.0008965
P20591	MX1_HUMAN	3.3061752	0.0009458
P63244	GBLP_HUMAN	3.2763508	0.0010516
Q8WW59	SPRY4_HUMAN	3.2735843	0.0010619
Q96SQ9	CP2S1_HUMAN	3.2701208	0.001075
Q12907	LMAN2_HUMAN	3.2588169	0.0011188
P67809	YBOX1_HUMAN	3.236587	0.0012097
P42224	STAT1_HUMAN	3.2239321	0.0012644
Q9HDC9	APMAP_HUMAN	3.2187852	0.0012873
Q06136	KDSR_HUMAN	3.2173564	0.0012938
Q9Y5M8	SRPRB_HUMAN	3.1746164	0.0015003
Q13217	DNJC3_HUMAN	3.1720405	0.0015137
Q92841	DDX17_HUMAN	3.1060171	0.0018963
Q9Y4K1	AIM1_HUMAN	3.1042995	0.0019073
P01857	IGHG1_HUMAN	3.0848916	0.0020363
P10606	COX5B_HUMAN	3.0823628	0.0020536
P49411	EFTU_HUMAN	3.0821616	0.0020549
P21757	MSRE_HUMAN	3.0784892	0.0020805
Q9H3N1	TMX1_HUMAN	3.0472785	0.0023092
Q02978	M2OM_HUMAN	3.0431524	0.0023411
Q01844	EWS_HUMAN	3.0333451	0.0024186
P51571	SSRD_HUMAN	3.0127044	0.0025893
P01876	IGHA1_HUMAN	2.9941066	0.0027525
Q2M389	WASH7_HUMAN	2.9685423	0.0029922
O95721	SNP29_HUMAN	2.9610441	0.003066
P04062	GLCM_HUMAN	2.9577299	0.0030991
Q96D96	HVCN1_HUMAN	2.9474645	0.0032039
P40926	MDHM_HUMAN	2.9447027	0.0032327
Q96HE7	ERO1A_HUMAN	2.9404174	0.0032777
Q9NZ01	TECR_HUMAN	2.9391627	0.003291
P01779	HV318_HUMAN	2.9101175	0.0036129
Q99613	EIF3C_HUMAN	2.9034603	0.0036906
P11279	LAMP1_HUMAN	2.8775611	0.0040076
Q9NTJ5	SAC1_HUMAN	2.8741325	0.0040514
P21964	COMT_HUMAN	2.8733039	0.004062
P20340	RAB6A_HUMAN	2.8728303	0.0040681
O94925	GLSK_HUMAN	2.8688506	0.0041197
P00387	NB5R3_HUMAN	2.8589059	0.004251
P42765	THIM_HUMAN	2.8318567	0.0046279
Q12846	STX4_HUMAN	2.8314093	0.0046343
P62701	RS4X_HUMAN	2.8182716	0.0048283
Q86UE4	LYRIC_HUMAN	2.7958062	0.005177
Q9UJZ1	STML2_HUMAN	2.7885363	0.0052947
P21796	VDAC1_HUMAN	2.7451367	0.0060486
Q9Y6C9	MTCH2_HUMAN	2.7203913	0.0065205
P04004	VTNC_HUMAN	2.7152648	0.0066223
Q96AH8	RAB7B_HUMAN	2.7142191	0.0066432
P01871	IGHM_HUMAN	2.7025293	0.0068814
P68431	H31_HUMAN	2.6994707	0.006945
P05141	ADT2_HUMAN	2.6597826	0.0078191
Q9BRQ8	AIFM2_HUMAN	2.6514405	0.0080149
P10301	RRAS_HUMAN	2.6457498	0.008151
P18136	KV313_HUMAN	2.6434934	0.0082055
P02786	TFR1_HUMAN	2.6215132	0.008754
Q99685	MGLL_HUMAN	2.6113671	0.0090181
Q9NYU2	UGGG1_HUMAN	2.584411	0.0097545
O75477	ERLN1_HUMAN	2.5775221	0.0099512
Q06210	GFPT1_HUMAN	2.5706013	0.0101522
P20073	ANXA7_HUMAN	2.570333	0.0101601
P01777	HV316_HUMAN	2.5597579	0.0104745
Q9BTZ2	DHRS4_HUMAN	2.5515359	0.0107249
P47914	RL29_HUMAN	2.5472495	0.0108576
P12236	ADT3_HUMAN	2.541526	0.011037
Q92552	RT27_HUMAN	2.539909	0.0110881
P07910	HNRPC_HUMAN	2.5207472	0.0117106
Q8IY17	PLPL6_HUMAN	2.5202435	0.0117274
Q15084	PDIA6_HUMAN	2.5122817	0.0119953
P02787	TRFE_HUMAN	2.5000966	0.0124159
P13804	ETFA_HUMAN	2.4939782	0.012632
P23396	RS3_HUMAN	2.4904181	0.0127593
P60033	CD81_HUMAN	2.0667073	0.0108147
P00738	HPT_HUMAN	2.0596208	8.212E-07
O14744	ANM5_HUMAN	2.0453075	0
P07602	SAP_HUMAN	2.0281924	6.003E-05
O14874	BCKD_HUMAN	2.0123445	1.11E-15
P04179	SODM_HUMAN	2.0115264	4.015E-05
Q9BWD1	THIC_HUMAN	1.9905579	0.0371097
Q9UKK9	NUDT5_HUMAN	1.9862312	0.0441836
Q5VTR2	BRE1A_HUMAN	1.9795887	0.0245469
Q99714	HCD2_HUMAN	1.9659117	0.0340034
P25786	PSA1_HUMAN	-1.966228	0.0026621
P35268	RL22_HUMAN	-1.9783914	0.0056458
P40227	TCPZ_HUMAN	-1.9840104	0.0064809
Q9ULV4	COR1C_HUMAN	-1.9905638	0.0446807
P50440	GATM_HUMAN	-2.0055788	0.0099035
P23528	COF1_HUMAN	-2.0076482	0.002101
Q9BR76	COR1B_HUMAN	-2.0131211	0.035406
Q9H0U4	RAB1B_HUMAN	-2.0195801	0.0380236
Q93050	VPP1_HUMAN	-2.0209387	0.0307174
P11233	RALA_HUMAN	-2.0376183	0.000197
Q96RP9	EFGM_HUMAN	-2.0694395	0.0312414
P18859	ATP5J_HUMAN	-2.0714786	0.0010086
P00918	CAH2_HUMAN	-2.0858573	9.676E-06
O14828	SCAM3_HUMAN	-2.0940391	1.11E-15
P40926	MDHM_HUMAN	-2.1036838	0.0069561
Q8NBS9	TXND5_HUMAN	-2.1539838	0.0285
P20042	IF2B_HUMAN	-2.1594313	0.0012553
P19105	ML12A_HUMAN	-2.1637648	0.0011801
Q9UHA4	LTOR3_HUMAN	-2.1879073	0.0441019
Q9H4A4	AMPB_HUMAN	-2.2092908	0.0385049
P61160	ARP2_HUMAN	-2.2102283	0.0114881
O96011	PX11B_HUMAN	-2.2128433	1.576E-07
Q08379	GOGA2_HUMAN	-2.2457061	0.0170992
P61221	ABCE1_HUMAN	-2.2484599	0.012611
P78527	PRKDC_HUMAN	-2.2874785	0.014603
P31146	COR1A_HUMAN	-2.2894403	0.0047321
Q99439	CNN2_HUMAN	-2.2974857	0.0322348
P30040	ERP29_HUMAN	-2.3149457	0.0042809
O75436	VP26A_HUMAN	-2.3221945	3.943E-10
P22307	NLTP_HUMAN	-2.3504768	0.0018946
P50552	VASP_HUMAN	-2.3718746	0.0099891
Q9Y286	SIGL7_HUMAN	-2.3906421	0.0465295
P11310	ACADM_HUMAN	-2.4150194	0.0002209
P09874	PARP1_HUMAN	-2.4202253	9.913E-05
Q99541	PLIN2_HUMAN	-2.4299892	0.0339277
Q86Y82	STX12_HUMAN	-2.4309719	0.0273848
Q9HC35	EMAL4_HUMAN	-2.4420771	0.0408246
P35998	PRS7_HUMAN	-2.4421987	0.0059543
Q13162	PRDX4_HUMAN	-2.4669482	0.0176993
Q9Y4W6	AFG32_HUMAN	-2.4848621	0.0472547
Q16836	HCDH_HUMAN	-2.494571	0.0220538
P04080	CYTB_HUMAN	-2.520504	2.404E-05
P55957	BID_HUMAN	-2.5274914	0.0107873
Q9Y5P6	GMPPB_HUMAN	-2.5308363	0.0478846
P62937	PPIA_HUMAN	-2.5331076	0.0129602
P50213	IDH3A_HUMAN	-2.5486289	0.0091815
Q8WUM4	PDC6I_HUMAN	-2.5495146	0.0304824
P60842	IF4A1_HUMAN	-2.5498261	0.0113056
Q9BVG4	PBDC1_HUMAN	-2.5756553	0.0369916
P15090	FABP4_HUMAN	-2.576208	0.0005626
P26640	SYVC_HUMAN	-2.5872268	0.003222
P33241	LSP1_HUMAN	-2.6078354	0.0051627
P22059	OSBP1_HUMAN	-2.6162573	0.0016254
A6NL28	TPM3L_HUMAN	-2.6259806	0
P49755	TMEDA_HUMAN	-2.653644	0.00889
P00813	ADA_HUMAN	-2.658956	7.107E-06
P13489	RINI_HUMAN	-2.6640738	0.0004273
P23526	SAHH_HUMAN	-2.6989397	0.0019923
Q9Y679	AUP1_HUMAN	-2.7372397	0.0493088
P48047	ATPO_HUMAN	-2.7502871	0.0079628
P08107	HSP71_HUMAN	-2.7546619	8.679E-05
Q06830	PRDX1_HUMAN	-2.7676762	0.0157344
Q15942	ZYX_HUMAN	3.5131921	0.0004428
P11216	PYGB_HUMAN	3.5062331	0.0004545
Q86Y82	STX12_HUMAN	3.4690544	0.0005223
P31150	GDIA_HUMAN	3.3924425	0.0006927
O00483	NDUA4_HUMAN	3.3906463	0.0006973
P17987	TCPA_HUMAN	3.364011	0.0007682
P50897	PPT1_HUMAN	3.3225367	0.000892
O75436	VP26A_HUMAN	3.3165883	0.0009112
O75369	FLNB_HUMAN	3.316552	0.0009114
O00233	PSMD9_HUMAN	3.2985134	0.000972
P62258	1433E_HUMAN	3.2905051	0.0010001
Q14165	MLEC_HUMAN	3.2872283	0.0010118
Q6ZUJ8	BCAP_HUMAN	3.26844	0.0010814
Q13596	SNX1_HUMAN	3.260177	0.0011134
O43169	CYB5B_HUMAN	3.2585587	0.0011198
P15311	EZRI_HUMAN	3.2134343	0.0013116
O75390	CISY_HUMAN	3.2036241	0.0013571
P01034	CYTC_HUMAN	3.1944637	0.0014009
Q8NC56	LEMD2_HUMAN	3.1594365	0.0015807
Q06830	PRDX1_HUMAN	3.0762069	0.0020965
P25098	ARBK1_HUMAN	3.0649413	0.0021771
P62873	GBB1_HUMAN	3.0576515	0.0022308
O60313	OPA1_HUMAN	3.0468849	0.0023123
P50990	TCPQ_HUMAN	3.0331446	0.0024202
P28072	PSB6_HUMAN	3.0205092	0.0025235
P24752	THIL_HUMAN	3.0043384	0.0026616
Q9Y3I0	RTCB_HUMAN	2.9813132	0.0028702
P21333	FLNA_HUMAN	2.935005	0.0033354
O95336	6PGL_HUMAN	2.9323611	0.003364
Q9Y2Q3	GSTK1_HUMAN	2.9203935	0.0034959
Q6DD88	ATLA3_HUMAN	2.9059214	0.0036617
P41091	IF2G_HUMAN	2.8666415	0.0041485
Q96JJ3	ELMO2_HUMAN	2.850604	0.0043636
P55957	BID_HUMAN	2.8404207	0.0045054
O43175	SERA_HUMAN	2.8287504	0.004673
P08754	GNAI3_HUMAN	2.8212285	0.004784
Q8TCJ2	STT3B_HUMAN	2.8136801	0.0048978
P07437	TBB5_HUMAN	2.8061545	0.0050137
O75431	MTX2_HUMAN	2.7976175	0.0051481
P49588	SYAC_HUMAN	2.7967465	0.005162
Q9Y3B3	TMED7_HUMAN	2.7857486	0.0053404
Q8TAT6	NPL4_HUMAN	2.7503252	0.0059536
P01040	CYTA_HUMAN	2.7386229	0.0061697
P48643	TCPE_HUMAN	2.7329026	0.0062779
P11233	RALA_HUMAN	2.7281435	0.0063692
Q9NQ88	TIGAR_HUMAN	2.7234371	0.0064607
P33241	LSP1_HUMAN	2.7061606	0.0068066
P16152	CBR1_HUMAN	2.7035247	0.0068608
Q14103	HNRPD_HUMAN	2.6988781	0.0069574
Q6P179	ERAP2_HUMAN	2.698345	0.0069685
P62987	RL40_HUMAN	2.6980627	0.0069744
Q04837	SSBP_HUMAN	2.6974715	0.0069868
P53597	SUCA_HUMAN	2.6833675	0.0072885
P15090	FABP4_HUMAN	2.6795542	0.007372
P30519	HMOX2_HUMAN	2.6763248	0.0074434
Q15836	VAMP3_HUMAN	2.6702041	0.0075805
P34910	EVI2B_HUMAN	2.6624393	0.0077577
Q15427	SF3B4_HUMAN	2.6567809	0.0078891
P07900	HS90A_HUMAN	2.6536535	0.0079626
Q9H1C7	CYTM1_HUMAN	2.6348326	0.0084179
P52565	GDIR1_HUMAN	2.6240264	0.0086897
P61160	ARP2_HUMAN	2.616083	0.0088945
O96005	CLPT1_HUMAN	2.5937601	0.0094933
Q9ULA0	DNPEP_HUMAN	2.5867191	0.0096895
Q8NF50	DOCK8_HUMAN	2.5571314	0.0105539
Q96A26	F162A_HUMAN	2.5344362	0.0112628
P28066	PSA5_HUMAN	2.5312703	0.011365
Q9UJ70	NAGK_HUMAN	2.4951561	0.0125902
P56537	IF6_HUMAN	2.4904146	0.0127594
Q7Z4G1	COMD6_HUMAN	2.4867572	0.0128913
Q9Y230	RUVB2_HUMAN	2.4800621	0.013136
P50395	GDIB_HUMAN	2.465016	0.0137007

Q14247	SRC8_HUMAN	2.4854933	0.0129372
P36776	LONM_HUMAN	2.4826545	0.0130408
Q9UBV2	SE1L1_HUMAN	2.4804533	0.0131215
O75352	MPU1_HUMAN	2.4795316	0.0131555
P40939	ECHA_HUMAN	2.4729303	0.013401
Q9P2E9	RRBP1_HUMAN	2.4723227	0.0134238
Q00765	REEP5_HUMAN	2.4614905	0.0138361
O15270	SPTC2_HUMAN	2.4613036	0.0138433
Q5TDH0	DDI2_HUMAN	2.4547966	0.0140964
P49756	RBM25_HUMAN	2.4436272	0.0145404
P10515	ODP2_HUMAN	2.4394932	0.0147079
P35527	K1C9_HUMAN	2.4316214	0.0150314
P08236	BGLR_HUMAN	2.4301975	0.0150906
P61313	RL15_HUMAN	2.4084798	0.0160191
Q6IAA8	LTOR1_HUMAN	2.4083719	0.0160238
P29692	EF1D_HUMAN	2.4037208	0.0162292
P01834	IGKC_HUMAN	2.3921239	0.0167512
P06748	NPM_HUMAN	2.3881474	0.0169335
Q13283	G3BP1_HUMAN	2.3871819	0.0169781
Q15020	SART3_HUMAN	2.3763198	0.0174863
P02751	FINC_HUMAN	2.3648113	0.0180393
Q8NF37	PCAT1_HUMAN	2.3644195	0.0180583
Q9NZ08	ERAP1_HUMAN	2.3388507	0.0193432
P10109	ADX_HUMAN	2.3386224	0.019355
P09525	ANXA4_HUMAN	2.3366836	0.0194556
O75410	TACC1_HUMAN	2.3360918	0.0194865
P02656	APOC3_HUMAN	2.3339173	0.0196001
Q13724	MOGS_HUMAN	2.3216209	0.0202534
P34810	CD68_HUMAN	2.3212163	0.0202752
Q96HY6	DDRGK_HUMAN	2.3155123	0.0205849
P51572	BAP31_HUMAN	2.3098962	0.0208939
P78527	PRKDC_HUMAN	2.3054227	0.0211429
Q9BTU6	P4K2A_HUMAN	2.2902193	0.0220086
P0CG06	LAC3_HUMAN	2.2797252	0.022624
O75643	U520_HUMAN	2.2422903	0.0249426
Q8N0X4	CLYBL_HUMAN	2.2370851	0.0252808
P04844	RPN2_HUMAN	2.2298858	0.025755
P31040	DHSA_HUMAN	2.2040609	0.0275201
P08865	RSSA_HUMAN	2.1998784	0.0278155
P62241	RS8_HUMAN	2.1856805	0.028839
Q9NWV8	BABA1_HUMAN	2.180191	0.0292433
P26599	PTBP1_HUMAN	2.1723981	0.0298256
O14744	ANM5_HUMAN	2.1669165	0.0302412
Q27J81	INF2_HUMAN	2.1668426	0.0302469
P46782	RS5_HUMAN	2.1642066	0.0304485
O14979	HNRDL_HUMAN	2.1484682	0.0316766
P68032	ACTC_HUMAN	2.1404891	0.0323153
Q07955	SRSF1_HUMAN	2.1398821	0.0323643
O75390	CISY_HUMAN	2.1309807	0.0330907
P46783	RS10_HUMAN	2.1298044	0.0331878
Q9Y6G9	DC1L1_HUMAN	2.1255227	0.033543
P18031	PTN1_HUMAN	2.1240797	0.0336635
Q9NR28	DBLOH_HUMAN	2.1223935	0.0338047
P16435	NCPR_HUMAN	2.1151543	0.0344168
P17931	LEG3_HUMAN	2.1042716	0.0353548
P25705	ATPA_HUMAN	2.0845342	0.0371116
P61604	CH10_HUMAN	2.0699236	0.0384595
P09651	ROA1_HUMAN	2.0664111	0.0387897
O43760	SNG2_HUMAN	2.066019	0.0388267
P30044	PRDX5_HUMAN	2.0512328	0.0402443
Q99623	PHB2_HUMAN	2.047229	0.0406356
P02768	ALBU_HUMAN	2.044573	0.040897
Q15907	RB11B_HUMAN	2.0438419	0.0409692
P05388	RLA0_HUMAN	2.0347153	0.0418795
Q9Y6A9	SPCS1_HUMAN	2.0282533	0.0425344
O00186	STXB3_HUMAN	2.019777	0.0434065
O15127	SCAM2_HUMAN	2.0115799	0.0442642
P36957	ODO2_HUMAN	2.0078971	0.0446542
P08962	CD63_HUMAN	2.0040169	0.0450682
P53674	CRBB1_HUMAN	2.0015066	0.0453378
Q8NBQ5	DHB11_HUMAN	1.9966857	0.0458593
P51148	RAB5C_HUMAN	1.9888709	0.0467155

Q7L576	CYFP1_HUMAN	-2.7716087	0.0264334
P01040	CYTA_HUMAN	-2.7967037	1.825E-05
P16152	CBR1_HUMAN	-2.8085161	0.0007261
O00757	F16P2_HUMAN	-2.8202814	0
O15212	PFD6_HUMAN	-2.8212549	1.554E-15
O43169	CYB5B_HUMAN	-2.8267636	6.601E-12
Q9NRV9	HEBP1_HUMAN	-2.9143604	0.0425406
P07737	PROF1_HUMAN	-2.9457262	6.949E-05
Q09161	NCBP1_HUMAN	-2.9756775	0.0171994
P15153	RAC2_HUMAN	-2.9883303	0.0005917
P27105	STOM_HUMAN	-3.0042785	0.0035642
P38646	GRP75_HUMAN	-3.0475791	0.0061957
P55795	HNRH2_HUMAN	-3.0755735	0.0107777
P26006	ITA3_HUMAN	-3.1041243	0.0028051
P15311	EZRI_HUMAN	-3.1451102	0.0007258
O60443	DFNA5_HUMAN	-3.1513057	1.358E-10
P45877	PPIC_HUMAN	-3.1863052	0.0077201
P21980	TGM2_HUMAN	-3.2023887	0.0015754
Q9ULA0	DNPEP_HUMAN	-3.2436581	0.0442699
P13073	COX41_HUMAN	-3.2881292	0.0003329
Q9H1C7	CYTM1_HUMAN	-3.3023056	0.0383141
P62942	FKB1A_HUMAN	-3.3069754	0.013627
P09960	LKHA4_HUMAN	-3.4957378	0.0001131
P48735	IDHP_HUMAN	-3.5226005	0.0087445
P17980	PRS6A_HUMAN	-3.665059	0.0009431
Q14980	NUMA1_HUMAN	-3.6938415	0.0215911
P68032	ACTC_HUMAN	-3.7228617	0.0145981
P27797	CALR_HUMAN	-3.7337381	0.0040923
P31930	QCR1_HUMAN	-3.7835268	0.0047982
P11216	PYGB_HUMAN	-3.8160104	0.0001887
P04632	CPNS1_HUMAN	-3.8194368	4.043E-05
Q99873	ANM1_HUMAN	-3.8347243	0.0350887
P12236	ADT3_HUMAN	-3.8606436	0.0002473
Q9H4E7	DEFI6_HUMAN	-3.8949064	0.0387617
O75368	SH3L1_HUMAN	-3.9780378	2.047E-10
Q9NZM1	MYOF_HUMAN	-4.0372902	0.043427
P36222	CH3L1_HUMAN	-4.0408334	0.0061719
P24752	THIL_HUMAN	-4.1050001	0.0023069
Q8IZ83	A16A1_HUMAN	-4.1066024	0.0280511
P25398	RS12_HUMAN	-4.2305215	0.0024188
P35914	HMGCL_HUMAN	-4.2425976	0.0058753
P82650	RT22_HUMAN	-4.2853004	0.0150584
P16402	H13_HUMAN	-4.2925931	0.0007686
O94874	UFL1_HUMAN	-4.3316792	6.067E-09
P41091	IF2G_HUMAN	-4.4242898	0.0074095
P50395	GDIB_HUMAN	-4.4904602	0.0096752
Q12931	TRAP1_HUMAN	-4.930254	0.0176981
P12955	PEPD_HUMAN	-5.243418	0.0003265
P25705	ATPA_HUMAN	-6.3578012	0.0026296
Q13596	SNX1_HUMAN	-6.5765746	0.0202225
P22314	UBA1_HUMAN	-6.8660371	0.0019084
P31150	GDIA_HUMAN	-7.9672287	0.0047836
Q07955	SRSF1_HUMAN	-8.0205919	0.0168189
Q9BVC6	TM109_HUMAN	-8.6103647	0.0362565
P40925	MDHC_HUMAN	-10.954493	0.0066022
P10809	CH60_HUMAN	-12.935813	0.0001356

Q9HB71	CYBP_HUMAN	2.4641516	0.0137338
P58546	MTPN_HUMAN	2.4500368	0.0142842
O00754	MA2B1_HUMAN	2.4450599	0.0144828
P00338	LDHA_HUMAN	2.4441052	0.0145212
P29350	PTN6_HUMAN	2.4369762	0.0148107
Q9NX40	OCAD1_HUMAN	2.4208502	0.0154843
O95865	DDAH2_HUMAN	2.4184513	0.0155867
O00161	SNP23_HUMAN	2.4167275	0.0156607
Q99439	CNN2_HUMAN	2.4094095	0.0159784
Q9H269	VPS16_HUMAN	2.405501	0.0161503
Q9H4E7	DEFI6_HUMAN	2.3933378	0.0166959
P45880	VDAC2_HUMAN	2.3602602	0.0182621
P49327	FAS_HUMAN	2.3583796	0.0183549
P54578	UBP14_HUMAN	2.355313	0.0185071
P22695	QCR2_HUMAN	2.3516717	0.0186893
O94903	PROSC_HUMAN	2.3434986	0.0191038
Q8IYB3	SRRM1_HUMAN	2.3254614	0.0200473
Q9BVC6	TM109_HUMAN	2.3254295	0.020049
O43396	TXNL1_HUMAN	2.3170812	0.0204993
Q93050	VPP1_HUMAN	2.3160172	0.0205573
P00813	ADA_HUMAN	2.3123221	0.0207599
P48735	IDHP_HUMAN	2.308395	0.0209772
Q9BZG1	RAB34_HUMAN	2.3062403	0.0210972
P62942	FKB1A_HUMAN	2.2879386	0.0221411
P53634	CATC_HUMAN	2.2870368	0.0221937
Q9BRK5	CAB45_HUMAN	2.2778843	0.0227335
Q15393	SF3B3_HUMAN	2.2777411	0.022742
Q8TD55	PKHO2_HUMAN	2.2615875	0.0237229
O43747	AP1G1_HUMAN	2.2544367	0.0241687
P50148	GNAQ_HUMAN	2.2533976	0.0242341
P50213	IDH3A_HUMAN	2.2420938	0.0249553
P22314	UBA1_HUMAN	2.2415378	0.0249913
Q96GA7	SDSL_HUMAN	2.2252914	0.0260617
P62993	GRB2_HUMAN	2.222609	0.0262422
P10809	CH60_HUMAN	2.2210746	0.0263459
Q6IBS0	TWF2_HUMAN	2.2060634	0.0273796
Q0VD83	APOBR_HUMAN	2.1973729	0.0279938
P50452	SPB8_HUMAN	2.1896287	0.0285512
P55072	TERA_HUMAN	2.1696619	0.0300325
Q9HC38	GLOD4_HUMAN	2.1612037	0.0306796
Q9NY15	STAB1_HUMAN	2.1543422	0.0312133
Q9H4A4	AMPB_HUMAN	2.1524928	0.0313586
O75439	MPPB_HUMAN	2.1479873	0.0317148
P55265	DSRAD_HUMAN	2.1435899	0.0320658
P16402	H13_HUMAN	2.1401421	0.0323433
Q9BXD5	NPL_HUMAN	2.140106	0.0323462
P16333	NCK1_HUMAN	2.1394848	0.0323964
Q9UMX5	NENF_HUMAN	2.1382514	0.0324963
Q13576	IQGA2_HUMAN	2.1343044	0.0328179
P25788	PSA3_HUMAN	2.1280368	0.033334
P26572	MGAT1_HUMAN	2.1233197	0.0337271
P00352	AL1A1_HUMAN	2.1196148	0.0340385
P40925	MDHC_HUMAN	2.1119357	0.034692
P35998	PRS7_HUMAN	2.1118868	0.0346962
Q9Y5P6	GMPPB_HUMAN	2.1075278	0.0350719
Q9HC35	EMAL4_HUMAN	2.1034669	0.035425
P00390	GSHR_HUMAN	2.0974431	0.0359544
Q05519	SRS11_HUMAN	2.0910221	0.0365261
P01920	DQB1_HUMAN	2.0848968	0.0370787
P14314	GLU2B_HUMAN	2.0848671	0.0370814
P07237	PDIA1_HUMAN	2.0796561	0.0375571
P46459	NSF_HUMAN	2.0773753	0.0377669
P43034	LIS1_HUMAN	2.0756163	0.0379294
Q16543	CDC37_HUMAN	2.063443	0.0390706
Q9UEU0	VTI1B_HUMAN	2.0614057	0.0392643
P62328	TYB4_HUMAN	2.061367	0.039268
Q15843	NEDD8_HUMAN	2.0551613	0.0398634
Q10567	AP1B1_HUMAN	2.0473523	0.0406235
P53602	MVD1_HUMAN	2.0456094	0.0407948
Q9H2H8	PPIL3_HUMAN	2.0320461	0.042149
Q9NZB2	F120A_HUMAN	2.0306806	0.0422874
P27797	CALR_HUMAN	2.0274702	0.0426144

Q9H7F0	AT133_HUMAN	1.9838415	0.0472735
Q9H3K2	GHITM_HUMAN	1.9777445	0.0479575
O60506	HNRPQ_HUMAN	1.9726003	0.0485411
O75396	SC22B_HUMAN	1.9691376	0.0489373
Q53EU6	GPAT3_HUMAN	1.9669726	0.0491864
P34932	HSP74_HUMAN	-1.9681003	0.0490565
P29144	TPP2_HUMAN	-1.9727451	0.0485246
P61221	ABCE1_HUMAN	-1.9792124	0.0477921
Q9UHX1	PUF60_HUMAN	-1.9899075	0.0466011
P25788	PSA3_HUMAN	-2.0043054	0.0450374
Q14204	DYHC1_HUMAN	-2.0074707	0.0446996
Q7Z3B4	NUP54_HUMAN	-2.0077992	0.0446646
P04632	CPNS1_HUMAN	-2.0109268	0.0443332
Q96FZ7	CHMP6_HUMAN	-2.0188976	0.0434979
P20039	2B1B_HUMAN	-2.024358	0.0429333
P53396	ACLY_HUMAN	-2.0409774	0.0412531
P55160	NCKPL_HUMAN	-2.0479716	0.0405628
P31939	PUR9_HUMAN	-2.0533897	0.0400348
Q9Y5X3	SNX5_HUMAN	-2.053956	0.03998
P14868	SYDC_HUMAN	-2.0586805	0.0395249
Q9H4M9	EHD1_HUMAN	-2.0597774	0.0394198
Q12792	TWF1_HUMAN	-2.0637947	0.0390372
Q3MHD2	LSM12_HUMAN	-2.0722738	0.0382399
P35241	RADI_HUMAN	-2.0726028	0.0382093
Q8WUM4	PDC6I_HUMAN	-2.0901231	0.0366067
Q13418	ILK_HUMAN	-2.1039824	0.03538
P07858	CATB_HUMAN	-2.1095475	0.0348973
Q9BQB6	VKOR1_HUMAN	-2.1231497	0.0337413
P14618	KPYM_HUMAN	-2.1239457	0.0336747
P31948	STIP1_HUMAN	-2.1364888	0.0326396
Q96PU8	QKI_HUMAN	-2.1428844	0.0321224
P37802	TAGL2_HUMAN	-2.1462834	0.0318504
Q96CM8	ACSF2_HUMAN	-2.151868	0.0314078
Q9Y2B0	CNPY2_HUMAN	-2.156556	0.0310403
O94804	STK10_HUMAN	-2.1683086	0.0301352
Q9H5X1	FA96A_HUMAN	-2.1741422	0.0296945
Q9UKK9	NUDT5_HUMAN	-2.1776005	0.0294358
P53634	CATC_HUMAN	-2.1806315	0.0292107
Q15843	NEDD8_HUMAN	-2.1819738	0.0291115
Q13561	DCTN2_HUMAN	-2.184173	0.0289495
Q8IVB4	SL9A9_HUMAN	-2.1866284	0.0287697
P04229	2B11_HUMAN	-2.2004423	0.0277755
P52566	GDIR2_HUMAN	-2.2010283	0.027734
O75934	SPF27_HUMAN	-2.201607	0.0276931
P29350	PTN6_HUMAN	-2.2070876	0.0273079
P54578	UBP14_HUMAN	-2.2134909	0.0268638
P09211	GSTP1_HUMAN	-2.2159959	0.0266918
P61981	1433G_HUMAN	-2.2163297	0.0266689
P61158	ARP3_HUMAN	-2.2302362	0.0257318
P21291	CSRP1_HUMAN	-2.235382	0.0253923
O94973	AP2A2_HUMAN	-2.239697	0.0251106
Q9HC35	EMAL4_HUMAN	-2.2432793	0.0248788
Q9BT09	CNPY3_HUMAN	-2.2479115	0.0245818
P51991	ROA3_HUMAN	-2.2622905	0.0236795
P47897	SYQ_HUMAN	-2.2684372	0.0233026
O15145	ARPC3_HUMAN	-2.2733252	0.0230066
Q9UEU0	VTI1B_HUMAN	-2.2762458	0.0228313
O95466	FMNL_HUMAN	-2.2827429	0.0224455
Q14980	NUMA1_HUMAN	-2.2851277	0.0223053
P35610	SOAT1_HUMAN	-2.2861517	0.0222454
Q9Y263	PLAP_HUMAN	-2.3004974	0.0214201
P46109	CRKL_HUMAN	-2.3091134	0.0209373
P20020	AT2B1_HUMAN	-2.309486	0.0209166
Q14103	HNRPD_HUMAN	-2.3394528	0.019312
O75347	TBCA_HUMAN	-2.3509518	0.0187255
P35998	PRS7_HUMAN	-2.3703345	0.017772
P55072	TERA_HUMAN	-2.3753034	0.0175345
P14598	NCF1_HUMAN	-2.4243991	0.0153337
P02794	FRIH_HUMAN	-2.4275765	0.0152001
P19971	TYPH_HUMAN	-2.4281578	0.0151757
P00492	HPRT_HUMAN	-2.4296589	0.015113
Q96CW1	AP2M1_HUMAN	-2.4386066	0.014744

P30049	ATPD_HUMAN	2.0246812	0.0429001
Q9UJU6	DBNL_HUMAN	2.0236331	0.0430079
Q99832	TCPH_HUMAN	2.0206131	0.0433198
Q99541	PLIN2_HUMAN	2.0084305	0.0445976
P30050	RL12_HUMAN	2.0083006	0.0446114
P49755	TMEDA_HUMAN	2.0037024	0.045102
Q969G3	SMCE1_HUMAN	1.998869	0.0456225
O43670	ZN207_HUMAN	1.9911056	0.0464693
P19338	NUCL_HUMAN	1.985502	0.0470886
P63261	ACTG_HUMAN	1.9811997	0.0475689
P17096	HMGA1_HUMAN	1.963447	0.0495943
Q07020	RL18_HUMAN	-1.9807839	0.0476155
P02792	FRIL_HUMAN	-1.9817369	0.0475087
P20036	DPA1_HUMAN	-1.9825842	0.0474139
Q06136	KDSR_HUMAN	-1.98765	0.0468504
Q92542	NICA_HUMAN	-1.9906013	0.0465247
P82921	RT21_HUMAN	-2.0008598	0.0454075
P10620	MGST1_HUMAN	-2.0015805	0.0453299
Q9NVJ2	ARL8B_HUMAN	-2.0018316	0.0453028
P08195	4F2_HUMAN	-2.0116543	0.0442564
Q15366	PCBP2_HUMAN	-2.0222677	0.0431487
P01903	DRA_HUMAN	-2.0250106	0.0428663
O00186	STXB3_HUMAN	-2.026277	0.0427364
Q15904	VAS1_HUMAN	-2.0313974	0.0422147
P04083	ANXA1_HUMAN	-2.0380724	0.0415427
P40939	ECHA_HUMAN	-2.0391581	0.0414343
P08865	RSSA_HUMAN	-2.0483905	0.0405218
O94925	GLSK_HUMAN	-2.0501137	0.0403533
P62861	RS30_HUMAN	-2.054201	0.0399562
Q15738	NSDHL_HUMAN	-2.0654241	0.0388829
P18206	VINC_HUMAN	-2.0665172	0.0387797
O95721	SNP29_HUMAN	-2.0734221	0.038133
Q15363	TMED2_HUMAN	-2.0748326	0.0380021
P02787	TRFE_HUMAN	-2.1021039	0.0355442
Q00610	CLH1_HUMAN	-2.1032538	0.0354436
O00410	IPO5_HUMAN	-2.1114525	0.0347334
P02790	HEMO_HUMAN	-2.1196038	0.0340395
Q9Y5U9	IR3IP_HUMAN	-2.1278754	0.0333474
Q7L014	DDX46_HUMAN	-2.1295738	0.0332068
Q14956	GPNMB_HUMAN	-2.1343113	0.0328173
P13639	EF2_HUMAN	-2.1344895	0.0328027
Q01459	DIAC_HUMAN	-2.1449831	0.0319542
Q2M389	WASH7_HUMAN	-2.1554776	0.0311245
Q9H3K2	GHITM_HUMAN	-2.1693354	0.0300572
Q13217	DNJC3_HUMAN	-2.1758805	0.0295642
Q9NVI7	ATD3A_HUMAN	-2.1804329	0.0292254
Q13724	MOGS_HUMAN	-2.1826889	0.0290587
Q9HB40	RISC_HUMAN	-2.1829096	0.0290425
P02647	APOA1_HUMAN	-2.1890144	0.0285958
Q9NYU2	UGGG1_HUMAN	-2.1906205	0.0284793
P62701	RS4X_HUMAN	-2.2040695	0.0275195
O75410	TACC1_HUMAN	-2.2157492	0.0267087
P07686	HEXB_HUMAN	-2.2198353	0.02643
Q9Y4K1	AIM1_HUMAN	-2.2214048	0.0263236
P07099	HYEP_HUMAN	-2.2218539	0.0262932
Q16181	SEPT7_HUMAN	-2.2238023	0.0261617
P37802	TAGL2_HUMAN	-2.2283393	0.0258579
P62424	RL7A_HUMAN	-2.2285618	0.0258431
Q9Y6A9	SPCS1_HUMAN	-2.2296467	0.0257709
P11279	LAMP1_HUMAN	-2.2325584	0.0255781
Q9BQB6	VKOR1_HUMAN	-2.2482264	0.0245618
P78527	PRKDC_HUMAN	-2.2564425	0.0240429
P18621	RL17_HUMAN	-2.2630955	0.0236298
P47813	IF1AX_HUMAN	-2.26449	0.023544
P17858	K6PL_HUMAN	-2.2656548	0.0234725
P60842	IF4A1_HUMAN	-2.2700611	0.0232039
Q99623	PHB2_HUMAN	-2.2817465	0.0225043
Q9UNM6	PSD13_HUMAN	-2.2970714	0.0216147
P62995	TRA2B_HUMAN	-2.3065007	0.0210827
P02652	APOA2_HUMAN	-2.3102398	0.0208749
P29692	EF1D_HUMAN	-2.3139158	0.0206723
A6NL28	TPM3L_HUMAN	-2.3205406	0.0203117

Q14157	UBP2L_HUMAN	-2.4528725	0.0141721
P41252	SYIC_HUMAN	-2.4561315	0.0140442
P09622	DLDH_HUMAN	-2.4613076	0.0138432
Q6ZUJ8	BCAP_HUMAN	-2.4621128	0.0138121
P16152	CBR1_HUMAN	-2.4710179	0.0134729
P62942	FKB1A_HUMAN	-2.4781009	0.0132084
O75563	SKAP2_HUMAN	-2.4787403	0.0131847
Q06323	PSME1_HUMAN	-2.479944	0.0131403
Q9BVG4	PBDC1_HUMAN	-2.4875199	0.0128637
P62753	RS6_HUMAN	-2.4877678	0.0128548
P40227	TCPZ_HUMAN	-2.4978004	0.0124967
P52209	6PGD_HUMAN	-2.516802	0.0118425
P50552	VASP_HUMAN	-2.5180072	0.0118021
Q9H4A6	GOLP3_HUMAN	-2.5230634	0.0116337
Q969G3	SMCE1_HUMAN	-2.5301062	0.0114028
P39023	RL3_HUMAN	-2.5415312	0.0110368
Q9HB71	CYBP_HUMAN	-2.5512025	0.0107352
P13796	PLSL_HUMAN	-2.5548026	0.0106248
P62750	RL23A_HUMAN	-2.5609113	0.0104398
P46940	IQGA1_HUMAN	-2.5641533	0.0103428
Q9NY33	DPP3_HUMAN	-2.5650673	0.0103156
Q5JWF2	GNAS1_HUMAN	-2.5660674	0.0102859
O00161	SNP23_HUMAN	-2.5718181	0.0101166
Q9UQ80	PA2G4_HUMAN	-2.5724242	0.0100989
O43396	TXNL1_HUMAN	-2.5850004	0.0097379
P50440	GATM_HUMAN	-2.5871274	0.009678
P09601	HMOX1_HUMAN	-2.5921963	0.0095365
P62158	CALM_HUMAN	-2.5957301	0.009439
P04075	ALDOA_HUMAN	-2.5985487	0.0093619
Q9H3G5	CPVL_HUMAN	-2.6111157	0.0090247
P00558	PGK1_HUMAN	-2.6145664	0.0089341
P05413	FABPH_HUMAN	-2.620102	0.0087903
P63208	SKP1_HUMAN	-2.6273309	0.0086058
P07195	LDHB_HUMAN	-2.6372666	0.0083577
Q96T60	PNKP_HUMAN	-2.6466341	0.0081297
P49327	FAS_HUMAN	-2.650803	0.0080301
P19105	ML12A_HUMAN	-2.6671678	0.0076493
Q9UNZ2	NSF1C_HUMAN	-2.6697006	0.0075919
P00390	GSHR_HUMAN	-2.6947783	0.0070435
Q9H269	VPS16_HUMAN	-2.7144062	0.0066395
P26640	SYVC_HUMAN	-2.7156747	0.0066141
Q9H223	EHD4_HUMAN	-2.7204141	0.00652
Q9UJU6	DBNL_HUMAN	-2.7281808	0.0063685
Q8IZ83	A16A1_HUMAN	-2.7319242	0.0062966
Q92882	OSTF1_HUMAN	-2.7342494	0.0062523
O15212	PFD6_HUMAN	-2.7590537	0.0057969
P11766	ADHX_HUMAN	-2.7602654	0.0057754
Q86UP2	KTN1_HUMAN	-2.7638633	0.0057121
P22314	UBA1_HUMAN	-2.7712585	0.005584
P61081	UBC12_HUMAN	-2.7797588	0.0054399
P45974	UBP5_HUMAN	-2.7994705	0.0051186
Q9NQ88	TIGAR_HUMAN	-2.7997163	0.0051148
P01920	DQB1_HUMAN	-2.801121	0.0050925
P11177	ODPB_HUMAN	-2.8058978	0.0050177
Q6ICG6	K0930_HUMAN	-2.8069539	0.0050012
Q16719	KYNU_HUMAN	-2.8105308	0.004946
P50148	GNAQ_HUMAN	-2.8273979	0.0046928
P43034	LIS1_HUMAN	-2.8448917	0.0044427
O60234	GMFG_HUMAN	-2.8450462	0.0044405
P28072	PSB6_HUMAN	-2.8472864	0.0044094
Q9H0W9	CK054_HUMAN	-2.8551786	0.0043013
Q9H4E7	DEFI6_HUMAN	-2.8579187	0.0042643
Q9Y6W5	WASF2_HUMAN	-2.8638755	0.0041849
P68371	TBB4B_HUMAN	-2.8751872	0.0040379
Q9BVL2	NUPL1_HUMAN	-2.8842384	0.0039236
Q99832	TCPH_HUMAN	-2.8880587	0.0038763
Q86UX7	URP2_HUMAN	-2.8953917	0.0037869
O95470	SGPL1_HUMAN	-2.913878	0.0035697
P53367	ARFP1_HUMAN	-2.9177586	0.0035256
O00159	MYO1C_HUMAN	-2.9470491	0.0032082
P25398	RS12_HUMAN	-2.9591162	0.0030852
Q07812	BAX_HUMAN	-2.9898428	0.0027912

O15229	KMO_HUMAN	-2.3437753	0.0190897
P02768	ALBU_HUMAN	-2.3575728	0.0183948
Q00765	REEP5_HUMAN	-2.3604372	0.0182534
Q9BTZ2	DHRS4_HUMAN	-2.3621967	0.018167
P62269	RS18_HUMAN	-2.3695196	0.0178112
Q9BYX2	TBD2A_HUMAN	-2.3762085	0.0174916
Q1KMD3	HNRL2_HUMAN	-2.3931462	0.0167046
Q14974	IMB1_HUMAN	-2.4022164	0.0162961
Q8NBQ5	DHB11_HUMAN	-2.4033946	0.0162436
Q9Y4L1	HYOU1_HUMAN	-2.4058549	0.0161347
P05120	PAI2_HUMAN	-2.4217566	0.0154457
Q9H1C4	UN93B_HUMAN	-2.4243582	0.0153355
O75629	CREG1_HUMAN	-2.4503215	0.0142729
P43003	EAA1_HUMAN	-2.4575637	0.0139883
Q14444	CAPR1_HUMAN	-2.4662882	0.0136521
P26641	EF1G_HUMAN	-2.4870835	0.0128795
P33121	ACSL1_HUMAN	-2.4885173	0.0128277
P15880	RS2_HUMAN	-2.4958576	0.0125653
Q96HY6	DDRGK_HUMAN	-2.5112781	0.0120295
P53674	CRBB1_HUMAN	-2.5141819	0.0119309
P60468	SC61B_HUMAN	-2.5268483	0.0115091
P51148	RAB5C_HUMAN	-2.574549	0.0100371
P52209	6PGD_HUMAN	-2.5785394	0.0099219
P05556	ITB1_HUMAN	-2.6065025	0.0091472
Q13636	RAB31_HUMAN	-2.6096808	0.0090627
Q9P0L0	VAPA_HUMAN	-2.611623	0.0090114
Q96AH8	RAB7B_HUMAN	-2.6181826	0.0088399
Q92688	AN32B_HUMAN	-2.62837	0.0085795
P61353	RL27_HUMAN	-2.6364174	0.0083787
O60749	SNX2_HUMAN	-2.636976	0.0083649
O75131	CPNE3_HUMAN	-2.6410916	0.0082639
P21912	DHSB_HUMAN	-2.661935	0.0077693
Q9Y613	FHOD1_HUMAN	-2.7063064	0.0068036
P49407	ARRB1_HUMAN	-2.7361214	0.0062168
P05362	ICAM1_HUMAN	-2.7374375	0.006192
P01011	AACT_HUMAN	-2.7473423	0.006008
P61026	RAB10_HUMAN	-2.8468589	0.0044153
P68431	H31_HUMAN	-2.8489022	0.004387
O15427	MOT4_HUMAN	-2.8587474	0.0042532
P61313	RL15_HUMAN	-2.8612939	0.0042192
P39019	RS19_HUMAN	-2.8930257	0.0038155
P19838	NFKB1_HUMAN	-2.9972629	0.0027242
Q14728	MFS10_HUMAN	-2.998876	0.0027098
O43252	PAPS1_HUMAN	-3.0103948	0.0026091
P34810	CD68_HUMAN	-3.0123927	0.002592
P02786	TFR1_HUMAN	-3.0206253	0.0025225
P06702	S10A9_HUMAN	-3.0221653	0.0025097
O00232	PSD12_HUMAN	-3.0303769	0.0024425
Q8NBJ5	GT251_HUMAN	-3.035772	0.0023992
P20340	RAB6A_HUMAN	-3.0522344	0.0022714
P04062	GLCM_HUMAN	-3.0556499	0.0022457
P10599	THIO_HUMAN	-3.0962073	0.0019601
P46781	RS9_HUMAN	-3.1502944	0.0016311
Q8N5M9	JAGN1_HUMAN	-3.1601147	0.0015771
P50991	TCPD_HUMAN	-3.219053	0.0012861
P23246	SFPQ_HUMAN	-3.2217497	0.0012741
O14979	HNRDL_HUMAN	-3.2669401	0.0010872
P50416	CPT1A_HUMAN	-3.3222753	0.0008929
P05023	AT1A1_HUMAN	-3.3465119	0.0008184
Q9BSJ8	ESYT1_HUMAN	-3.4105997	0.0006482
P05090	APOD_HUMAN	-3.4185193	0.0006296
P01009	A1AT_HUMAN	-3.4572023	0.0005458
Q9BTU6	P4K2A_HUMAN	-3.4642218	0.0005318
P62081	RS7_HUMAN	-3.4950376	0.000474
Q99685	MGLL_HUMAN	-3.5074974	0.0004523
P47756	CAPZB_HUMAN	-3.5119947	0.0004448
P46783	RS10_HUMAN	-3.561492	0.0003688
P04844	RPN2_HUMAN	-3.5674604	0.0003605
P61247	RS3A_HUMAN	-3.5791347	0.0003447
P63244	GBLP_HUMAN	-3.5956819	0.0003235
Q9BZF1	OSBL8_HUMAN	-3.6052267	0.0003119
P43490	NAMPT_HUMAN	-3.6449866	0.0002674

Q16543	CDC37_HUMAN	-2.9911854	0.002779
P50502	F10A1_HUMAN	-3.0126463	0.0025898
Q7Z4G1	COMD6_HUMAN	-3.0190807	0.0025354
O60313	OPA1_HUMAN	-3.0566936	0.0022379
P15090	FABP4_HUMAN	-3.0663183	0.0021671
P49368	TCPG_HUMAN	-3.085176	0.0020343
Q9BRF8	CPPED_HUMAN	-3.1005582	0.0019316
Q96C23	GALM_HUMAN	-3.100733	0.0019304
P08575	PTPRC_HUMAN	-3.1082265	0.0018821
P84098	RL19_HUMAN	-3.1146425	0.0018417
P20042	IF2B_HUMAN	-3.1219282	0.0017967
P04233	HG2A_HUMAN	-3.1222699	0.0017946
P37837	TALDO_HUMAN	-3.1292842	0.0017523
O60493	SNX3_HUMAN	-3.1308237	0.0017432
P05109	S10A8_HUMAN	-3.1398636	0.0016903
Q969T9	WBP2_HUMAN	-3.1435679	0.001669
P00338	LDHA_HUMAN	-3.144416	0.0016642
Q02543	RL18A_HUMAN	-3.1487076	0.0016399
Q658P3	STEA3_HUMAN	-3.1673025	0.0015386
O00170	AIP_HUMAN	-3.1968723	0.0013893
Q9H2H8	PPIL3_HUMAN	-3.2059358	0.0013462
Q07866	KLC1_HUMAN	-3.217882	0.0012914
P33176	KINH_HUMAN	-3.2209696	0.0012776
P62304	RUXE_HUMAN	-3.2211432	0.0012768
P36969	GPX4_HUMAN	-3.2512745	0.0011489
P20702	ITAX_HUMAN	-3.2519843	0.001146
Q9HC38	GLOD4_HUMAN	-3.2706936	0.0010728
Q9BZG1	RAB34_HUMAN	-3.2708683	0.0010722
P18669	PGAM1_HUMAN	-3.3032091	0.0009559
P58546	MTPN_HUMAN	-3.3281047	0.0008744
O43491	E41L2_HUMAN	-3.3321333	0.0008618
Q15080	NCF4_HUMAN	-3.3368621	0.0008473
P25685	DNJB1_HUMAN	-3.3469247	0.0008171
P60983	GMFB_HUMAN	-3.3514877	0.0008038
P31946	1433B_HUMAN	-3.3608591	0.000777
Q9Y490	TLN1_HUMAN	-3.3610603	0.0007764
O75083	WDR1_HUMAN	-3.3985759	0.0006774
Q15067	ACOX1_HUMAN	-3.4152874	0.0006371
Q8TAT6	NPL4_HUMAN	-3.4202937	0.0006255
P27105	STOM_HUMAN	-3.4257516	0.0006131
Q15427	SF3B4_HUMAN	-3.4720176	0.0005166
O75351	VPS4B_HUMAN	-3.5078394	0.0004518
P31150	GDIA_HUMAN	-3.5374812	0.000404
Q9UJ70	NAGK_HUMAN	-3.5627439	0.000367
P52565	GDIR1_HUMAN	-3.5643137	0.0003648
Q96TA1	NIBL1_HUMAN	-3.5865107	0.0003351
P40429	RL13A_HUMAN	-3.6109843	0.000305
Q71UM5	RS27L_HUMAN	-3.6246384	0.0002894
P52790	HXK3_HUMAN	-3.6487924	0.0002635
Q9UHL4	DPP2_HUMAN	-3.6511465	0.0002611
P31153	METK2_HUMAN	-3.6511927	0.000261
P51570	GALK1_HUMAN	-3.6555308	0.0002567
Q9NQW7	XPP1_HUMAN	-3.7138605	0.0002041
Q92835	SHIP1_HUMAN	-3.7166612	0.0002019
P62913	RL11_HUMAN	-3.7231398	0.0001968
P05362	ICAM1_HUMAN	-3.7240838	0.000196
Q9NUN5	LMBD1_HUMAN	-3.7261242	0.0001944
P49189	AL9A1_HUMAN	-3.744391	0.0001808
P63220	RS21_HUMAN	-3.7544405	0.0001737
P62263	RS14_HUMAN	-3.772619	0.0001615
Q99497	PARK7_HUMAN	-3.7762843	0.0001592
O00160	MYO1F_HUMAN	-3.7852101	0.0001536
O95498	VNN2_HUMAN	-3.8050274	0.0001418
P23526	SAHH_HUMAN	-3.8094975	0.0001392
Q9ULA0	DNPEP_HUMAN	-3.8234526	0.0001316
P30038	AL4A1_HUMAN	-3.8426689	0.0001217
P14174	MIF_HUMAN	-3.8441392	0.000121
P15311	EZRI_HUMAN	-3.8569608	0.0001148
Q14696	MESD_HUMAN	-3.8651253	0.000111
O95336	6PGL_HUMAN	-3.9401628	8.143E-05
Q9Y333	LSM2_HUMAN	-3.9473024	7.904E-05
O95865	DDAH2_HUMAN	-3.9643965	7.358E-05

P17844	DDX5_HUMAN	-3.6690527	0.0002435
Q9H8H3	MET7A_HUMAN	-3.6707047	0.0002419
P24534	EF1B_HUMAN	-3.845923	0.0001201
P04839	CY24B_HUMAN	-3.8939698	9.862E-05
Q9Y285	SYFA_HUMAN	-3.9187377	8.901E-05
Q9BWJ5	SF3B5_HUMAN	-3.9287751	8.538E-05
P30101	PDIA3_HUMAN	-4.2782307	1.884E-05
P60953	CDC42_HUMAN	-4.4504757	8.568E-06
P0CG06	LAC3_HUMAN	-4.5083929	6.532E-06
Q92552	RT27_HUMAN	-4.8945758	9.852E-07
P36578	RL4_HUMAN	-4.9854078	6.183E-07
Q9NZ01	TECR_HUMAN	-5.5790831	2.418E-08
P16671	CD36_HUMAN	-5.9273458	3.079E-09
Q9Y4I1	MYO5A_HUMAN	-6.2056124	5.448E-10
Q9UNX3	RL26L_HUMAN	-6.6132972	3.759E-11
P46776	RL27A_HUMAN	-7.0983114	1.263E-12
Q14643	ITPR1_HUMAN	-7.1033754	1.217E-12
Q13838	DX39B_HUMAN	-7.5461207	4.485E-14
P15121	ALDR_HUMAN	-7.9335769	2.22E-15
Q6NUT3	MFS12_HUMAN	-9.9105629	0
P04179	SODM_HUMAN	-10.645584	0

P29401	TKT_HUMAN	-3.9963989	6.431E-05
P50395	GDIB_HUMAN	-4.0071986	6.144E-05
P56211	ARP19_HUMAN	-4.0073818	6.14E-05
P17987	TCPA_HUMAN	-4.0075174	6.136E-05
P49588	SYAC_HUMAN	-4.0102867	6.065E-05
Q8TDZ2	MICA1_HUMAN	-4.0392387	5.362E-05
P31146	COR1A_HUMAN	-4.0523797	5.07E-05
Q9Y286	SIGL7_HUMAN	-4.087635	4.358E-05
Q9BY43	CHM4A_HUMAN	-4.0902964	4.308E-05
Q9HAB8	PPCS_HUMAN	-4.1087963	3.977E-05
P53602	MVD1_HUMAN	-4.1174099	3.832E-05
P30740	ILEU_HUMAN	-4.1202812	3.784E-05
Q9Y679	AUP1_HUMAN	-4.1506953	3.315E-05
Q9H4A4	AMPB_HUMAN	-4.1534874	3.274E-05
Q9NUQ9	FA49B_HUMAN	-4.1873736	2.822E-05
P61163	ACTZ_HUMAN	-4.1933925	2.748E-05
Q9BVC6	TM109_HUMAN	-4.2043852	2.618E-05
O00233	PSMD9_HUMAN	-4.2170843	2.475E-05
O60664	PLIN3_HUMAN	-4.2250986	2.388E-05
P55957	BID_HUMAN	-4.2372754	2.262E-05
P30041	PRDX6_HUMAN	-4.2821103	1.851E-05
Q96KP4	CNDP2_HUMAN	-4.2904768	1.783E-05
P49721	PSB2_HUMAN	-4.3080085	1.647E-05
P01040	CYTA_HUMAN	-4.3197326	1.562E-05
P60981	DEST_HUMAN	-4.3394431	1.428E-05
Q9Y5P6	GMPPB_HUMAN	-4.4381986	9.071E-06
O95232	LC7L3_HUMAN	-4.4694717	7.841E-06
P08571	CD14_HUMAN	-4.4699485	7.824E-06
P61160	ARP2_HUMAN	-4.5455309	5.48E-06
P00352	AL1A1_HUMAN	-4.5601402	5.112E-06
P15586	GNS_HUMAN	-4.5633951	5.033E-06
P30086	PEBP1_HUMAN	-4.6035984	4.153E-06
P26006	ITA3_HUMAN	-4.6083973	4.058E-06
Q9UBQ0	VPS29_HUMAN	-4.6556194	3.23E-06
P62258	1433E_HUMAN	-4.7755646	1.792E-06
Q03169	TNAP2_HUMAN	-4.7884615	1.681E-06
P52907	CAZA1_HUMAN	-4.8120227	1.494E-06
P35237	SPB6_HUMAN	-4.8237903	1.409E-06
P50897	PPT1_HUMAN	-4.935073	8.012E-07
P98082	DAB2_HUMAN	-4.9481127	7.494E-07
Q14165	MLEC_HUMAN	-4.9854718	6.181E-07
P09960	LKHA4_HUMAN	-4.987872	6.105E-07
Q9Y2S7	PDIP2_HUMAN	-4.9956142	5.865E-07
Q96QK1	VPS35_HUMAN	-5.0742624	3.89E-07
P43487	RANG_HUMAN	-5.0831129	3.713E-07
P07737	PROF1_HUMAN	-5.0833186	3.709E-07
P04080	CYTB_HUMAN	-5.0908256	3.565E-07
Q9NRV9	HEBP1_HUMAN	-5.1638725	2.419E-07
P27635	RL10_HUMAN	-5.1691244	2.352E-07
P40925	MDHC_HUMAN	-5.1906102	2.096E-07
Q9BXD5	NPL_HUMAN	-5.2150727	1.837E-07
P61086	UBE2K_HUMAN	-5.217577	1.813E-07
P62993	GRB2_HUMAN	-5.276161	1.319E-07
P15121	ALDR_HUMAN	-5.2832794	1.269E-07
Q01469	FABP5_HUMAN	-5.2956485	1.186E-07
P42566	EPS15_HUMAN	-5.3020616	1.145E-07
Q66K14	TBC9B_HUMAN	-5.3267898	9.996E-08
P28066	PSA5_HUMAN	-5.3461492	8.985E-08
P13798	ACPH_HUMAN	-5.4420449	5.267E-08
P50990	TCPQ_HUMAN	-5.5928294	2.234E-08
P14550	AK1A1_HUMAN	-5.6262909	1.841E-08
P25098	ARBK1_HUMAN	-5.6457487	1.645E-08
P06733	ENOA_HUMAN	-5.6636354	1.482E-08
P54725	RD23A_HUMAN	-5.7111786	1.122E-08
Q6IBS0	TWF2_HUMAN	-5.7450135	9.191E-09
P61970	NTF2_HUMAN	-5.7568027	8.572E-09
Q15056	IF4H_HUMAN	-5.7771514	7.598E-09
Q96JJ3	ELMO2_HUMAN	-5.8345598	5.393E-09
P08107	HSP71_HUMAN	-5.8612736	4.593E-09
O94903	PROSC_HUMAN	-6.0192517	1.752E-09
P50452	SPB8_HUMAN	-6.0426514	1.516E-09
P26038	MOES_HUMAN	-6.1624468	7.163E-10

P13489	RINI_HUMAN	-6.1716061	6.76E-10
O75368	SH3L1_HUMAN	-6.2120157	5.231E-10
O43707	ACTN4_HUMAN	-6.3933847	1.623E-10
Q9NY15	STAB1_HUMAN	-6.9512667	3.62E-12
O75369	FLNB_HUMAN	-7.0883327	1.357E-12
Q96CX2	KCD12_HUMAN	-7.1500916	8.673E-13
P19878	NCF2_HUMAN	-7.2067119	5.731E-13
P35579	MYH9_HUMAN	-7.4140527	1.226E-13
Q96L92	SNX27_HUMAN	-7.7014176	1.354E-14
P12955	PEPD_HUMAN	-8.0875159	6.661E-16
Q9NR45	SIAS_HUMAN	-8.1282264	4.441E-16
P01034	CYTC_HUMAN	-8.5746634	0
Q06830	PRDX1_HUMAN	-8.5976735	0
Q9BZE4	NOG1_HUMAN	-8.7414793	0
P23528	COF1_HUMAN	-8.7442779	0
O00483	NDUA4_HUMAN	-9.2766215	0
P07900	HS90A_HUMAN	-9.3175739	0
O43399	TPD54_HUMAN	-9.8021114	0
O75874	IDHC_HUMAN	-10.279078	0
Q9H299	SH3L3_HUMAN	-10.31234	0
Q13596	SNX1_HUMAN	-10.830721	0
Q86VB7	C163A_HUMAN	-11.14496	0
Q15691	MARE1_HUMAN	-11.50266	0
P48643	TCPE_HUMAN	-11.524573	0
P46926	GNPI1_HUMAN	-11.764431	0
P62937	PPIA_HUMAN	-11.99255	0
Q10567	AP1B1_HUMAN	-12.216376	0
P57737	CORO7_HUMAN	-14.360583	0
