# Supplementary figures and images for: Opposing regulation of endolysosomal pathways by long-acting nanoformulated antiretroviral therapy and HIV-1 in human macrophages
Source: Retrovirology. 2015 Jan 22;12:5. doi: 10.1186/s12977-014-0133-5 (PMC4307176; doi:10.1186/s12977-014-0133-5)

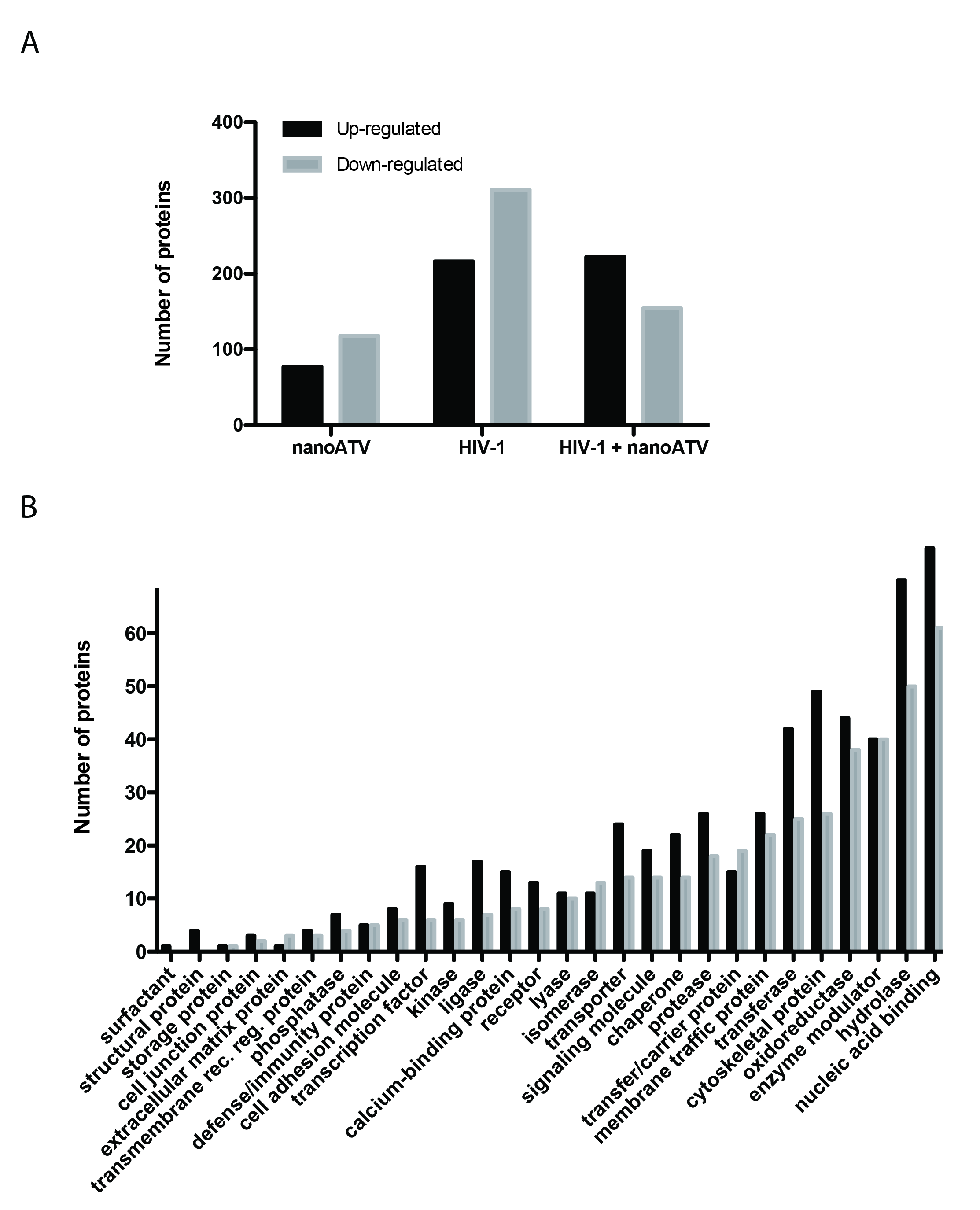

Supplement: Additional file 2: — Deregulated proteins during HIV-1 infection and nanoATV treatment. Uninfected and HIV-1 infected MDM were treated with or without nanoATV. The cells were then collected for peptide identification. (A) Numbers of significantly up- or down- regulated proteins were identified by SWATH-MS and compared to uninfected and untreated MDM used as controls. (B) Changed proteins were classified according to protein class by PANTHER. These are represented by 28 independent clusters (p<0.05). [file 12977_2014_133_MOESM2_ESM.tiff]

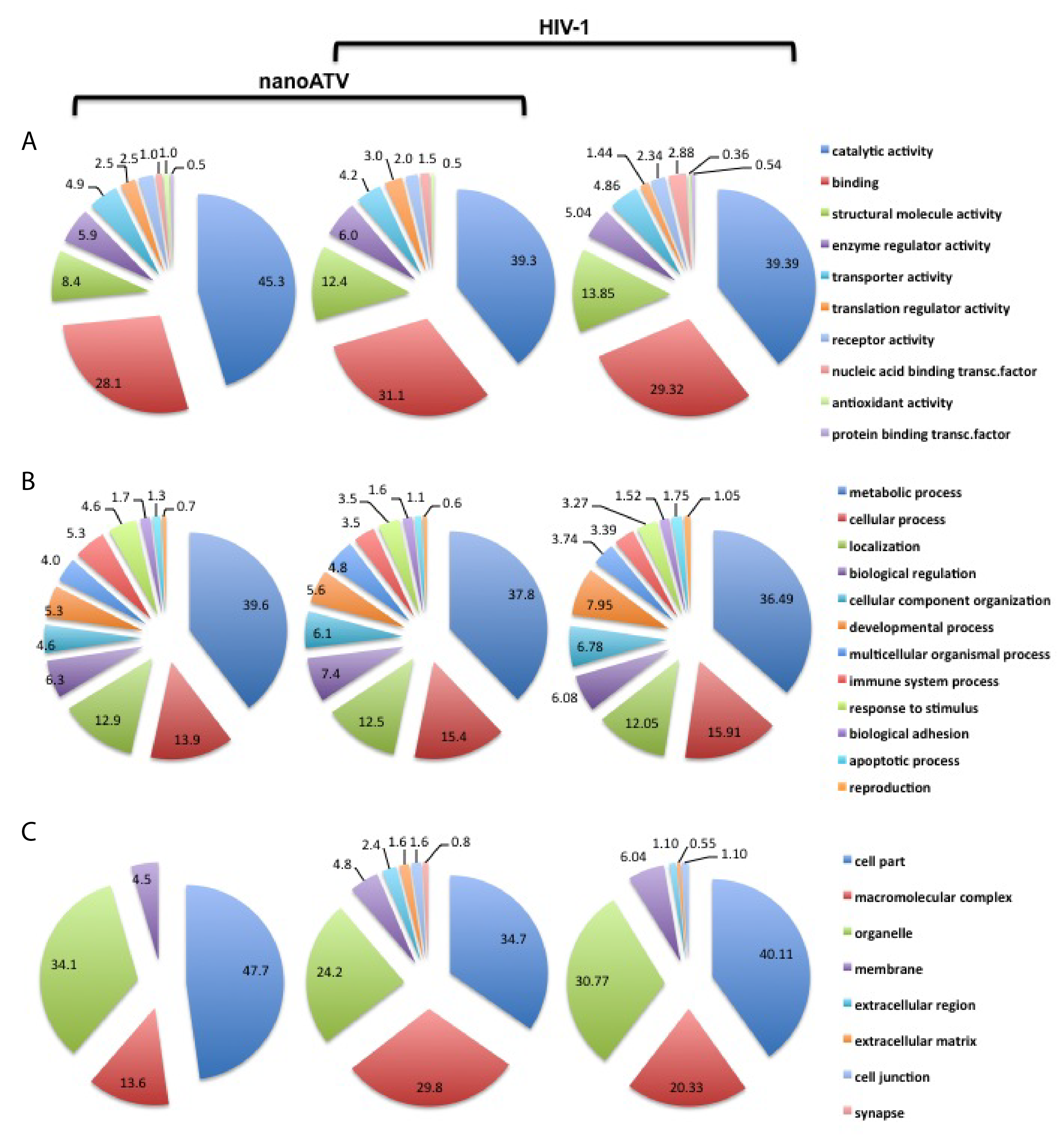

Supplement: Additional file 3: — Functional characterization of significant deregulated proteins between uninfected and HIV-1-infected and nanoATV treated MDM. Proteins were compared to uninfected/untreated control cells (p<0.05) then bioinformatics analysis was performed. The Gene Ontology molecular function (A), biological processes (B) and cellular component distribution (C) were obtained from the analysis using the Protein Analysis Through Evolutionary Relationships (PANTHER) classification system. [file 12977_2014_133_MOESM3_ESM.tiff]
