# Supplementary material for: Opposing regulation of endolysosomal pathways by long-acting nanoformulated antiretroviral therapy and HIV-1 in human macrophages
Source: Retrovirology. 2015 Jan 22;12:5. doi: 10.1186/s12977-014-0133-5 (PMC4307176; doi:10.1186/s12977-014-0133-5)
Supplement: Additional file 4: — Annotation Clusters and Enrichment data for MDM treated with nanoATV using DAVID. [file 12977_2014_133_MOESM4_ESM.pdf]

Additional file 4. Annotation Clusters and Enrichment data for MDM treated with nanoATV using DAVID

Cluster and Category	Enrichment and Term	Count	%	P value	Genes	Fold Enrichment
Annotation Cluster 1	***Enrichment Score: 9.459341796686921***					
GOTERM_CC_FAT	GO:0031967~organelle envelope	32	16.49485	7.33E-11	P24752, P27105, Q14974, Q99714, P33121, P09874, P15153, P18859, Q7Z3B4, P50440, P35914, Q9Y4W6, P40926, O75489, P42765, P12236, P20674, Q9NZM1, Q9Y5L4, O15229, P13073, P31930, P10809, P48047, P04179, P50416, O43169, Q9BVC6, P25705, P55957, Q16836, P20073	3.95039598
GOTERM_CC_FAT	GO:0031975~envelope	32	16.49485	7.95E-11	P24752, P27105, Q14974, Q99714, P33121, P09874, P15153, P18859, Q7Z3B4, P50440, P35914, Q9Y4W6, P40926, O75489, P42765, P12236, P20674, Q9NZM1, Q9Y5L4, O15229, P13073, P31930, P10809, P48047, P04179, P50416, O43169, Q9BVC6, P25705, P55957, Q16836, P20073	3.93769374
GOTERM_CC_FAT	GO:0031966~mitochondrial membrane	25	12.8866	2.63E-10	P24752, P27105, Q99714, P33121, P18859, P50440, P35914, Q9Y4W6, P40926, O75489, P42765, P12236, P20674, Q9Y5L4, O15229, P13073, P31930, P10809, P48047, P04179, P50416, O43169, P25705, P55957, Q16836	4.85653059
GOTERM_CC_FAT	GO:0005743~mitochondrial inner membrane	22	11.34021	4.41E-10	P24752, Q9Y4W6, P40926, O75489, P42765, P12236, P20674, Q99714, Q9Y5L4, O15229, P13073, P31930, P10809, P04179, P50416, P48047, O43169, P25705, P18859, P50440, Q16836, P35914	5.50279832
GOTERM_CC_FAT	GO:0044429~mitochondrial part	30	15.46392	5.99E-10	P24752, P27105, Q99714, P33121, P11310, P18859, P50440, P35914, Q9Y4W6, P40926, O75489, P42765, P12236, P20674, Q9Y5L4, O14874, P38646, O15229, P13073, P31930, P10809, P48047, P04179, P50416, O43169, P25705, P82650, P50213, P55957, Q16836	3.85910532
GOTERM_CC_FAT	GO:0005740~mitochondrial envelope	25	12.8866	9.13E-10	P24752, P27105, Q99714, P33121, P18859, P50440, P35914, Q9Y4W6, P40926, O75489, P42765, P12236, P20674, Q9Y5L4, O15229, P13073, P31930, P10809, P48047, P04179, P50416, O43169, P25705, P55957, Q16836	4.56676147
GOTERM_CC_FAT	GO:0019866~organelle inner membrane	22	11.34021	1.65E-09	P24752, Q9Y4W6, P40926, O75489, P42765, P12236, P20674, Q99714, Q9Y5L4, O15229, P13073, P31930, P10809, P04179, P50416, P48047, O43169, P25705, P18859, P50440, Q16836, P35914	5.11810422
Annotation Cluster 2	***Enrichment Score: 3.9467739581145262***					
GOTERM_BP_FAT	GO:0015031~protein transport	24	12.37113	9.84E-05	Q86VS8, P30101, Q13596, Q14974, P49841, P49755, Q8WUM4, P27797, Q9NP72, P30040, Q9Y5L4, Q9H0U4, O75436, P38646, P50395, P20339, P18085, Q86Y82, P16671, Q7Z3B4, O95721, P31150, P55957, O14828	2.46288289
GOTERM_BP_FAT	GO:0045184~establishment of protein localization	24	12.37113	1.13E-04	Q86VS8, P30101, Q13596, Q14974, P49841, P49755, Q8WUM4, P27797, Q9NP72, P30040, Q9Y5L4, Q9H0U4, O75436, P38646, P50395, P20339, P18085, Q86Y82, P16671, Q7Z3B4, O95721, P31150, P55957, O14828	2.44046393
GOTERM_BP_FAT	GO:0008104~protein localization	26	13.40206	1.30E-04	Q13596, Q14974, P27797, O75436, P18085, P15311, Q7Z3B4, O14828, Q86VS8, P30101, P60033, P49841, P49755, Q8WUM4, Q9NP72, P30040, Q9H0U4, Q9Y5L4, P38646, P20339, P50395, Q86Y82, P16671, O95721, P55957, P31150	2.30511318
Annotation Cluster 3	***Enrichment Score: 3.5314963387724307***					
INTERPRO	IPR002155:Thiolase	4	2.061856	4.64E-05	P24752, P22307, P42765, Q9BWD1	50.9060351
INTERPRO	IPR016038:Thiolase-like, subgroup	4	2.061856	1.09E-04	P24752, P22307, P42765, Q9BWD1	39.5935829
PIR_SUPERFAMILY	PIRSF000429:Ac-CoA_Ac_transf	4	2.061856	1.65E-04	P24752, P22307, P42765, Q9BWD1	33.0178571
GOTERM_MF_FAT	GO:0016408~C-acyltransferase activity	4	2.061856	9.65E-04	P24752, P22307, P42765, Q9BWD1	19.7911585
UP_SEQ_FEATURE	active site:Acyl-thioester intermediate	3	1.546392	0.002729	P24752, P42765, Q9BWD1	36.945232
Annotation Cluster 4	***Enrichment Score: 3.131951564786724***					
GOTERM_MF_FAT	GO:0015078~hydrogen ion transmembrane transporter activity	8	4.123711	1.35E-04	P13073, P31930, P48047, P25705, P18859, P20674, P21281, Q93050	7.03685637
GOTERM_MF_FAT	GO:0015077~monovalent inorganic cation transmembrane transporter activity	8	4.123711	3.31E-04	P13073, P31930, P48047, P25705, P18859, P20674, P21281, Q93050	6.08958724
KEGG_PATHWAY	hsa00190:Oxidative phosphorylation	9	4.639175	0.002258	P13073, P31930, O75489, P48047, P25705, P18859, P20674, P21281, Q93050	3.7853598
GOTERM_MF_FAT	GO:0022890~inorganic cation transmembrane transporter activity	8	4.123711	0.002945	P13073, P31930, P48047, P25705, P18859, P20674, P21281, Q93050	4.1941528

Annotation Cluster 5	***Enrichment Score: 2.9387803714893956***					
GOTERM_CC_FAT	GO:0016023~cytoplasmic membrane-bounded vesicle	18	9.278351	8.05E-04	P30101, P31146, P27105, P49755, Q8WUM4, P30040, P21281, P23526, Q93050, P20339, P08195, P10809, Q86Y82, P10909, P16671, Q14956, Q06830	2.50491018
GOTERM_CC_FAT	GO:0031982~vesicle	20	10.30928	0.001113	P30101, P31146, P27105, P49755, Q8WUM4, P30040, Q9NZM1, P21281, P23526, Q93050, P20339, P08195, P10809, Q86Y82, P10909, P16671, Q14956, P20073, Q06830	2.28474394
GOTERM_CC_FAT	GO:0031988~membrane-bounded vesicle	18	9.278351	0.001147	P30101, P31146, P27105, P49755, Q8WUM4, P30040, P21281, P23526, Q93050, P20339, P08195, P10809, Q86Y82, P10909, P16671, Q14956, Q06830	2.42552922
GOTERM_CC_FAT	GO:0031410~cytoplasmic vesicle	19	9.793814	0.00171	P30101, P31146, P27105, P49755, Q8WUM4, P30040, Q9NZM1, P21281, P23526, Q93050, P20339, P08195, P10809, Q86Y82, P10909, P16671, Q14956, Q06830	2.26517059
**Annotation Cluster 6**	**Enrichment Score: 2.779307836775372**					
INTERPRO	IPR017936:Thioredoxin-like	5	2.57732	6.70E-04	P30101, P10599, Q8NBS9, Q13162, Q06830	12.3729947
SP_PIR_KEYWORDS	Redox-active center	5	2.57732	8.16E-04	P30101, P10599, Q8NBS9, Q13162, Q06830	11.8035101
GOTERM_BP_FAT	GO:0045454~cell redox homeostasis	5	2.57732	0.008395	P30101, P10599, Q8NBS9, Q13162, Q06830	6.20607395
Annotation Cluster 7	***Enrichment Score: 2.6480329334270434***					
INTERPRO	IPR015048:Region of unknown function DUF1899	3	1.546392	0.001806	P31146, Q9ULV4, Q9BR76	44.5427807
INTERPRO	IPR015049:Region of unknown function DUF1900	3	1.546392	0.00251	P31146, Q9ULV4, Q9BR76	38.1795264
INTERPRO	IPR015505:Coronin	3	1.546392	0.00251	P31146, Q9ULV4, Q9BR76	38.1795264
Annotation Cluster 8	***Enrichment Score: 2.545193972931817***					
GOTERM_BP_FAT	GO:0005996~monosaccharide metabolic process	10	5.154639	2.14E-03	Q06210, P40925, P40926, P49841, P00558, P50416, P11216, Q9UKK9, P46976, O00757	3.5223663
GOTERM_BP_FAT	GO:0019318~hexose metabolic process	9	4.639175	0.003171	Q06210, P40925, P40926, P49841, P00558, P50416, P11216, P46976, O00757	3.66546243
GOTERM_BP_FAT	GO:0006006~glucose metabolic process	8	4.123711	0.003409	P40925, P40926, P49841, P00558, P50416, P11216, P46976, O00757	4.08870754
Annotation Cluster 9	***Enrichment Score: 2.5071464857795256***					
GOTERM_BP_FAT	GO:0009060~aerobic respiration	5	2.57732	9.71E-04	P40925, P31930, P40926, P50213, P48735	11.1709331
SP_PIR_KEYWORDS	tricarboxylic acid cycle	4	2.061856	0.001158	P40925, P40926, P50213, P48735	18.8856161
GOTERM_BP_FAT	GO:0046356~acetyl-CoA catabolic process	4	2.061856	0.002967	P40925, P40926, P50213, P48735	13.5993968
GOTERM_BP_FAT	GO:0006099~tricarboxylic acid cycle	4	2.061856	0.002967	P40925, P40926, P50213, P48735	13.5993968
GOTERM_BP_FAT	GO:0009109~coenzyme catabolic process	4	2.061856	0.004235	P40925, P40926, P50213, P48735	12.0302357
GOTERM_BP_FAT	GO:0006734~NADH metabolic process	3	1.546392	0.004279	P40925, P40926, P50213	29.3236994
GOTERM_BP_FAT	GO:0006084~acetyl-CoA metabolic process	4	2.061856	6.99E-03	P40925, P40926, P50213, P48735	10.0898751
GOTERM_BP_FAT	GO:0051187~cofactor catabolic process	4	2.061856	0.006991	P40925, P40926, P50213, P48735	10.0898751
Annotation Cluster 10	***Enrichment Score: 2.3632561689261466***					
GOTERM_MF_FAT	GO:0046933~hydrogen ion transporting ATP synthase activity, rotational mechanism	4	2.061856	9.65E-04	P48047, P25705, P18859, P21281	19.7911585
SP_PIR_KEYWORDS	Hydrogen ion transport	5	2.57732	0.001354	P48047, P25705, P18859, P21281, Q93050	10.3280713
GOTERM_BP_FAT	GO:0015985~energy coupled proton transport, down electrochemical gradient	5	2.57732	0.001614	P48047, P25705, P18859, P21281, Q93050	9.77456647

GOTERM_BP_FAT	GO:0015986~ATP synthesis coupled proton transport	5	2.57732	0.001614	P48047, P25705, P18859, P21281, Q93050	9.77456647
GOTERM_CC_FAT	GO:0016469~proton-transporting two-sector ATPase complex	5	2.57732	0.002701	P48047, P25705, P18859, P21281, Q93050	8.50432468
GOTERM_BP_FAT	GO:0009259~ribonucleotide metabolic process	8	4.123711	0.002728	P48047, P25705, P18859, Q9H7F0, Q9UKK9, P21281, P00813, Q93050	4.25559357
GOTERM_MF_FAT	GO:0046961~proton-transporting ATPase activity, rotational mechanism	4	2.061856	0.002861	P48047, P25705, P18859, P21281	13.7677625
GOTERM_BP_FAT	GO:0034220~ion transmembrane transport	5	2.57732	0.003425	P48047, P25705, P18859, P21281, Q93050	7.97923794
GOTERM_BP_FAT	GO:0009152~purine ribonucleotide biosynthetic process	7	3.608247	0.003786	P48047, P25705, P18859, Q9H7F0, P21281, P00813, Q93050	4.67842498
GOTERM_BP_FAT	GO:0009144~purine nucleoside triphosphate metabolic process	7	3.608247	0.004646	P48047, P25705, P18859, Q9H7F0, P21281, P00813, Q93050	4.48668625
GOTERM_BP_FAT	GO:0009260~ribonucleotide biosynthetic process	7	3.608247	0.005028	P48047, P25705, P18859, Q9H7F0, P21281, P00813, Q93050	4.41432034
GOTERM_BP_FAT	GO:0006754~ATP biosynthetic process	6	3.092784	0.005509	P48047, P25705, P18859, Q9H7F0, P21281, Q93050	5.2716763
GOTERM_BP_FAT	GO:0009141~nucleoside triphosphate metabolic process	7	3.608247	0.006546	P48047, P25705, P18859, Q9H7F0, P21281, P00813, Q93050	4.17844063
GOTERM_BP_FAT	GO:0015992~proton transport	5	2.57732	0.0075	P48047, P25705, P18859, P21281, Q93050	6.40955179
GOTERM_BP_FAT	GO:0009206~purine ribonucleoside triphosphate biosynthetic process	6	3.092784	8.23E-03	P48047, P25705, P18859, Q9H7F0, P21281, Q93050	4.78754276
GOTERM_BP_FAT	GO:0009150~purine ribonucleotide metabolic process	7	3.608247	0.008372	P48047, P25705, P18859, Q9H7F0, P21281, P00813, Q93050	3.96649074
GOTERM_BP_FAT	GO:0006818~hydrogen transport	5	2.57732	0.008395	P48047, P25705, P18859, P21281, Q93050	6.20607395
GOTERM_BP_FAT	GO:0009145~purine nucleoside triphosphate biosynthetic process	6	3.092784	0.008581	P48047, P25705, P18859, Q9H7F0, P21281, Q93050	4.73918374
GOTERM_BP_FAT	GO:0009201~ribonucleoside triphosphate biosynthetic process	6	3.092784	0.008581	P48047, P25705, P18859, Q9H7F0, P21281, Q93050	4.73918374
GOTERM_BP_FAT	GO:0009142~nucleoside triphosphate biosynthetic process	6	3.092784	0.009695	P48047, P25705, P18859, Q9H7F0, P21281, Q93050	4.59979599
GOTERM_BP_FAT	GO:0009165~nucleotide biosynthetic process	8	4.123711	0.009698	P48047, P25705, P18859, Q9H7F0, P21281, P00813, Q93050, O15229	3.36329169
Annotation Cluster 11	***Enrichment Score: 2.177831156434542***					
GOTERM_BP_FAT	GO:0031400~negative regulation of protein modification process	7	3.608247	0.004115	P35998, P27105, P25786, P62942, P17980, P55036, P51665	4.59979599
GOTERM_BP_FAT	GO:0031398~positive regulation of protein ubiquitination	6	3.092784	0.00431	P35998, P25786, P62942, P17980, P55036, P51665	5.58546656
GOTERM_BP_FAT	GO:0032269~negative regulation of cellular protein metabolic process	8	4.123711	0.008177	P35998, P27105, P25786, P62942, P17980, P27797, P55036, P51665	3.47540141
GOTERM_BP_FAT	GO:0031396~regulation of protein ubiquitination	6	3.092784	0.008942	P35998, P25786, P62942, P17980, P55036, P51665	4.69179191
GOTERM_BP_FAT	GO:0051248~negative regulation of protein metabolic process	8	4.123711	0.009953	P35998, P27105, P25786, P62942, P17980, P27797, P55036, P51665	3.34530617
Annotation Cluster 12	***Enrichment Score: 2.169979087961855***					
SP_PIR_KEYWORDS	proteasome	5	2.57732	0.002402	P35998, P25786, P17980, P55036, P51665	8.85263255

GOTERM_BP_FAT	GO:0031398~positive regulation of protein ubiquitination	6	3.092784	0.00431	P35998, P25786, P62942, P17980, P55036, P51665	5.58546656
GOTERM_CC_FAT	GO:0000502~proteasome complex	5	2.57732	0.00806	P35998, P25786, P17980, P55036, P51665	6.27368214
GOTERM_BP_FAT	GO:0031396~regulation of protein ubiquitination	6	3.092784	0.008942	P35998, P25786, P62942, P17980, P55036, P51665	4.69179191
GOTERM_BP_FAT	GO:0051436~negative regulation of ubiquitin-protein ligase activity during mitotic cell cycle	5	2.57732	0.009357	P35998, P25786, P17980, P55036, P51665	6.01511783
GOTERM_BP_FAT	GO:0031145~anaphase-promoting complex-dependent proteasomal ubiquitin-dependent protein catabolic process	5	2.57732	0.009357	P35998, P25786, P17980, P55036, P51665	6.01511783
KEGG_PATHWAY	hsa03050:Proteasome	5	2.57732	0.009887	P35998, P25786, P17980, P55036, P51665	5.81674674
