# Supplementary material for: Opposing regulation of endolysosomal pathways by long-acting nanoformulated antiretroviral therapy and HIV-1 in human macrophages
Source: Retrovirology. 2015 Jan 22;12:5. doi: 10.1186/s12977-014-0133-5 (PMC4307176; doi:10.1186/s12977-014-0133-5)
Supplement: Additional file 5: — Annotation Clusters and Enrichment data for HIV-1 infected MDM using DAVID. [file 12977_2014_133_MOESM5_ESM.pdf]

Additional file 5. Annotation Clusters and Enrichment data for HIV-1 infected MDM using DAVID

Cluster and Category	Enrichment and Term	Count	%	P value	Genes	Fold Enrichment
Annotation Cluster 1	***Enrichment Score: 11.065950515302008***					
GOTERM_BP_FAT	GO:0006414~translational elongation	27	5.152672	3.27E-16	P46776, P62750, P29692, P62753, P27635, P08865, P84098, P61247, P62241, P47914, P61313, P63220, P23396, P62263, P46782, P62701, P46783, Q02543, P49411, P05388, P62913, P25398, P26640, P50914, P40429, P39023	7.77719578
SP_PIR_KEYWORDS	ribosome	20	3.816794	5.31E-14	P46783, Q02543, P05388, P62753, P62913, P27635, P08865, P25398, P84098, P61247, P62241, P47914, P63220, P23396, P62263, P40429, P46782, P62701, P39023	10.0569905
GOTERM_CC_FAT	GO:0022626~cytosolic ribosome	22	4.198473	3.52E-13	P46783, Q02543, P46776, P62750, P05388, P62753, P62913, P27635, P08865, P25398, P84098, P61247, P62241, P50914, P47914, P63220, P23396, P62263, P46782, P62701, P39023	7.71478738
SP_PIR_KEYWORDS	ribosomal protein	26	4.961832	5.81E-11	Q71UM5, P46776, P62750, P27635, P62753, P08865, P84098, P61247, P62241, P47914, P61313, P63220, P23396, P62263, P46782, P62701, P46783, Q02543, P05388, P62913, P25398, Q92552, P50914, P40429, P39023	5.07664041
GOTERM_CC_FAT	GO:0033279~ribosomal subunit	24	4.580153	9.23E-11	P46783, Q02543, P46776, P62750, P05388, P62753, P27635, P62913, P08865, P25398, P06748, P84098, P61247, P62241, P50914, P47914, P63220, P23396, P62263, P40429, P46782, P62701, P39023	5.32583333
KEGG_PATHWAY	hsa03010:Ribosome	23	4.389313	1.72E-10	Q71UM5, P46783, Q02543, P46776, P62750, P05388, P62753, P62913, P27635, P08865, P25398, P84098, P61247, P62241, P50914, P47914, P61313, P63220, P23396, P40429, P46782, P62701, P39023	5.1704244
GOTERM_CC_FAT	GO:0005840~ribosome	29	5.534351	2.02E-09	Q71UM5, P46776, P62750, P62753, P27635, P08865, P84098, P61247, P62241, P47914, P61313, P63220, P23396, P62263, P46782, P62701, P46783, Q02543, P05388, P62913, P25398, P06748, Q9P2E9, Q92552, P50914, O76021, P40429, P39023	3.83129716
GOTERM_MF_FAT	GO:0003735~structural constituent of ribosome	25	4.770992	2.62E-09	Q71UM5, Q02543, P46776, P62750, P05388, P62753, P27635, P62913, P25398, P08865, P84098, P61247, P62241, P50914, P47914, O76021, P61313, P63220, P62263, P23396, P40429, P46782, P39023, P62701	4.35133795
Annotation Cluster 2	***Enrichment Score: 8.588842774814447***					
GOTERM_BP_FAT	GO:0008104~protein localization	67	12.78626	1.28E-09	Q96QK1, Q12907, O75351, P21757, P31946, O60493, P15311, P61970, P51572, Q7Z3B4, O43707, O15127, P51571, P42566, O00161, Q16543, O00186, Q9Y490, Q8WUM4, Q96AH8, P62913, Q9Y5X3, O75369, P35579, P06748, P04233, Q9P2E9, P62258, Q07812, Q9BY43, O00159, Q10567, P31150, P55957, Q96L92, Q96CW1, O00170, Q13596, P60953, P61026, Q96FZ7, Q03518, Q9UEU0, Q9H269, P61981, P50897, O94973, Q9BZG1, Q15907, P20340, P55072, Q15843, Q5JWF2, P30101, Q12846, Q9BVL2, Q9NP72, Q13636, P51148, O75396, P20339, P50395, P35241, Q9UBQ0, O95721, Q14764, P53367	2.20997245
GOTERM_BP_FAT	GO:0015031~protein transport	60	11.45038	3.08E-09	Q96QK1, Q12907, O75351, P21757, P31946, O60493, P61970, P51572, Q7Z3B4, O43707, P51571, O15127, P42566, O00161, Q16543, O00186, Q8WUM4, Q96AH8, P62913, Q9Y5X3, P35579, P06748, P04233, Q9P2E9, P62258, Q9BY43, O00159, Q10567, P31150, P55957, Q96L92, Q96CW1, O00170, Q13596, P61026, Q96FZ7, Q03518, Q9UEU0, P61981, Q9H269, P50897, O94973, Q9BZG1, P20340, Q15907, P55072, Q5JWF2, P30101, Q12846, Q9BVL2, Q9NP72, Q13636, P51148, O75396, P20339, P50395, Q9UBQ0, O95721, Q14764, P53367	2.29074591
GOTERM_BP_FAT	GO:0045184~establishment of protein localization	60	11.45038	4.33E-09	Q96QK1, Q12907, O75351, P21757, P31946, O60493, P61970, P51572, Q7Z3B4, O43707, P51571, O15127, P42566, O00161, Q16543, O00186, Q8WUM4, Q96AH8, P62913, Q9Y5X3, P35579, P06748, P04233, Q9P2E9, P62258, Q9BY43, O00159, Q10567, P31150, P55957, Q96L92, Q96CW1, O00170, Q13596, P61026, Q96FZ7, Q03518, Q9UEU0, P61981, Q9H269, P50897, O94973, Q9BZG1, P20340, Q15907, P55072, Q5JWF2, P30101, Q12846, Q9BVL2, Q9NP72, Q13636, P51148, O75396, P20339, P50395, Q9UBQ0, O95721, Q14764, P53367	2.26989387
Annotation Cluster 3	***Enrichment Score: 7.703728491545319***					
GOTERM_BP_FAT	GO:0046365~monosaccharide catabolic process	18	3.435115	1.77E-10	P37837, P40925, P40926, P18669, P00338, P04075, P06733, P10515, P07195, P46926, P11177, P52790, O95336, P00558, P52789, P52209, P14618, Q9UKK9	7.37555657
GOTERM_BP_FAT	GO:0046164~alcohol catabolic process	19	3.625954	1.93E-10	P40925, P37837, P40926, P18669, P00338, P04075, P06733, P10515, P21964, P07195, P46926, P11177, P52790, O95336, P00558, P52789, P52209, P14618, Q9UKK9	6.82416036
GOTERM_BP_FAT	GO:0006007~glucose catabolic process	16	3.053435	6.56E-10	P37837, P40925, P40926, P18669, P04075, P06733, P10515, P00338, P07195, P11177, P52790, O95336, P00558, P52789, P52209, P14618	8.02550983
GOTERM_BP_FAT	GO:0016052~carbohydrate catabolic process	21	4.007634	7.22E-10	P40925, P37837, P40926, P15586, P18669, P00338, P04075, P06733, P10515, P07195, P46926, P11177, P52790, O95336, P00558, P52789, P08236, P52209, P14550, P14618, Q9UKK9	5.60497189

GOTERM_BP_FAT	GO:0044275~cellular carbohydrate catabolic process	18	3.435115	3.49E-09	P37837, P40925, P40926, P18669, P00338, P04075, P06733, P10515, P07195, P46926, P11177, P52790, O95336, P00558, P52789, P52209, P14618, Q9UKK9	6.16075901
GOTERM_BP_FAT	GO:0019320~hexose catabolic process	16	3.053435	8.83E-09	P37837, P40925, P40926, P18669, P04075, P06733, P10515, P00338, P07195, P11177, P52790, O95336, P00558, P52789, P52209, P14618	6.74608072
GOTERM_BP_FAT	GO:0006096~glycolysis	13	2.480916	4.03E-08	P40925, P40926, P18669, P04075, P06733, P10515, P00338, P07195, P11177, P52790, P00558, P52789, P14618	8.04685427
GOTERM_BP_FAT	GO:0005996~monosaccharide metabolic process	24	4.580153	2.35E-06	Q06210, P40925, P37837, P40926, P18669, P00338, P04075, P06733, P10515, Q96C23, P07195, P46926, P11177, P52790, O95336, P00558, P52789, Q9UJ70, P50416, P52209, P14550, P14618, Q9UKK9, P51570	3.14513223
GOTERM_BP_FAT	GO:0019318~hexose metabolic process	21	4.007634	9.86E-06	Q06210, P40925, P37837, P40926, P18669, P00338, P04075, P06733, P10515, Q96C23, P07195, P11177, P52790, O95336, P00558, P52789, P50416, P52209, P14550, P14618, P51570	3.18198925
GOTERM_BP_FAT	GO:0006006~glucose metabolic process	18	3.435115	1.97E-05	P37837, P40925, P40926, P18669, P00338, P04075, P06733, P10515, P07195, P11177, P52790, O95336, P00558, P52789, P50416, P52209, P14550, P14618	3.4226439
Annotation Cluster 4	***Enrichment Score: 6.462972304113294***					
GOTERM_CC_FAT	GO:0031982~vesicle	53	10.1145	5.5E-08	P31946, P23526, Q9H4M9, P62993, Q9UHL4, O43707, P05023, Q06830, Q07866, O00161, P31146, O00186, P02787, P02786, Q8WUM4, Q9BTU6, P11279, P62258, Q9BY43, P10909, Q10567, P98082, Q96CW1, P27105, P02751, P52566, P04075, P30086, P09622, P30041, P07900, P50897, O94973, P08195, Q15907, Q9P0L0, Q5JWF2, P30101, Q12846, P02768, P43007, P19878, P07858, P49327, Q9NZM1, P18031, P51148, Q15084, O75396, P20339, P61421, P57737, P20073	2.24691874
GOTERM_CC_FAT	GO:0031988~membrane-bounded vesicle	46	8.778626	2.63E-07	P27105, P02751, P52566, P04075, P30086, P31946, P09622, P23526, Q9H4M9, P30041, P07900, P50897, P62993, O94973, P08195, Q9UHL4, Q15907, O43707, P05023, Q06830, O00161, P30101, P31146, O00186, Q12846, P02787, P02786, P02768, P43007, P19878, Q8WUM4, P07858, Q9BTU6, P49327, P11279, P51148, Q15084, O75396, P20339, P61421, P62258, P57737, Q9BY43, P10909, P98082, Q96CW1	2.30035994
GOTERM_CC_FAT	GO:0016023~cytoplasmic membrane-bounded vesicle	44	8.396947	7.13E-07	P27105, P02751, P52566, P30086, P31946, P09622, P23526, Q9H4M9, P30041, P07900, P50897, O94973, P08195, Q9UHL4, Q15907, O43707, P05023, Q06830, O00161, P30101, P31146, O00186, Q12846, P02787, P02768, P02786, P43007, P19878, Q8WUM4, P07858, Q9BTU6, P49327, P11279, P51148, Q15084, O75396, P20339, P61421, P62258, Q9BY43, P57737, P10909, P98082, Q96CW1	2.27235556
GOTERM_CC_FAT	GO:0031410~cytoplasmic vesicle	48	9.160305	1.36E-06	P27105, P02751, P52566, P30086, P31946, P09622, P23526, Q9H4M9, P30041, P07900, P50897, O94973, P08195, Q9UHL4, Q15907, O43707, P05023, Q06830, Q07866, O00161, P30101, P31146, O00186, Q12846, P02787, P02768, P02786, P43007, P19878, Q8WUM4, P07858, Q9BTU6, P49327, P11279, Q9NZM1, P18031, P51148, Q15084, O75396, P20339, P61421, P62258, P57737, Q9BY43, P10909, Q10567, P98082, Q96CW1	2.12369678
Annotation Cluster 5	***Enrichment Score: 5.316735319725446***					
SP_PIR_KEYWORDS	lysosome	18	3.435115	6.54E-07	Q9UHG3, P15586, P07858, P04062, Q96AH8, P11279, Q9H269, P30041, P04233, P50897, Q9NUN5, P08236, P34810, Q9UHL4, O15118, P08962, P53634, Q14108	4.43452533
GOTERM_CC_FAT	GO:0005773~vacuole	26	4.961832	2.86E-06	P15586, O75351, Q9UEU0, P09622, Q9H269, P30041, Q9ULA0, P50897, P45974, Q9UHL4, P08962, O00161, Q12846, Q9UHG3, P04062, P07858, Q96AH8, P11279, P04233, P61421, Q9NUN5, P08236, P34810, O15118, P53634, Q14108	2.93061728
GOTERM_CC_FAT	GO:0005764~lysosome	22	4.198473	1.7E-05	O00161, Q9UHG3, P15586, O75351, P07858, P04062, Q96AH8, P11279, Q9UEU0, Q9H269, P30041, P04233, P50897, Q9NUN5, P45974, P08236, P34810, Q9UHL4, O15118, P08962, P53634, Q14108	2.96160084
GOTERM_CC_FAT	GO:0000323~lytic vacuole	22	4.198473	1.7E-05	O00161, Q9UHG3, P15586, O75351, P07858, P04062, Q96AH8, P11279, Q9UEU0, Q9H269, P30041, P04233, P50897, Q9NUN5, P45974, P08236, P34810, Q9UHL4, O15118, P08962, P53634, Q14108	2.96160084
Annotation Cluster 6	***Enrichment Score: 5.284887143704187***					
SP_PIR_KEYWORDS	nucleotide-binding	88	16.79389	3.72E-09	Q9Y5M8, Q96T60, O75351, P47897, P31153, Q9BZE4, P68371, Q9H4M9, P61081, Q13283, P38606, P52789, Q9UJ70, P53396, P05023, P25098, P51570, Q9Y285, O00160, P49588, P49411, P17987, P22314, P41252, Q9BTU6, Q96AH8, P17844, P35579, O94804, P00558, Q13418, Q96CM8, O00159, O60313, P61158, O60488, P35998, P27105, P34932, P40227, Q9Y6G9, P60953, P14868, P61026, P30520, P36776, P30086, Q03518, P33121, Q99832, Q92841, P78527, P61221, Q9H223, P61163, P33176, P07900, P61160, P08107, P20591, P48643, Q9BZG1, P40616, P68032, P53602, P50990, Q15907, P20340, P55072, Q5JWF2, Q9Y5P6, O75643, Q9NP72, P50148, P20020, Q13636, P51148, P61086, P33897, P26640, P49368, P20339, P06576, P52790, Q14204, P25705, P10301, P14618, Q9H7F0	1.91595809

GOTERM_MF_FAT	GO:0017076~purine nucleotide binding	101	19.27481	5.51E-06	Q9Y5M8, O75351, P31153, P61081, Q13283, P38606, Q9UJ70, P52789, P53396, O00160, P49411, P49588, P17987, P41252, P17844, P00558, O95881, Q96CM8, O00159, O60313, O60488, Q9BRQ8, Q15067, P35998, P34932, Q9Y6G9, P40227, P60953, P61026, P14598, P09622, P33121, Q99832, P61221, Q9H223, P31040, P20591, Q9BZG1, P40616, P68032, P53602, P50990, Q15907, P55072, Q5JWF2, Q9Y5P6, Q9NP72, P20020, Q13636, P61086, P33897, P06576, Q96HE7, Q6IBS0, P10301, P14618, Q9H7F0, Q96T60, P47897, Q9BZE4, P68371, Q9H4M9, P61604, Q658P3, P13804, P05023, P25098, P51570, Q9Y285, P22314, Q9BTU6, Q96AH8, P35579, O94804, Q13418, P61158, P27105, P14868, P30520, P36776, P30086, Q03518, Q92841, P78527, P61163, P33176, P07900, P08107, P61160, P48643, P20340, O75643, P00390, P50148, P51148, P26640, P49368, P20339, P52790, Q14204, P25705	1.53980192
GOTERM_MF_FAT	GO:0032555~purine ribonucleotide binding	91	17.36641	0.000188	Q9Y5M8, Q96T60, O75351, P47897, P31153, Q9BZE4, P68371, Q9H4M9, P61604, P61081, Q13283, P38606, P52789, Q9UJ70, P53396, P05023, P25098, P51570, Q9Y285, O00160, P49588, P49411, P17987, P22314, P41252, Q9BTU6, Q96AH8, P17844, P35579, O94804, P00558, Q13418, Q96CM8, O00159, O60313, P61158, O60488, P35998, P27105, P34932, P40227, Q9Y6G9, P60953, P14868, P61026, P30520, P36776, P30086, P14598, Q03518, P33121, Q99832, Q92841, P78527, P61221, Q9H223, P61163, P33176, P07900, P61160, P08107, P20591, P48643, Q9BZG1, P40616, P68032, P53602, P50990, Q15907, P20340, P55072, Q5JWF2, Q9Y5P6, O75643, Q9NP72, P50148, P20020, Q13636, P51148, P61086, P26640, P33897, P49368, P20339, P06576, P52790, Q14204, Q6IBS0, P25705, P10301, P14618, Q9H7F0	1.44930838
GOTERM_MF_FAT	GO:0032553~ribonucleotide binding	91	17.36641	1.88E-04	Q9Y5M8, Q96T60, O75351, P47897, P31153, Q9BZE4, P68371, Q9H4M9, P61604, P61081, Q13283, P38606, P52789, Q9UJ70, P53396, P05023, P25098, P51570, Q9Y285, O00160, P49588, P49411, P17987, P22314, P41252, Q9BTU6, Q96AH8, P17844, P35579, O94804, P00558, Q13418, Q96CM8, O00159, O60313, P61158, O60488, P35998, P27105, P34932, P40227, Q9Y6G9, P60953, P14868, P61026, P30520, P36776, P30086, P14598, Q03518, P33121, Q99832, Q92841, P78527, P61221, Q9H223, P61163, P33176, P07900, P61160, P08107, P20591, P48643, Q9BZG1, P40616, P68032, P53602, P50990, Q15907, P20340, P55072, Q5JWF2, Q9Y5P6, O75643, Q9NP72, P50148, P20020, Q13636, P51148, P61086, P26640, P33897, P49368, P20339, P06576, P52790, Q14204, Q6IBS0, P25705, P10301, P14618, Q9H7F0	1.44930838
Annotation Cluster 7	***Enrichment Score: 4.637050016070767***					
INTERPRO	IPR002194:Chaperonin TCP-1, conserved site	6	1.145038	5.8E-06	P49368, P40227, P17987, P48643, P50990, Q99832	19.5988235
INTERPRO	IPR017998:Chaperone, tailless complex polypeptide 1	6	1.145038	1.04E-05	P49368, P40227, P17987, P48643, P50990, Q99832	17.8171123
PIR_SUPERFAMILY	PIRSF002584:molecular chaperone t-complex-type	6	1.145038	3.70E-05	P49368, P40227, P17987, P48643, P50990, Q99832	13.3261261
GOTERM_CC_FAT	GO:0005832~chaperonin-containing T-complex	5	0.954198	4.83E-05	P49368, P40227, P17987, P48643, Q99832	20.2888889
INTERPRO	IPR002423:Chaperonin Cpn60/TCP-1	6	1.145038	6.08E-05	P49368, P40227, P17987, P48643, P50990, Q99832	13.0658824
Annotation Cluster 8	***Enrichment Score: 4.326145700108451***					
SP_PIR_KEYWORDS	mrna splicing	22	4.198473	2.85E-07	P51991, O75643, P49756, P62306, P17844, Q9Y333, P23246, O95232, P62304, Q07955, Q15427, Q96PU8, P11940, P07910, Q9H2H8, P26599, Q9UHX1, P67809, P62318, O75934, P09651, O60506	3.86400161
SP_PIR_KEYWORDS	mrna processing	22	4.198473	9.54E-06	P51991, O75643, P49756, P62306, P17844, Q9Y333, P23246, O95232, P62304, Q07955, Q15427, Q96PU8, P11940, P07910, Q9H2H8, P26599, Q9UHX1, P67809, P62318, O75934, P09651, O60506	3.10606283
GOTERM_BP_FAT	GO:0008380~RNA splicing	24	4.580153	0.000122	O14744, P51991, O75643, P49756, P62306, P17844, Q9Y333, P23246, O95232, P62304, Q07955, Q15427, Q96PU8, P11940, P07910, Q9H2H8, P26599, Q9UHX1, P67809, P62318, O75934, Q14103, P09651, O60506	2.45851886
GOTERM_BP_FAT	GO:0006397~mRNA processing	24	4.580153	0.000714	O14744, P51991, O75643, P49756, P62306, P17844, Q9Y333, P23246, O95232, P62304, Q07955, Q15427, Q96PU8, P11940, P07910, Q9H2H8, P26599, Q9UHX1, P67809, P62318, O75934, Q14103, P09651, O60506	2.17513818
GOTERM_BP_FAT	GO:0016071~mRNA metabolic process	26	4.961832	0.000993	O14744, P62306, P23246, Q9Y333, P62304, Q15427, P08107, Q9UHX1, P13489, O75934, P09651, O60506, P51991, O75643, P49756, P17844, O95232, Q07955, P11940, Q96PU8, Q9H2H8, P07910, P26599, P67809, P62318, Q14103	2.04433595
Annotation Cluster 9	***Enrichment Score: 3.8716596170633593***					

GOTERM_BP_FAT	GO:0046496~nicotinamide nucleotide metabolic process	9	1.717557	5.27E-05	P37837, P40925, P40926, O95336, O75874, P52209, Q16719, P07195, P36957	6.54580645
GOTERM_BP_FAT	GO:0006769~nicotinamide metabolic process	9	1.717557	5.27E-05	P37837, P40925, P40926, O95336, O75874, P52209, Q16719, P07195, P36957	6.54580645
GOTERM_BP_FAT	GO:0009820~alkaloid metabolic process	9	1.717557	6.36E-05	P37837, P40925, P40926, O95336, O75874, P52209, Q16719, P07195, P36957	6.38615264
GOTERM_BP_FAT	GO:0019362~pyridine nucleotide metabolic process	9	1.717557	7.62E-05	P37837, P40925, P40926, O95336, O75874, P52209, Q16719, P07195, P36957	6.23410138
GOTERM_BP_FAT	GO:0043603~cellular amide metabolic process	10	1.908397	1.10E-04	P37837, P40925, P40926, O95336, O75874, P52209, Q16719, P07195, P36957, P09622	5.19508449
GOTERM_BP_FAT	GO:0006733~oxidoreduction coenzyme metabolic process	9	1.717557	0.00036	P37837, P40925, P40926, O95336, O75874, P52209, Q16719, P07195, P36957	5.03523573
GOTERM_BP_FAT	GO:0019748~secondary metabolic process	10	1.908397	0.001488	P37837, P40925, P40926, O95336, O75874, P52209, Q16719, P07195, P36957, P11766	3.68259153
Annotation Cluster 10	***Enrichment Score: 3.755057031401147***					
GOTERM_BP_FAT	GO:0051187~cofactor catabolic process	8	1.526718	6.86E-05	P31040, P40925, P11177, P40926, O75874, P09601, P36957, O75390	7.507735
GOTERM_BP_FAT	GO:0046356~acetyl-CoA catabolic process	7	1.335878	9.68E-05	P31040, P40925, P11177, P40926, O75874, P36957, O75390	8.85423095
GOTERM_BP_FAT	GO:0006099~tricarboxylic acid cycle	7	1.335878	9.68E-05	P31040, P40925, P11177, P40926, O75874, P36957, O75390	8.85423095
GOTERM_BP_FAT	GO:0009109~coenzyme catabolic process	7	1.335878	0.000202	P31040, P40925, P11177, P40926, O75874, P36957, O75390	7.83258892
SP_PIR_KEYWORDS	tricarboxylic acid cycle	6	1.145038	0.000207	P31040, P40925, P40926, O75874, P36957, O75390	10.4880044
GOTERM_BP_FAT	GO:0009060~aerobic respiration	7	1.335878	0.001098	P31040, P40925, P11177, P40926, O75874, P36957, O75390	5.81849462
Annotation Cluster 11	***Enrichment Score: 3.6108107996745735***					
SMART	SM00360:RRM	17	3.244275	6.07E-05	O14979, P51991, P49756, Q01844, P23246, Q07955, Q15427, Q15056, Q15020, P11940, P07910, P26599, Q9UHX1, Q13283, Q14103, P09651, O60506	3.26555095
INTERPRO	IPR012677:Nucleotide-binding, alpha-beta plait	18	3.435115	0.000305	O14979, P62750, P51991, P49756, Q01844, P23246, Q07955, Q15427, Q15056, Q15020, P11940, P07910, P26599, Q9UHX1, Q13283, Q14103, P09651, O60506	2.76039768
INTERPRO	IPR000504:RNA recognition motif, RNP-1	17	3.244275	0.000795	O14979, P51991, P49756, Q01844, P23246, Q07955, Q15427, Q15056, Q15020, P11940, P07910, P26599, Q9UHX1, Q13283, Q14103, P09651, O60506	2.63175355
Annotation Cluster 12	***Enrichment Score: 3.315666361084132***					
GOTERM_BP_FAT	GO:0030036~actin cytoskeleton organization	21	4.007634	0.000103	P31146, P60953, Q9Y490, P52566, P04075, P43034, Q9Y6W5, P23528, O75369, P35579, P60981, O43491, P07737, P52907, P15311, P50552, Q27J81, P68032, O43707, O95466, P13796	2.7032829
GOTERM_BP_FAT	GO:0030029~actin filament-based process	21	4.007634	0.000245	P31146, P60953, Q9Y490, P52566, P04075, P43034, Q9Y6W5, P23528, O75369, P35579, P60981, O43491, P07737, P52907, P15311, P50552, Q27J81, P68032, O43707, O95466, P13796	2.53502878
GOTERM_BP_FAT	GO:0007010~cytoskeleton organization	27	5.152672	0.004481	Q8TDZ2, P60953, P52566, P04075, P35527, P43034, Q9Y6W5, P43487, P23528, O43491, P07737, P52907, P15311, P68032, O43707, P31146, Q9Y490, Q13561, O75369, P35579, P06748, P60981, P50552, Q27J81, Q14204, O95466, P13796	1.80159811
Annotation Cluster 13	***Enrichment Score: 3.2902141314156435***					
SP_PIR_KEYWORDS	atp-binding	66	12.59542	2.67E-06	Q96T60, O75351, P31153, P47897, Q9H4M9, P61081, Q13283, P38606, Q9UJ70, P52789, P53396, P05023, P25098, P51570, Q9Y285, O00160, P49588, P17987, P22314, P41252, Q9BTU6, P17844, P35579, O94804, P00558, Q13418, Q96CM8, O00159, P61158, O60488, P35998, P27105, P34932, P40227, Q9Y6G9, P14868, P36776, P30086, Q03518, P33121, P78527, Q92841, Q99832, P61221, P61163, Q9H223, P33176, P07900, P08107, P61160, P48643, P53602, P68032, P50990, P55072, O75643, P20020, P61086, P33897, P26640, P49368, P06576, P52790, Q14204, P25705, P14618, Q9H7F0	1.82709578

GOTERM_MF_FAT	GO:0030554~adenyl nucleotide binding	79	15.07634	0.000417	Q96T60, O75351, P47897, P31153, Q9H4M9, P61604, P61081, Q13283, Q658P3, P38606, P52789, Q9UJ70, P13804, P53396, P05023, P25098, P51570, Q9Y285, O00160, P49588, P17987, P22314, P41252, Q9BTU6, P17844, P35579, O94804, P00558, O95881, Q13418, Q96CM8, O00159, P61158, O60488, Q9BRQ8, Q15067, P35998, P27105, P34932, P40227, Q9Y6G9, P14868, P36776, P30086, Q03518, P09622, P33121, Q92841, P78527, Q99832, P61221, Q9H223, P61163, P31040, P33176, P07900, P61160, P08107, P48643, P68032, P53602, P50990, P55072, O75643, P00390, Q9NP72, P20020, P61086, P33897, P26640, P49368, P06576, P52790, Q14204, Q96HE7, Q6IBS0, P25705, P14618, Q9H7F0	1.46483087
GOTERM_MF_FAT	GO:0001882~nucleoside binding	80	15.26718	0.000507	Q96T60, O75351, P47897, P31153, Q9H4M9, P61604, P61081, Q13283, Q658P3, P38606, P52789, Q9UJ70, P13804, P53396, O43707, P05023, P25098, P51570, Q9Y285, O00160, P49588, P17987, P22314, P41252, Q9BTU6, P17844, P35579, O94804, P00558, O95881, Q13418, Q96CM8, O00159, P61158, O60488, Q9BRQ8, Q15067, P35998, P27105, P34932, P40227, Q9Y6G9, P14868, P36776, P30086, Q03518, P09622, P33121, Q92841, P78527, Q99832, P61221, Q9H223, P61163, P31040, P33176, P07900, P61160, P08107, P48643, P68032, P53602, P50990, P55072, O75643, P00390, Q9NP72, P20020, P61086, P33897, P26640, P49368, P06576, P52790, Q14204, Q96HE7, Q6IBS0, P25705, P14618, Q9H7F0	1.45116581
GOTERM_MF_FAT	GO:0001883~purine nucleoside binding	79	15.07634	0.000653	Q96T60, O75351, P47897, P31153, Q9H4M9, P61604, P61081, Q13283, Q658P3, P38606, P52789, Q9UJ70, P13804, P53396, P05023, P25098, P51570, Q9Y285, O00160, P49588, P17987, P22314, P41252, Q9BTU6, P17844, P35579, O94804, P00558, O95881, Q13418, Q96CM8, O00159, P61158, O60488, Q9BRQ8, Q15067, P35998, P27105, P34932, P40227, Q9Y6G9, P14868, P36776, P30086, Q03518, P09622, P33121, Q92841, P78527, Q99832, P61221, Q9H223, P61163, P31040, P33176, P07900, P61160, P08107, P48643, P68032, P53602, P50990, P55072, O75643, P00390, Q9NP72, P20020, P61086, P33897, P26640, P49368, P06576, P52790, Q14204, Q96HE7, Q6IBS0, P25705, P14618, Q9H7F0	1.44287214
GOTERM_MF_FAT	GO:0005524~ATP binding	69	13.16794	0.006052	Q96T60, O75351, P31153, P47897, Q9H4M9, P61604, P61081, Q13283, P38606, Q9UJ70, P52789, P53396, P05023, P25098, P51570, Q9Y285, O00160, P49588, P17987, P22314, P41252, Q9BTU6, P17844, P35579, O94804, P00558, Q13418, Q96CM8, O00159, P61158, O60488, P35998, P27105, P34932, P40227, Q9Y6G9, P14868, P36776, P30086, Q03518, P33121, P78527, Q92841, Q99832, P61221, Q9H223, P61163, P33176, P07900, P61160, P08107, P48643, P68032, P53602, P50990, P55072, O75643, Q9NP72, P20020, P61086, P33897, P26640, P49368, P06576, P52790, Q14204, Q6IBS0, P25705, P14618, Q9H7F0	1.3660314
GOTERM_MF_FAT	GO:0032559~adenyl ribonucleotide binding	69	13.16794	0.008139	Q96T60, O75351, P31153, P47897, Q9H4M9, P61604, P61081, Q13283, P38606, Q9UJ70, P52789, P53396, P05023, P25098, P51570, Q9Y285, O00160, P49588, P17987, P22314, P41252, Q9BTU6, P17844, P35579, O94804, P00558, Q13418, Q96CM8, O00159, P61158, O60488, P35998, P27105, P34932, P40227, Q9Y6G9, P14868, P36776, P30086, Q03518, P33121, P78527, Q92841, Q99832, P61221, Q9H223, P61163, P33176, P07900, P61160, P08107, P48643, P68032, P53602, P50990, P55072, O75643, Q9NP72, P20020, P61086, P33897, P26640, P49368, P06576, P52790, Q14204, Q6IBS0, P25705, P14618, Q9H7F0	1.34778115
Annotation Cluster 14	***Enrichment Score: 3.1800267356290535***					
SMART	SM00244:PHB	5	0.954198	0.000213	P27105, Q9UJZ1, O75477, P30086, Q99623	15.5889423
INTERPRO	IPR001107:Band 7 protein	5	0.954198	0.000494	P27105, Q9UJZ1, O75477, P30086, Q99623	12.5633484
PIR_SUPERFAMILY	PIRSF005651:membrane protease subunits, stomatin/prohibitin homologs	4	0.763359	0.002741	P27105, Q9UJZ1, P30086, Q99623	12.6915487
Annotation Cluster 15	***Enrichment Score: 3.1557913617308255***					
GOTERM_MF_FAT	GO:0003924~GTPase activity	20	3.816794	0.000113	Q5JWF2, P60953, P49411, P50148, Q9NP72, P14598, Q13636, Q9BZE4, Q9H4M9, P68371, P51148, Q9H223, P20339, P20591, P40616, Q9BZG1, P10301, P20340, Q15907, O60313	2.77165791
UP_SEQ_FEATURE	nucleotide phosphate-binding region:GTP	21	4.007634	0.000194	Q5JWF2, Q9Y5M8, P60953, P49411, P61026, P30520, P50148, Q96AH8, Q9NP72, Q13636, Q9BZE4, P68371, P51148, P20339, P20591, P40616, Q9BZG1, P10301, P20340, Q15907, O60313	2.59653901
SP_PIR_KEYWORDS	gtp-binding	22	4.198473	0.000281	Q5JWF2, Q9Y5M8, Q9Y5P6, P60953, P49411, P61026, P30520, P50148, Q96AH8, Q9NP72, Q13636, Q9BZE4, P68371, P51148, P20339, P20591, P40616, Q9BZG1, P10301, P20340, Q15907, O60313	2.45463932
GOTERM_MF_FAT	GO:0005525~GTP binding	25	4.770992	0.002143	Q9Y5M8, P60953, P61026, P30520, P14598, Q9BZE4, Q9H4M9, P68371, Q9H223, P20591, Q9BZG1, P40616, Q15907, P20340, Q5JWF2, Q9Y5P6, P49411, P50148, Q9NP72, Q96AH8, Q13636, P51148, P20339, P10301, O60313	1.96512036
GOTERM_MF_FAT	GO:0032561~guanyl ribonucleotide binding	25	4.770992	0.002971	Q9Y5M8, P60953, P61026, P30520, P14598, Q9BZE4, Q9H4M9, P68371, Q9H223, P20591, Q9BZG1, P40616, Q15907, P20340, Q5JWF2, Q9Y5P6, P49411, P50148, Q9NP72, Q96AH8, Q13636, P51148, P20339, P10301, O60313	1.91367742

GOTERM_MF_FAT	GO:0019001~guanyl nucleotide binding	25	4.770992	0.002971	Q9Y5M8, P60953, P61026, P30520, P14598, Q9BZE4, Q9H4M9, P68371, Q9H223, P20591, Q9BZG1, P40616, Q15907, P20340, Q5JWF2, Q9Y5P6, P49411, P50148, Q9NP72, Q96AH8, Q13636, P51148, P20339, P10301, O60313	1.91367742
Annotation Cluster 16	***Enrichment Score: 3.1021412765058383***					
GOTERM_BP_FAT	GO:0000375~RNA splicing, via transesterification reactions	15	2.862595	0.00079	O14744, P51991, O75643, P49756, P62306, Q9Y333, P62304, Q07955, Q15427, P07910, P26599, P67809, P62318, Q14103, P09651	2.85220325
GOTERM_BP_FAT	GO:0000377~RNA splicing, via transesterification reactions with bulged adenosine as nucleophile	15	2.862595	0.00079	O14744, P51991, O75643, P49756, P62306, Q9Y333, P62304, Q07955, Q15427, P07910, P26599, P67809, P62318, Q14103, P09651	2.85220325
GOTERM_BP_FAT	GO:0000398~nuclear mRNA splicing, via spliceosome	15	2.862595	0.00079	O14744, P51991, O75643, P49756, P62306, Q9Y333, P62304, Q07955, Q15427, P07910, P26599, P67809, P62318, Q14103, P09651	2.85220325
Annotation Cluster 17	***Enrichment Score: 3.025874501682239***					
GOTERM_BP_FAT	GO:0042981~regulation of apoptosis	46	8.778626	0.000802	Q71UM5, P42224, P52565, P14174, P62753, P31946, P09601, P23528, P78527, P68371, P61604, P50897, P08107, P30044, Q9H3N1, P20591, P61247, Q9NR28, O43707, P55072, P23396, Q06830, P04080, P30101, P04083, P49588, P02768, P08575, P07858, P62913, P06748, O95865, Q92835, P04233, P09211, P09525, Q07812, P62258, O95881, Q13418, P10909, P55957, Q9NQC3, P16070, Q9BRQ8	1.66449473
GOTERM_BP_FAT	GO:0043067~regulation of programmed cell death	46	8.778626	0.000986	Q71UM5, P42224, P52565, P14174, P62753, P31946, P09601, P23528, P78527, P68371, P61604, P50897, P08107, P30044, Q9H3N1, P20591, P61247, Q9NR28, O43707, P55072, P23396, Q06830, P04080, P30101, P04083, P49588, P02768, P08575, P07858, P62913, P06748, O95865, Q92835, P04233, P09211, P09525, Q07812, P62258, O95881, Q13418, P10909, P55957, Q9NQC3, P16070, Q9BRQ8	1.64809577
GOTERM_BP_FAT	GO:0010941~regulation of cell death	46	8.778626	0.001057	Q71UM5, P42224, P52565, P14174, P62753, P31946, P09601, P23528, P78527, P68371, P61604, P50897, P08107, P30044, Q9H3N1, P20591, P61247, Q9NR28, O43707, P55072, P23396, Q06830, P04080, P30101, P04083, P49588, P02768, P08575, P07858, P62913, P06748, O95865, Q92835, P04233, P09211, P09525, Q07812, P62258, O95881, Q13418, P10909, P55957, Q9NQC3, P16070, Q9BRQ8	1.64202916
Annotation Cluster 18	***Enrichment Score: 3.021625064457108***					
GOTERM_BP_FAT	GO:0006200~ATP catabolic process	6	1.145038	0.000148	P06576, O75351, P48047, P53396, P25705, P36776	10.9096774
GOTERM_BP_FAT	GO:0009261~ribonucleotide catabolic process	7	1.335878	0.00016	P06576, O75351, P48047, P53396, P25705, P36776, Q9UKK9	8.14589247
GOTERM_BP_FAT	GO:0009203~ribonucleoside triphosphate catabolic process	6	1.145038	0.000363	P06576, O75351, P48047, P53396, P25705, P36776	9.18709677
GOTERM_BP_FAT	GO:0009207~purine ribonucleoside triphosphate catabolic process	6	1.145038	0.000363	P06576, O75351, P48047, P53396, P25705, P36776	9.18709677
GOTERM_BP_FAT	GO:0009146~purine nucleoside triphosphate catabolic process	6	1.145038	0.000599	P06576, O75351, P48047, P53396, P25705, P36776	8.31213518
GOTERM_BP_FAT	GO:0009154~purine ribonucleotide catabolic process	6	1.145038	0.000937	P06576, O75351, P48047, P53396, P25705, P36776	7.58934081
GOTERM_BP_FAT	GO:0009143~nucleoside triphosphate catabolic process	6	1.145038	0.00115	P06576, O75351, P48047, P53396, P25705, P36776	7.27311828
GOTERM_BP_FAT	GO:0006195~purine nucleotide catabolic process	6	1.145038	0.004985	P06576, O75351, P48047, P53396, P25705, P36776	5.28954057
GOTERM_BP_FAT	GO:0009166~nucleotide catabolic process	7	1.335878	0.007686	P06576, O75351, P48047, P53396, P25705, P36776, Q9UKK9	3.99308455
GOTERM_BP_FAT	GO:0044270~nitrogen compound catabolic process	8	1.526718	0.007872	P06576, O75351, P48047, P53396, P25705, P36776, P09601, Q9UKK9	3.47372813
Annotation Cluster 19	***Enrichment Score: 2.9010352325845883***					
SMART	SM00175:RAB	9	1.717557	0.000112	P20339, P61026, Q9BZG1, Q9NP72, Q96AH8, P20340, Q15907, Q13636, P51148	5.98002049

UP_SEQ_FEATURE	short sequence motif:Effector region	11	2.099237	0.000272	P20339, P60953, P61026, Q9BZG1, Q9NP72, P10301, Q96AH8, P20340, Q15907, Q13636, P51148	4.22344315
UP_SEQ_FEATURE	lipid moiety-binding region:S-geranylgeranyl cysteine	11	2.099237	0.000484	P20339, P60953, P61026, Q9BZG1, Q9NP72, P10301, Q96AH8, P20340, Q15907, Q13636, P51148	3.93359901
INTERPRO	IPR003579:Ras small GTPase, Rab type	9	1.717557	0.00051	P20339, P61026, Q9BZG1, Q9NP72, Q96AH8, P20340, Q15907, Q13636, P51148	4.81938284
SP_PIR_KEYWORDS	prenylation	12	2.290076	0.003648	P02545, P20339, P60953, P61026, Q9BZG1, Q9NP72, P10301, Q96AH8, P20340, Q15907, Q13636, P51148	2.82369348
INTERPRO	IPR013753:Ras	11	2.099237	0.005012	P20339, P60953, P61026, Q9BZG1, Q9NP72, P10301, Q96AH8, P20340, Q15907, Q13636, P51148	2.87449412
INTERPRO	IPR005225:Small GTP-binding protein	13	2.480916	0.00529	P60953, P61026, Q9NP72, Q96AH8, Q9BZE4, Q13636, P51148, P20339, Q9BZG1, P40616, P10301, Q15907, P20340	2.54276154
INTERPRO	IPR001806:Ras GTPase	11	2.099237	0.008512	P20339, P60953, P61026, Q9BZG1, Q9NP72, P10301, Q96AH8, P20340, Q15907, Q13636, P51148	2.66156863
Annotation Cluster 20	***Enrichment Score: 2.76274651331982***					
GOTERM_BP_FAT	GO:0009259~ribonucleotide metabolic process	16	3.053435	0.000159	O75351, P04075, P30520, P36776, Q13488, P20020, P31939, P06576, P61421, P38606, P48047, P25705, P53396, Q9H7F0, P05023, Q9UKK9	3.16652769
GOTERM_BP_FAT	GO:0046034~ATP metabolic process	13	2.480916	0.000249	O75351, P04075, P36776, Q13488, P20020, P06576, P61421, P38606, P48047, P53396, P25705, P05023, Q9H7F0	3.60192524
GOTERM_BP_FAT	GO:0009150~purine ribonucleotide metabolic process	15	2.862595	0.000277	O75351, P04075, P30520, P36776, Q13488, P20020, P31939, P06576, P61421, P38606, P48047, P53396, P25705, Q9H7F0, P05023	3.16222534
GOTERM_BP_FAT	GO:0009205~purine ribonucleoside triphosphate metabolic process	13	2.480916	0.000675	O75351, P04075, P36776, Q13488, P20020, P06576, P61421, P38606, P48047, P53396, P25705, P05023, Q9H7F0	3.23249701
GOTERM_BP_FAT	GO:0009199~ribonucleoside triphosphate metabolic process	13	2.480916	0.000729	O75351, P04075, P36776, Q13488, P20020, P06576, P61421, P38606, P48047, P53396, P25705, P05023, Q9H7F0	3.20510297
GOTERM_BP_FAT	GO:0009144~purine nucleoside triphosphate metabolic process	13	2.480916	0.00098	O75351, P04075, P36776, Q13488, P20020, P06576, P61421, P38606, P48047, P53396, P25705, P05023, Q9H7F0	3.10001763
GOTERM_BP_FAT	GO:0009141~nucleoside triphosphate metabolic process	13	2.480916	0.001817	O75351, P04075, P36776, Q13488, P20020, P06576, P61421, P38606, P48047, P53396, P25705, P05023, Q9H7F0	2.88703932
GOTERM_BP_FAT	GO:0006163~purine nucleotide metabolic process	16	3.053435	0.001886	O75351, P04075, P30520, P36776, Q13488, P20020, P31939, P06576, P00492, P61421, P38606, P48047, P25705, P53396, Q9H7F0, P05023	2.50257833
GOTERM_BP_FAT	GO:0009152~purine ribonucleotide biosynthetic process	12	2.290076	0.002263	P06576, P61421, P38606, P48047, P30520, P04075, P25705, Q13488, P31939, P20020, Q9H7F0, P05023	2.9838434
GOTERM_BP_FAT	GO:0006754~ATP biosynthetic process	10	1.908397	0.003404	P06576, P61421, P38606, P48047, P04075, P25705, Q13488, P20020, Q9H7F0, P05023	3.2688172
GOTERM_BP_FAT	GO:0009260~ribonucleotide biosynthetic process	12	2.290076	0.003565	P06576, P61421, P38606, P48047, P30520, P04075, P25705, Q13488, P31939, P20020, Q9H7F0, P05023	2.81540062
GOTERM_BP_FAT	GO:0006164~purine nucleotide biosynthetic process	13	2.480916	0.004965	P04075, P30520, Q13488, P20020, P31939, P00492, P06576, P61421, P38606, P48047, P25705, P05023, Q9H7F0	2.55541994
GOTERM_BP_FAT	GO:0009206~purine ribonucleoside triphosphate biosynthetic process	10	1.908397	0.006438	P06576, P61421, P38606, P48047, P04075, P25705, Q13488, P20020, Q9H7F0, P05023	2.96861971
GOTERM_BP_FAT	GO:0009145~purine nucleoside triphosphate biosynthetic process	10	1.908397	0.006874	P06576, P61421, P38606, P48047, P04075, P25705, Q13488, P20020, Q9H7F0, P05023	2.93863365
GOTERM_BP_FAT	GO:0009201~ribonucleoside triphosphate biosynthetic process	10	1.908397	0.006874	P06576, P61421, P38606, P48047, P04075, P25705, Q13488, P20020, Q9H7F0, P05023	2.93863365
GOTERM_BP_FAT	GO:0009142~nucleoside triphosphate biosynthetic process	10	1.908397	0.008317	P06576, P61421, P38606, P48047, P04075, P25705, Q13488, P20020, Q9H7F0, P05023	2.85220325
Annotation Cluster 21	***Enrichment Score: 2.688331704112668***					
INTERPRO	IPR001557:L-lactate/malate dehydrogenase	4	0.763359	0.001416	P40925, P40926, P00338, P07195	16.3323529

INTERPRO	IPR001236:Lactate/malate dehydrogenase	4	0.763359	0.001416	P40925, P40926, P00338, P07195	16.3323529
INTERPRO	IPR015955:Lactate dehydrogenase/glycoside hydrolase, family 4, C-terminal	4	0.763359	0.002076	P40925, P40926, P00338, P07195	14.5176471
PIR_SUPERFAMILY	PIRSF000102:Lac_mal_DH	4	0.763359	0.004241	P40925, P40926, P00338, P07195	11.1051051
Annotation Cluster 22	***Enrichment Score: 2.6872232530691824***					
GOTERM_MF_FAT	GO:0042626~ATPase activity, coupled to transmembrane movement of substances	12	2.290076	0.001223	P33897, P06576, Q9UHG3, P61421, P38606, P48047, P25705, Q03518, P20020, Q9H7F0, P05023, O15533	3.21919167
GOTERM_MF_FAT	GO:0043492~ATPase activity, coupled to movement of substances	12	2.290076	0.001318	P33897, P06576, Q9UHG3, P61421, P38606, P48047, P25705, Q03518, P20020, Q9H7F0, P05023, O15533	3.18992629
GOTERM_MF_FAT	GO:0016820~hydrolase activity, acting on acid anhydrides, catalyzing transmembrane movement of substances	12	2.290076	0.001419	P33897, P06576, Q9UHG3, P61421, P38606, P48047, P25705, Q03518, P20020, Q9H7F0, P05023, O15533	3.16118822
GOTERM_MF_FAT	GO:0015399~primary active transmembrane transporter activity	12	2.290076	0.003015	P33897, P06576, Q9UHG3, P61421, P38606, P48047, P25705, Q03518, P20020, Q9H7F0, P05023, O15533	2.87616305
GOTERM_MF_FAT	GO:0015405~P-P-bond-hydrolysis-driven transmembrane transporter activity	12	2.290076	0.003015	P33897, P06576, Q9UHG3, P61421, P38606, P48047, P25705, Q03518, P20020, Q9H7F0, P05023, O15533	2.87616305
GOTERM_MF_FAT	GO:0042625~ATPase activity, coupled to transmembrane movement of ions	9	1.717557	0.003621	P06576, Q9UHG3, P61421, P38606, P48047, P25705, P20020, Q9H7F0, P05023	3.55633674
Annotation Cluster 23	***Enrichment Score: 2.581561636875662***					
GOTERM_BP_FAT	GO:0006085~acetyl-CoA biosynthetic process	4	0.763359	0.000742	P11177, P10515, P36957, P09622	19.3949821
GOTERM_BP_FAT	GO:0006086~acetyl-CoA biosynthetic process from pyruvate	3	0.572519	0.003442	P11177, P10515, P09622	29.0924731
GOTERM_CC_FAT	GO:0045254~pyruvate dehydrogenase complex	3	0.572519	0.007048	P11177, P10515, P09622	21.3033333
Annotation Cluster 24	***Enrichment Score: 2.56799221931492***					
GOTERM_CC_FAT	GO:0005741~mitochondrial outer membrane	11	2.099237	0.0012	P27105, Q07812, P52789, P50416, P30086, P55957, O60313, P33121, O60488, Q9BRQ8, P00387, P21796	3.47165432
GOTERM_CC_FAT	GO:0031968~organelle outer membrane	11	2.099237	0.003546	P27105, Q07812, P52789, P50416, P30086, P55957, O60313, P33121, O60488, Q9BRQ8, P00387, P21796	3.00431624
GOTERM_CC_FAT	GO:0019867~outer membrane	11	2.099237	0.004648	P27105, Q07812, P52789, P50416, P30086, P55957, O60313, P33121, O60488, Q9BRQ8, P00387, P21796	2.89304527
Annotation Cluster 25	***Enrichment Score: 2.1390104037218753***					
SP_PIR_KEYWORDS	proteasome	7	1.335878	0.004032	P35998, P25788, P28072, Q06323, P49721, O00233, P28066	4.58850191
GOTERM_BP_FAT	GO:0031396~regulation of protein ubiquitination	10	1.908397	0.007331	P35998, P25788, P28072, P63208, P62942, Q06323, P49721, Q9BZE4, O00233, P28066	2.90924731
GOTERM_BP_FAT	GO:0031398~positive regulation of protein ubiquitination	9	1.717557	0.008053	P35998, P25788, P28072, P63208, P62942, Q06323, P49721, O00233, P28066	3.11705069
GOTERM_BP_FAT	GO:0051437~positive regulation of ubiquitin-protein ligase activity during mitotic cell cycle	8	1.526718	0.008524	P35998, P25788, P28072, P63208, Q06323, P49721, O00233, P28066	3.4226439
GOTERM_BP_FAT	GO:0051443~positive regulation of ubiquitin-protein ligase activity	8	1.526718	0.009945	P35998, P25788, P28072, P63208, Q06323, P49721, O00233, P28066	3.32485407

Annotation Cluster 26	***Enrichment Score: 2.0573312624977116***					
INTERPRO	IPR004100:ATPase, F1/V1/A1 complex, alpha/beta subunit, N-terminal	3	0.572519	0.008763	P06576, P38606, P25705	19.5988235
INTERPRO	IPR000793:ATPase, F1/V1/A1 complex, alpha/beta subunit, C-terminal	3	0.572519	0.008763	P06576, P38606, P25705	19.5988235
INTERPRO	IPR000194:ATPase, F1/V1/A1 complex, alpha/beta subunit, nucleotide-binding domain	3	0.572519	0.008763	P06576, P38606, P25705	19.5988235
