# Supplementary material for: Opposing regulation of endolysosomal pathways by long-acting nanoformulated antiretroviral therapy and HIV-1 in human macrophages
Source: Retrovirology. 2015 Jan 22;12:5. doi: 10.1186/s12977-014-0133-5 (PMC4307176; doi:10.1186/s12977-014-0133-5)
Supplement: Additional file 6: — Annotation Clusters and Enrichment data for HIV-1 infected MDM treated with nanoATV using DAVID. [file 12977_2014_133_MOESM6_ESM.pdf]

Additional file 6. Annotation Clusters and Enrichment data for HIV-1 infected MDM treated with nanoATV using DAVID

Cluster and Category	Enrichment and Term	Count	%	P value	Genes	Fold Enrichment
Annotation Cluster 1	***Enrichment Score: 12.441499659441538***					
GOTERM_BP_FAT	GO:0006414~translational elongation	26	6.933333	1.02E-18	P36578, P46776, Q07020, P29692, P08865, P61353, P30050, Q9UNX3, P18621, P62424, P62269, P61247, P61313, P63220, P46781, P62701, P15880, P46783, P39019, P62081, P62987, P26641, P62861, P13639, P24534	10.5528953
SP_PIR_KEYWORDS	ribosome	19	5.066667	2.17E-15	P15880, P36578, P46783, P39019, P62081, Q07020, P08865, P61353, P30050, P62987, P62861, P18621, P62424, P62269, P61247, P63220, P46781, P62701	13.3503196
SP_PIR_KEYWORDS	ribonucleoprotein	31	8.266667	3.15E-14	P36578, P46776, P14866, Q07020, P08865, P61353, Q9Y333, P30050, Q9UNX3, P18621, P62424, P62269, P61247, P61313, P82921, P63220, P46781, P62701, P15880, P46783, P51991, P39019, P62081, P22087, P62987, P62861, Q92552, P82650, Q14103, Q15366	5.69925926
SP_PIR_KEYWORDS	ribosomal protein	25	6.666667	2.96E-13	P15880, P46783, P36578, P46776, P39019, P62081, Q07020, P61353, P08865, P30050, P62987, P18621, Q9UNX3, P62861, Q92552, P62424, P62269, P61247, P82650, P63220, P82921, P61313, P62701, P46781	6.82092199
GOTERM_CC_FAT	GO:0033279~ribosomal subunit	22	5.866667	1.41E-11	P15880, P36578, P46783, P46776, P39019, P62081, Q07020, P08865, P62987, P62861, P18621, Q9UNX3, P62424, P62269, P61247, P82650, P02792, P82921, P63220, P46781, P62701	6.57756362
KEGG_PATHWAY	hsa03010:Ribosome	21	5.6	2.27E-11	P15880, P36578, P46783, P46776, P39019, P62081, Q07020, P08865, P61353, P30050, P62987, P62861, P18621, Q9UNX3, P62424, P62269, P61247, P61313, P63220, P46781, P62701	6.49425287
GOTERM_MF_FAT	GO:0003735~structural constituent of ribosome	23	6.133333	1.25E-10	P15880, P36578, P46776, P39019, P62081, Q07020, P08865, P61353, P30050, P62987, P62861, P18621, Q9UNX3, P62424, P62269, P61247, P82650, P61313, P82921, P63220, P46781, P62701	5.58941674
GOTERM_CC_FAT	GO:0005840~ribosome	26	6.933333	3.55E-10	P36578, P46776, Q07020, P08865, P61353, P30050, Q9UNX3, P18621, P62424, P62269, P61247, P61313, P82921, P63220, P46781, P62701, P15880, P46783, P39019, P62081, P62987, P62861, Q92552, P02792, P82650	4.62793483
Annotation Cluster 2	***Enrichment Score: 7.125697894370129***					
GOTERM_CC_FAT	GO:0031988~membrane-bounded vesicle	40	10.66667	2.87E-08	P35606, P30086, P07686, Q14643, P23526, Q93050, P07900, P50897, P62993, P08195, Q92542, P01009, P50991, Q00610, P05023, O43747, Q06830, P08754, O00161, P30101, O00186, P02787, P05556, P02786, P02768, P49755, Q9BTU6, P49327, P11279, P51148, P62258, P10809, Q15363, Q86Y82, P07237, P16671, O43759, P16333, Q14956, P02647	2.69503247
GOTERM_CC_FAT	GO:0031982~vesicle	44	11.73333	3.73E-08	P49768, P35606, P30086, P07686, Q14643, P23526, Q93050, P07900, P50897, P62993, P08195, Q92542, P01009, P50991, Q00610, P05023, O43747, Q06830, P08754, Q9P0L0, O00161, P30101, O00186, P02787, P05556, P02786, P02768, P49755, Q9BTU6, P49327, P11279, Q9NZM1, P51148, P62258, P10809, Q15363, Q86Y82, P07237, P16671, O43759, Q10567, P16333, Q14956, P02647	2.51321834
GOTERM_CC_FAT	GO:0016023~cytoplasmic membrane-bounded vesicle	38	10.13333	1.14E-07	P35606, P30086, P07686, Q14643, P23526, Q93050, P07900, P50897, P08195, Q92542, P01009, P50991, Q00610, P05023, O43747, Q06830, P08754, O00161, P30101, O00186, P02787, P05556, P02786, P02768, P49755, Q9BTU6, P49327, P11279, P51148, P10809, P62258, Q15363, P07237, Q86Y82, P16671, O43759, Q14956, P02647	2.64407186
GOTERM_CC_FAT	GO:0031410~cytoplasmic vesicle	41	10.93333	2.58E-07	P49768, P35606, P30086, P07686, Q14643, P23526, Q93050, P07900, P50897, P08195, Q92542, P01009, P50991, Q00610, P05023, O43747, Q06830, P08754, O00161, P30101, O00186, P02787, P05556, P02786, P02768, P49755, Q9BTU6, P49327, P11279, Q9NZM1, P51148, P62258, P10809, Q15363, P07237, Q86Y82, P16671, O43759, Q10567, Q14956, P02647	2.44399985
Annotation Cluster 3	***Enrichment Score: 6.269407667375663***					
GOTERM_CC_FAT	GO:0019866~organelle inner membrane	28	7.466667	1.35E-07	P24752, Q9NS69, P49768, P53597, O95202, P10620, O75439, P30405, O00483, Q14643, P30049, P50440, P22695, P35914, Q99623, Q9Y4W6, P21912, P43003, O15229, Q9Y2Q3, P10809, P04179, P50416, O43169, P40939, Q8NC56, O60313, P56537	3.25697541
GOTERM_CC_FAT	GO:0044429~mitochondrial part	39	10.4	2.83E-07	P24752, O75431, Q9NS69, P49768, P53597, P45880, O95202, P10620, P30086, P30405, O75439, O00483, P33121, P30049, P50440, P82921, P22695, Q99623, P35914, Q9Y4W6, P21912, O94925, P43003, Q9Y2Q3, O15229, Q04837, P11177, P10809, O43169, P04179, P50416, P30084, P40939, P82650, P30038, P50213, P55957, O60313, O75390	2.50841846
GOTERM_CC_FAT	GO:0031966~mitochondrial membrane	30	8	4.65E-07	P24752, O75431, Q9NS69, P49768, P53597, P45880, O95202, P10620, P30086, O75439, P30405, O00483, P33121, P30049, P50440, P22695, P35914, Q99623, Q9Y4W6, P21912, P43003, Q9Y2Q3, O15229, P10809, P04179, P50416, O43169, P40939, P55957, O60313	2.91391836

GOTERM_CC_FAT	GO:0005743~mitochondrial inner membrane	25	6.666667	1.55E-06	P24752, Q9NS69, P49768, P53597, P10620, O95202, O75439, P30405, O00483, P30049, P50440, P22695, P35914, Q99623, Q9Y4W6, P21912, P43003, O15229, Q9Y2Q3, P10809, P04179, P50416, O43169, P40939, O60313	3.12658996
GOTERM_CC_FAT	GO:0005740~mitochondrial envelope	30	8	1.63E-06	P24752, O75431, Q9NS69, P49768, P53597, P45880, O95202, P10620, P30086, O75439, P30405, O00483, P33121, P30049, P50440, P22695, P35914, Q99623, Q9Y4W6, P21912, P43003, Q9Y2Q3, O15229, P10809, P04179, P50416, O43169, P40939, P55957, O60313	2.74005688
Annotation Cluster 4	***Enrichment Score: 6.184276222537203***					
GOTERM_BP_FAT	GO:0008104~protein localization	48	12.8	2.86E-07	O75431, Q13596, Q14974, P60953, Q9NS69, P61026, P49768, P27797, P35606, Q96FZ7, Q9UEU0, O75436, Q9H269, P50897, P15311, P62328, Q9BZG1, Q15904, P20340, Q00610, P55072, O43747, Q15843, O00161, P30101, Q16543, O00186, P46459, P49755, Q96AH8, P21333, Q13636, O75369, O60749, P51148, P50395, P04233, P62258, P60468, Q15363, Q86Y82, P16671, O43759, Q10567, O95721, P31150, P55957, O00410	2.23096269
GOTERM_BP_FAT	GO:0045184~establishment of protein localization	43	11.46667	6.99E-07	O75431, Q13596, Q14974, Q9NS69, P61026, P49768, P27797, P35606, Q96FZ7, Q9UEU0, O75436, Q9H269, P50897, Q9BZG1, Q15904, P20340, Q00610, P55072, O43747, O00161, P30101, Q16543, O00186, P46459, P49755, Q96AH8, P21333, Q13636, O60749, P51148, P50395, P04233, P60468, P62258, Q15363, Q86Y82, P16671, O43759, Q10567, O95721, P31150, P55957, O00410	2.29224889
GOTERM_BP_FAT	GO:0015031~protein transport	42	11.2	1.4E-06	O75431, Q13596, Q14974, Q9NS69, P61026, P49768, P27797, P35606, Q96FZ7, Q9UEU0, O75436, Q9H269, P50897, Q9BZG1, Q15904, P20340, Q00610, P55072, O43747, O00161, P30101, Q16543, O00186, P46459, P49755, Q96AH8, Q13636, O60749, P51148, P50395, P04233, P60468, P62258, Q15363, Q86Y82, P16671, O43759, Q10567, O95721, P31150, P55957, O00410	2.25950847
Annotation Cluster 5	***Enrichment Score: 5.455635307824368***					
GOTERM_CC_FAT	GO:0000267~cell fraction	57	15.2	4.66E-07	O15427, P08648, P10620, P30405, P43034, P07686, P17858, Q9H4A4, P62424, Q9UJ70, P05023, P25098, O00161, Q9Y285, P40925, P05556, P49588, P49755, Q9BTU6, P54578, P11279, Q9BRK5, P04179, Q15363, Q13724, P07237, Q9Y6A9, P31150, P55957, P49407, Q9NS69, Q15836, P49768, P27797, P62942, P30086, Q14643, P33121, P50897, O00754, Q92542, P55072, P08754, Q9P0L0, P15090, P00390, P50148, P43003, Q9BQB6, P07099, P50416, O43169, P63261, P34810, P16671, O95721, P30519	2.01418216
GOTERM_CC_FAT	GO:0005624~membrane fraction	44	11.73333	6.10E-06	O15427, P08648, Q9NS69, Q15836, P49768, P27797, P62942, P10620, P30086, P43034, P30405, P33121, Q14643, P50897, P62424, Q92542, P05023, P55072, P25098, P08754, Q9P0L0, O00161, P05556, P49755, Q9BTU6, P50148, P43003, P54578, Q9BQB6, P11279, P07099, O43169, P50416, Q15363, Q13724, P07237, P34810, P16671, O95721, Q9Y6A9, P31150, P55957, P30519, P49407	2.08140456
GOTERM_CC_FAT	GO:0005626~insoluble fraction	44	11.73333	1.51E-05	O15427, P08648, Q9NS69, Q15836, P49768, P27797, P62942, P10620, P30086, P43034, P30405, P33121, Q14643, P50897, P62424, Q92542, P05023, P55072, P25098, P08754, Q9P0L0, O00161, P05556, P49755, Q9BTU6, P50148, P43003, P54578, Q9BQB6, P11279, P07099, O43169, P50416, Q15363, Q13724, P07237, P34810, P16671, O95721, Q9Y6A9, P31150, P55957, P30519, P49407	2.00698008
Annotation Cluster 6	***Enrichment Score: 5.002499093194939***					
SP_PIR_KEYWORDS	gtp-binding	22	5.866667	2.09E-06	P08754, P07437, Q9Y5P6, P60953, Q9NVJ2, P61026, P53597, P50148, Q96AH8, Q13636, Q9BZE4, P68371, Q6DD88, P51148, P11233, P13639, Q9BZG1, P20340, O60313, Q9BUF5, P41091, Q16181	3.42994934
UP_SEQ_FEATURE	nucleotide phosphate-binding region:GTP	20	5.333333	6.18E-06	P08754, P07437, P60953, P61026, Q9NVJ2, P50148, Q96AH8, Q13636, Q9BZE4, P68371, Q6DD88, P51148, P11233, P13639, Q9BZG1, P20340, O60313, Q9BUF5, P41091, Q16181	3.45545763
GOTERM_MF_FAT	GO:0005525~GTP binding	25	6.666667	1.48E-05	P07437, Q8NF50, P60953, P61026, P53597, Q9BZE4, P68371, Q6DD88, Q9H223, P11233, Q9BZG1, P20340, Q9BUF5, P08754, Q9Y5P6, Q9NVJ2, P50148, Q96AH8, Q13636, P51148, P21980, P13639, O60313, Q16181, P41091	2.74375296
GOTERM_MF_FAT	GO:0019001~guanyl nucleotide binding	25	6.666667	2.25E-05	P07437, Q8NF50, P60953, P61026, P53597, Q9BZE4, P68371, Q6DD88, Q9H223, P11233, Q9BZG1, P20340, Q9BUF5, P08754, Q9Y5P6, Q9NVJ2, P50148, Q96AH8, Q13636, P51148, P21980, P13639, O60313, Q16181, P41091	2.67192696
GOTERM_MF_FAT	GO:0032561~guanyl ribonucleotide binding	25	6.666667	2.25E-05	P07437, Q8NF50, P60953, P61026, P53597, Q9BZE4, P68371, Q6DD88, Q9H223, P11233, Q9BZG1, P20340, Q9BUF5, P08754, Q9Y5P6, Q9NVJ2, P50148, Q96AH8, Q13636, P51148, P21980, P13639, O60313, Q16181, P41091	2.67192696
Annotation Cluster 7	***Enrichment Score: 4.994891158658105***					
GOTERM_CC_FAT	GO:0005773~vacuole	21	5.6	9.81E-06	O00161, P49768, Q9NVJ2, P04062, Q96AH8, P11279, P13686, Q9UEU0, P07686, Q9H269, P01903, Q9ULA0, P04233, P50897, Q01459, O00754, Q92542, P34810, Q15904, P53634, P00813	3.18912176

GOTERM_CC_FAT	GO:0000323~lytic vacuole	19	5.066667	1.03E-05	O00161, P49768, Q9NVJ2, P04062, Q96AH8, P11279, P13686, Q9UEU0, P07686, Q9H269, P01903, P04233, P50897, Q01459, O00754, Q92542, P34810, P53634, P00813	3.44606522
GOTERM_CC_FAT	GO:0005764~lysosome	19	5.066667	1.03E-05	O00161, P49768, Q9NVJ2, P04062, Q96AH8, P11279, P13686, Q9UEU0, P07686, Q9H269, P01903, P04233, P50897, Q01459, O00754, Q92542, P34810, P53634, P00813	3.44606522
Annotation Cluster 8	***Enrichment Score: 4.847598121877984***					
GOTERM_BP_FAT	GO:0031400~negative regulation of protein modification process	14	3.733333	6.32E-06	P35998, P25788, P49768, P62942, P30086, Q9BZE4, P62987, P28072, P62258, Q9UNM6, P49721, O00233, O00232, P28066	4.8228164
GOTERM_BP_FAT	GO:0051248~negative regulation of protein metabolic process	17	4.533333	1.34E-05	P35998, P25788, P49768, P27797, P62942, P21333, P30086, P19838, Q9BZE4, P62987, P28072, P62258, Q9UNM6, P49721, O00233, O00232, P28066	3.72672176
GOTERM_BP_FAT	GO:0032269~negative regulation of cellular protein metabolic process	16	4.266667	3.39E-05	P35998, P25788, P49768, P27797, P62942, P30086, P19838, Q9BZE4, P62987, P28072, P62258, Q9UNM6, P49721, O00233, O00232, P28066	3.64390572
Annotation Cluster 9	***Enrichment Score: 4.537383915287949***					
GOTERM_BP_FAT	GO:0030029~actin filament-based process	21	5.6	1.8E-06	Q99439, P06702, P60953, P05556, P27797, P21333, O15511, P43034, Q9Y6W5, P23528, O75369, P60981, Q9Y613, Q9Y4I1, P11233, P15311, P50552, P47756, P62328, P63261, P16333	3.57208601
GOTERM_BP_FAT	GO:0030036~actin cytoskeleton organization	20	5.333333	2.7E-06	Q99439, P06702, P60953, P05556, P27797, P21333, O15511, P43034, Q9Y6W5, P23528, O75369, P60981, Q9Y613, P11233, P15311, P50552, P47756, P62328, P63261, P16333	3.62778225
GOTERM_BP_FAT	GO:0007010~cytoskeleton organization	21	5.6	0.005036	Q99439, P07437, P06702, P60953, P05556, P27797, P21333, O15511, P43034, Q9Y6W5, P23528, O75369, P60981, Q9Y613, P11233, P15311, P50552, P47756, P62328, P63261, P16333	1.97447873
Annotation Cluster 10	***Enrichment Score: 4.4535369975071735***					
GOTERM_BP_FAT	GO:0051187~cofactor catabolic process	8	2.133333	7.51E-06	P40925, P11177, P21912, P53597, P50213, P48735, O75390, P30519	10.5790811
GOTERM_BP_FAT	GO:0006099~tricarboxylic acid cycle	7	1.866667	1.41E-05	P40925, P11177, P21912, P53597, P50213, P48735, O75390	12.4764163
GOTERM_BP_FAT	GO:0046356~acetyl-CoA catabolic process	7	1.866667	1.41E-05	P40925, P11177, P21912, P53597, P50213, P48735, O75390	12.4764163
GOTERM_BP_FAT	GO:0009060~aerobic respiration	8	2.133333	1.77E-05	P40925, P11177, P21912, P53597, P50213, P48735, O75390, P22695	9.37004329
GOTERM_BP_FAT	GO:0009109~coenzyme catabolic process	7	1.866667	3.02E-05	P40925, P11177, P21912, P53597, P50213, P48735, O75390	11.0368298
SP_PIR_KEYWORDS	tricarboxylic acid cycle	6	1.6	4.26E-05	P40925, P21912, P53597, P50213, P48735, O75390	14.6552381
GOTERM_BP_FAT	GO:0006084~acetyl-CoA metabolic process	7	1.866667	8.71E-05	P40925, P11177, P21912, P53597, P50213, P48735, O75390	9.25669599
KEGG_PATHWAY	hsa00020:Citrate cycle (TCA cycle)	7	1.866667	8.02E-04	P40925, P11177, P21912, P53597, P50213, P48735, O75390	6.07526882
Annotation Cluster 11	***Enrichment Score: 4.382292157580419***					
INTERPRO	IPR017998:Chaperone, tailless complex polypeptide 1	6	1.6	2.04E-06	P10809, P17987, P48643, P50990, P50991, Q99832	24.8271237
INTERPRO	IPR002423:Chaperonin Cpn60/TCP-1	6	1.6	1.23E-05	P10809, P17987, P48643, P50990, P50991, Q99832	18.2065574
INTERPRO	IPR002194:Chaperonin TCP-1, conserved site	5	1.333333	4.29E-05	P17987, P48643, P50990, P50991, Q99832	22.7581967
PIR_SUPERFAMILY	PIRSF002584:molecular chaperone t-complex-type	5	1.333333	0.000201	P17987, P48643, P50990, P50991, Q99832	15.218107
GOTERM_CC_FAT	GO:0005832~chaperonin-containing T-complex	4	1.066667	0.000567	P17987, P48643, P50991, Q99832	21.8682635
Annotation Cluster 12	***Enrichment Score: 4.322011532423317***					
GOTERM_BP_FAT	GO:0043161~proteasomal ubiquitin-dependent protein catabolic process	13	3.466667	6.66E-06	P35998, P25788, P62987, P28072, Q8TAT6, Q9UNM6, P60468, P54725, P49721, P55072, O00233, O00232, P28066	5.22471777
GOTERM_BP_FAT	GO:0010498~proteasomal protein catabolic process	13	3.466667	6.66E-06	P35998, P25788, P62987, P28072, Q8TAT6, Q9UNM6, P60468, P54725, P49721, P55072, O00233, O00232, P28066	5.22471777

GOTERM_BP_FAT	GO:0006511~ubiquitin-dependent protein catabolic process	15	4	0.002437	Q15843, P35998, P25788, P54578, P62987, P28072, Q8TAT6, P60468, Q9UNM6, P54725, P49721, P55072, O00233, O00232, P28066	2.54094666
Annotation Cluster 13	***Enrichment Score: 4.0606055602156665***					
GOTERM_BP_FAT	GO:0034613~cellular protein localization	25	6.666667	6.88E-05	Q13596, O75431, Q14974, Q9NS69, P60953, P27797, P35606, Q9UEU0, Q9H269, Q15904, Q00610, P55072, O43747, Q16543, P30101, P21333, O60749, P04233, P60468, P62258, Q86Y82, Q10567, O43759, P55957, O00410	2.49354862
GOTERM_BP_FAT	GO:0070727~cellular macromolecule localization	25	6.666667	7.67E-05	Q13596, O75431, Q14974, Q9NS69, P60953, P27797, P35606, Q9UEU0, Q9H269, Q15904, Q00610, P55072, O43747, Q16543, P30101, P21333, O60749, P04233, P60468, P62258, Q86Y82, Q10567, O43759, P55957, O00410	2.47547943
GOTERM_BP_FAT	GO:0006886~intracellular protein transport	23	6.133333	0.000125	P30101, Q16543, O75431, Q13596, Q14974, Q9NS69, P27797, P35606, Q9UEU0, Q9H269, O60749, P04233, P60468, P62258, Q86Y82, Q15904, O43759, Q00610, Q10567, P55957, O43747, P55072, O00410	2.52101766
Annotation Cluster 14	***Enrichment Score: 3.99030146751753***					
GOTERM_CC_FAT	GO:0031968~organelle outer membrane	12	3.2	8.18E-05	O75431, Q9NS69, O43169, P49768, P50416, P45880, P10620, P30086, P55957, O60313, P33121, O15229	4.41570705
GOTERM_CC_FAT	GO:0005741~mitochondrial outer membrane	11	2.933333	0.000113	O75431, Q9NS69, O43169, P50416, P45880, P10620, P30086, P55957, O60313, P33121, O15229	4.67737858
GOTERM_CC_FAT	GO:0019867~outer membrane	12	3.2	0.000115	O75431, Q9NS69, O43169, P49768, P50416, P45880, P10620, P30086, P55957, O60313, P33121, O15229	4.25216234
Annotation Cluster 15	***Enrichment Score: 3.7018302564374146***					
GOTERM_CC_FAT	GO:0031974~membrane-enclosed lumen	75	20	8.75E-05	P24752, Q96T60, P53597, P30405, Q9Y230, P23246, P23528, Q9BZE4, Q09028, P61247, Q9HB71, P50440, P04080, P15880, P06702, Q68CZ2, P39019, Q8IYB3, P17844, Q6P179, P22087, Q9BRK5, P11177, O75629, Q04837, P10809, P04179, P07237, Q9BVC6, O60313, P02647, P19338, P56537, O00410, Q14974, O43670, Q7L014, P14866, P27797, P45880, P25685, O75439, Q14643, P78527, P48643, P01009, P53602, P30049, P82921, Q8NBJ5, P02652, P55072, P35914, P46781, P30101, P02768, P51991, O94925, P55265, P62081, P17096, P19838, Q9Y2Q3, Q13838, P62987, Q9NYU2, P30084, Q969G3, P40939, P82650, Q05519, P30038, P50213, Q9Y4L1, O75390	1.54644913
GOTERM_CC_FAT	GO:0043233~organelle lumen	72	19.2	0.000246	P24752, Q96T60, P53597, P30405, Q9Y230, P23246, P23528, Q9BZE4, Q09028, P61247, P04080, P15880, P06702, Q68CZ2, P39019, Q8IYB3, P17844, Q6P179, P22087, Q9BRK5, P11177, O75629, Q04837, P10809, P04179, P07237, Q9BVC6, P02647, P19338, P56537, O00410, Q14974, O43670, Q7L014, P14866, P27797, P45880, P25685, O75439, Q14643, P78527, P48643, P01009, P53602, P30049, P82921, Q8NBJ5, P02652, P55072, P35914, P46781, P30101, P02768, P51991, O94925, P55265, P62081, P17096, P19838, Q9Y2Q3, Q13838, P62987, Q9NYU2, P30084, Q969G3, P40939, P82650, Q05519, P30038, P50213, Q9Y4L1, O75390	1.5139567
GOTERM_CC_FAT	GO:0070013~intracellular organelle lumen	70	18.66667	0.000364	P24752, Q96T60, P53597, P30405, Q9Y230, P23246, P23528, Q9BZE4, Q09028, P61247, P04080, P15880, P06702, Q68CZ2, P39019, Q8IYB3, P17844, Q6P179, P22087, Q9BRK5, P11177, O75629, Q04837, P10809, P04179, P07237, Q9BVC6, P02647, P19338, P56537, O00410, Q14974, O43670, Q7L014, P14866, P45880, P27797, P25685, O75439, Q14643, P78527, P48643, P53602, P30049, P82921, Q8NBJ5, P02652, P55072, P35914, P46781, P30101, P51991, O94925, P55265, P62081, P17096, P19838, Q9Y2Q3, Q13838, P62987, Q9NYU2, P30084, Q969G3, P40939, P82650, Q05519, P30038, P50213, Q9Y4L1, O75390	1.50582478
Annotation Cluster 16	***Enrichment Score: 3.4721179406230864***					
SP_PIR_KEYWORDS	nucleotide-binding	61	16.26667	3.28E-06	P07437, Q96T60, P53597, Q9Y230, Q9BZE4, P68371, P17858, Q6DD88, Q9Y4I1, P11233, Q9UJ70, P05023, Q9BUF5, P25098, Q9Y285, Q9Y4W6, P49588, P46459, P17987, P22314, Q96AH8, Q9BTU6, P17844, P60842, P10809, O60313, Q16181, P35998, Q9NVI7, P60953, P61026, Q7L014, P45880, P30086, P33121, P78527, Q99832, Q9H223, P07900, P61160, P48643, Q9BZG1, P53602, Q15904, P50990, P50991, P20340, P55072, P08754, Q9Y5P6, Q9NVJ2, P50148, Q13636, Q12931, O43252, P51148, Q13838, P13639, P63261, Q9Y4L1, P41091	1.85580862
GOTERM_MF_FAT	GO:0017076~purine nucleotide binding	68	18.13333	0.001272	P07437, Q8NF50, Q96T60, P53597, Q9Y230, Q9BZE4, P68371, P17858, Q6DD88, Q9Y4I1, P11233, Q9UJ70, P05023, Q9BUF5, P25098, Q9Y285, Q9Y4W6, P49588, P46459, P17987, P22314, Q9BTU6, Q96AH8, P17844, O15229, P60842, P01903, P04839, P21980, P10809, O60313, Q16181, P35998, Q9NVI7, P60953, P61026, Q7L014, P30086, P33121, P78527, Q99832, Q9H223, P07900, P61160, P48643, Q9BZG1, P53602, P30049, P50990, Q15904, P50991, P20340, P55072, P08754, Q9Y5P6, P00390, Q9NVJ2, P50148, Q13636, Q12931, O43252, P51148, Q13838, P13639, P63261, Q6IBS0, Q9Y4L1, P41091	1.44746559

GOTERM_MF_FAT	GO:0032553~ribonucleotide binding	65	17.33333	0.001761	P07437, Q8NF50, Q96T60, P53597, Q9Y230, Q9BZE4, P68371, P17858, Q6DD88, Q9Y4I1, P11233, Q9UJ70, P05023, Q9BUF5, P25098, Q9Y285, Q9Y4W6, P49588, P46459, P17987, P22314, Q96AH8, Q9BTU6, P17844, P60842, P01903, P21980, P10809, O60313, Q16181, P35998, Q9NVI7, P60953, P61026, Q7L014, P30086, P33121, P78527, Q99832, Q9H223, P07900, P61160, P48643, Q9BZG1, P53602, P30049, P50990, Q15904, P50991, P20340, P55072, P08754, Q9Y5P6, Q9NVJ2, P50148, Q13636, Q12931, O43252, P51148, Q13838, P13639, P63261, Q6IBS0, Q9Y4L1, P41091	1.44540189
GOTERM_MF_FAT	GO:0032555~purine ribonucleotide binding	65	17.33333	0.001761	P07437, Q8NF50, Q96T60, P53597, Q9Y230, Q9BZE4, P68371, P17858, Q6DD88, Q9Y4I1, P11233, Q9UJ70, P05023, Q9BUF5, P25098, Q9Y285, Q9Y4W6, P49588, P46459, P17987, P22314, Q96AH8, Q9BTU6, P17844, P60842, P01903, P21980, P10809, O60313, Q16181, P35998, Q9NVI7, P60953, P61026, Q7L014, P30086, P33121, P78527, Q99832, Q9H223, P07900, P61160, P48643, Q9BZG1, P53602, P30049, P50990, Q15904, P50991, P20340, P55072, P08754, Q9Y5P6, Q9NVJ2, P50148, Q13636, Q12931, O43252, P51148, Q13838, P13639, P63261, Q6IBS0, Q9Y4L1, P41091	1.44540189
Annotation Cluster 17	***Enrichment Score: 3.4306457988146875***					
GOTERM_BP_FAT	GO:0031397~negative regulation of protein ubiquitination	10	2.666667	7.34E-05	P62987, P35998, P25788, P28072, Q9UNM6, P49721, Q9BZE4, O00233, O00232, P28066	5.53972154
SP_PIR_KEYWORDS	proteasome	8	2.133333	0.000101	P35998, P25788, P28072, Q9UNM6, P49721, O00233, O00232, P28066	7.32761905
GOTERM_BP_FAT	GO:0031396~regulation of protein ubiquitination	11	2.933333	0.000157	P62987, P35998, P25788, P28072, Q9UNM6, P62942, P49721, Q9BZE4, O00233, O00232, P28066	4.50933333
GOTERM_BP_FAT	GO:0051436~negative regulation of ubiquitin-protein ligase activity during mitotic cell cycle	9	2.4	0.00017	P62987, P35998, P25788, P28072, Q9UNM6, P49721, O00233, O00232, P28066	5.67608392
GOTERM_BP_FAT	GO:0031145~anaphase-promoting complex-dependent proteasomal ubiquitin-dependent protein catabolic process	9	2.4	0.00017	P62987, P35998, P25788, P28072, Q9UNM6, P49721, O00233, O00232, P28066	5.67608392
GOTERM_BP_FAT	GO:0031398~positive regulation of protein ubiquitination	10	2.666667	0.000198	P62987, P35998, P25788, P28072, Q9UNM6, P62942, P49721, O00233, O00232, P28066	4.88023088
GOTERM_BP_FAT	GO:0051352~negative regulation of ligase activity	9	2.4	0.00021	P62987, P35998, P25788, P28072, Q9UNM6, P49721, O00233, O00232, P28066	5.50664858
GOTERM_BP_FAT	GO:0051444~negative regulation of ubiquitin-protein ligase activity	9	2.4	0.00021	P62987, P35998, P25788, P28072, Q9UNM6, P49721, O00233, O00232, P28066	5.50664858
GOTERM_BP_FAT	GO:0051437~positive regulation of ubiquitin-protein ligase activity during mitotic cell cycle	9	2.4	0.000233	P62987, P35998, P25788, P28072, Q9UNM6, P49721, O00233, O00232, P28066	5.42566845
GOTERM_BP_FAT	GO:0051443~positive regulation of ubiquitin-protein ligase activity	9	2.4	0.000286	P62987, P35998, P25788, P28072, Q9UNM6, P49721, O00233, O00232, P28066	5.27064935
GOTERM_BP_FAT	GO:0051439~regulation of ubiquitin-protein ligase activity during mitotic cell cycle	9	2.4	0.000315	P62987, P35998, P25788, P28072, Q9UNM6, P49721, O00233, O00232, P28066	5.19641485
GOTERM_BP_FAT	GO:0051351~positive regulation of ligase activity	9	2.4	0.000382	P62987, P35998, P25788, P28072, Q9UNM6, P49721, O00233, O00232, P28066	5.05404732
GOTERM_BP_FAT	GO:0051438~regulation of ubiquitin-protein ligase activity	9	2.4	0.0006	P62987, P35998, P25788, P28072, Q9UNM6, P49721, O00233, O00232, P28066	4.73006993
GOTERM_BP_FAT	GO:0051340~regulation of ligase activity	9	2.4	0.000773	P62987, P35998, P25788, P28072, Q9UNM6, P49721, O00233, O00232, P28066	4.55488215
GOTERM_CC_FAT	GO:0000502~proteasome complex	8	2.133333	0.000997	P35998, P25788, P28072, Q9UNM6, P49721, O00233, O00232, P28066	5.01894572
GOTERM_BP_FAT	GO:0031401~positive regulation of protein modification process	12	3.2	0.006081	P62987, P35998, P25788, P28072, Q9UNM6, P49768, P62942, P02790, P49721, O00233, O00232, P28066	2.63062713
KEGG_PATHWAY	hsa03050:Proteasome	7	1.866667	0.007163	P35998, P25788, P28072, Q9UNM6, P49721, O00232, P28066	4.0070922
Annotation Cluster 18	***Enrichment Score: 3.1855183808271903***					

GOTERM_BP_FAT	GO:0043933~macromolecular complex subunit organization	33	8.8	0.000568	P07437, Q14974, Q9NS69, Q15836, P27797, P62942, P30086, P68371, P17858, Q6DD88, P07900, P62993, Q9BUF5, P55072, P02652, O00186, P17987, P21333, P17096, P60981, P04233, P21980, P10809, P16402, P04179, P68431, Q969G3, Q15393, P16333, P02647, P56537, Q16181, O00410	1.90535211
GOTERM_BP_FAT	GO:0006461~protein complex assembly	26	6.933333	0.000618	P07437, Q14974, Q9NS69, Q15836, P62942, P27797, P30086, P68371, P17858, Q6DD88, P07900, P62993, Q9BUF5, P55072, O00186, P17987, P21333, P17096, P04233, P21980, P10809, P04179, Q15393, P16333, O00410, Q16181	2.11057906
GOTERM_BP_FAT	GO:0070271~protein complex biogenesis	26	6.933333	0.000618	P07437, Q14974, Q9NS69, Q15836, P62942, P27797, P30086, P68371, P17858, Q6DD88, P07900, P62993, Q9BUF5, P55072, O00186, P17987, P21333, P17096, P04233, P21980, P10809, P04179, Q15393, P16333, O00410, Q16181	2.11057906
GOTERM_BP_FAT	GO:0065003~macromolecular complex assembly	31	8.266667	0.000836	P07437, Q14974, Q9NS69, Q15836, P62942, P27797, P30086, P68371, P17858, Q6DD88, P07900, P62993, Q9BUF5, P55072, P02652, O00186, P17987, P21333, P17096, P04233, P21980, P10809, P16402, P04179, P68431, Q15393, P16333, P02647, P56537, Q16181, O00410	1.91099567
Annotation Cluster 19	***Enrichment Score: 2.7633770165530978***					
GOTERM_BP_FAT	GO:0000375~RNA splicing, via transesterification reactions	12	3.2	0.001291	Q15427, P51991, P14866, Q8IYB3, Q05519, Q15393, P62995, Q14103, Q9Y333, Q15366, Q9BWJ5, Q13838	3.21521093
GOTERM_BP_FAT	GO:0000377~RNA splicing, via transesterification reactions with bulged adenosine as nucleophile	12	3.2	0.001291	Q15427, P51991, P14866, Q8IYB3, Q05519, Q15393, P62995, Q14103, Q9Y333, Q15366, Q9BWJ5, Q13838	3.21521093
GOTERM_BP_FAT	GO:0000398~nuclear mRNA splicing, via spliceosome	12	3.2	0.001291	Q15427, P51991, P14866, Q8IYB3, Q05519, Q15393, P62995, Q14103, Q9Y333, Q15366, Q9BWJ5, Q13838	3.21521093
GOTERM_BP_FAT	GO:0008380~RNA splicing	16	4.266667	0.004111	P51991, P14866, Q7L014, Q8IYB3, P17844, Q9Y333, P23246, Q9BWJ5, Q13838, Q15427, Q9H2H8, Q05519, Q15393, P62995, Q14103, Q15366	2.30951771
Annotation Cluster 20	***Enrichment Score: 2.7508129817461633***					
SMART	SM00102:ADF	4	1.066667	0.000506	Q6IBS0, P23528, P60981, Q9UJU6	23.7359477
INTERPRO	IPR002108:Actin-binding, cofilin/tropomyosin type	4	1.066667	0.001116	Q6IBS0, P23528, P60981, Q9UJU6	18.2065574
UP_SEQ_FEATURE	domain:ADF-H	3	0.8	0.00989	P23528, P60981, Q9UJU6	19.113
Annotation Cluster 21	***Enrichment Score: 2.6192911550025637***					
SP_PIR_KEYWORDS	proteinase	5	1.333333	0.000274	P25788, P28072, P49721, P22695, P28066	15.0862745
SP_PIR_KEYWORDS	threonine protease	4	1.066667	0.006502	P25788, P28072, P49721, P28066	10.2586667
INTERPRO	IPR001353:Proteasome, subunit alpha/beta	4	1.066667	0.007789	P25788, P28072, P49721, P28066	9.58239862
Annotation Cluster 22	***Enrichment Score: 2.618193945393547***					
SP_PIR_KEYWORDS	mrna splicing	13	3.466667	0.000836	P51991, Q7L014, Q8IYB3, P17844, Q9Y333, P23246, Q9BWJ5, Q13838, Q15427, Q9H2H8, Q05519, Q15393, P62995	3.19049442
SP_PIR_KEYWORDS	mrna processing	14	3.733333	0.001818	P51991, Q7L014, Q8IYB3, P55265, P17844, Q9Y333, P23246, Q9BWJ5, Q13838, Q15427, Q9H2H8, Q05519, Q15393, P62995	2.76194872
GOTERM_BP_FAT	GO:0008380~RNA splicing	16	4.266667	0.004111	P51991, P14866, Q7L014, Q8IYB3, P17844, Q9Y333, P23246, Q9BWJ5, Q13838, Q15427, Q9H2H8, Q05519, Q15393, P62995, Q14103, Q15366	2.30951771
GOTERM_BP_FAT	GO:0006397~mRNA processing	17	4.533333	0.00539	P51991, P14866, Q7L014, Q8IYB3, P55265, P17844, Q9Y333, P23246, Q9BWJ5, Q13838, Q15427, Q9H2H8, Q05519, Q15393, P62995, Q14103, Q15366	2.1710186
Annotation Cluster 23	***Enrichment Score: 2.5900919873172112***					
GOTERM_BP_FAT	GO:0044275~cellular carbohydrate catabolic process	9	2.4	0.001063	P40925, P11177, O95336, P52209, P00338, P06733, P11216, P46926, P17858	4.34053476
GOTERM_BP_FAT	GO:0046365~monosaccharide catabolic process	8	2.133333	0.001654	P40925, P11177, O95336, P52209, P00338, P06733, P46926, P17858	4.61903542
GOTERM_BP_FAT	GO:0006007~glucose catabolic process	7	1.866667	0.002762	P40925, P11177, O95336, P52209, P00338, P06733, P17858	4.94754441
GOTERM_BP_FAT	GO:0046164~alcohol catabolic process	8	2.133333	0.003521	P40925, P11177, O95336, P52209, P00338, P06733, P46926, P17858	4.04878414
GOTERM_BP_FAT	GO:0019320~hexose catabolic process	7	1.866667	0.006552	P40925, P11177, O95336, P52209, P00338, P06733, P17858	4.15880545

Annotation Cluster 24	***Enrichment Score: 2.4836261810505076***					
UP_SEQ_FEATURE	short sequence motif:Effector region	8	2.133333	0.002627	P11233, P60953, P61026, Q9BZG1, Q96AH8, P20340, Q13636, P51148	4.29204211
SMART	SM00175:RAB	6	1.6	0.003447	P61026, Q9BZG1, Q96AH8, P20340, Q13636, P51148	5.83670845
UP_SEQ_FEATURE	lipid moiety-binding region:S-geranylgeranyl cysteine	8	2.133333	0.00391	P11233, P60953, P61026, Q9BZG1, Q96AH8, P20340, Q13636, P51148	3.9974902

Annotation Cluster 25	***Enrichment Score: 2.4413368357204157***					
GOTERM_BP_FAT	GO:0006769~nicotinamide metabolic process	6	1.6	0.0027	P43490, P40925, O95336, P52209, P50213, O15229	6.14909091
GOTERM_BP_FAT	GO:0046496~nicotinamide nucleotide metabolic process	6	1.6	0.0027	P43490, P40925, O95336, P52209, P50213, O15229	6.14909091
GOTERM_BP_FAT	GO:0009820~alkaloid metabolic process	6	1.6	0.003015	P43490, P40925, O95336, P52209, P50213, O15229	5.99911308
GOTERM_BP_FAT	GO:0019362~pyridine nucleotide metabolic process	6	1.6	0.003356	P43490, P40925, O95336, P52209, P50213, O15229	5.85627706
GOTERM_BP_FAT	GO:0006733~oxidoreduction coenzyme metabolic process	6	1.6	0.008421	P43490, P40925, O95336, P52209, P50213, O15229	4.73006993
